# Photochemical Cu(iii)-mediated trifluoromethylation of (hetero)arenes and biomolecules

**DOI:** 10.1039/d5sc07405c

**Published:** 2025-11-11

**Authors:** Petr Pospíšil, Vladimir Motornov, Ondřej Michal, Lucie Šálková, Soňa Boháčová, Tomáš Slanina, Ján Tarábek, Blanka Klepetářová, Petr Beier

**Affiliations:** a Institute of Organic Chemistry and Biochemistry of the Czech Academy of Sciences Flemingovo nam. 2 16600 Prague Czech Republic beier@uochb.cas.cz cuprate51@gmail.com; b Institute of Organic Chemistry and Technology, Faculty of Chemical Technology, University of Pardubice Studentská 573 53210 Pardubice Czech Republic; c Department of Physical Chemistry, University of Chemistry and Technology, Prague Technická 5 16628 Prague Czech Republic; d Freie Universität Berlin Fabeckstraße 34-36 14195 Berlin Germany

## Abstract

A highly efficient and atom-economical method for the C–H trifluoromethylation of (hetero)arenes and complex biomolecules has been developed using a substoichiometric amount of the stable tetrakis(trifluoromethyl)cuprate(iii) salt. Upon violet-light irradiation in the presence of an oxidant, all four CF_3_ groups are sequentially converted into trifluoromethyl radicals, enabling high-yielding transformations under mild conditions. The protocol exhibits excellent functional group tolerance and is applicable to the late-stage trifluoromethylation of pharmaceuticals, amino acids, and nucleosides. Mechanistic studies support a photoinitiated radical pathway and reveal the full utilization of the Cu(iii) species. The results presented advance the use of copper-mediated strategies for the sustainable incorporation of fluorine into complex molecules.

## Abstract 2

Atom-economical trifluoromethylation of (hetero)arenes, including selected biomolecules, was achieved using a substoichiometric amount of a Cu(iii) trifluoromethyl complex.

## Graphical abstract

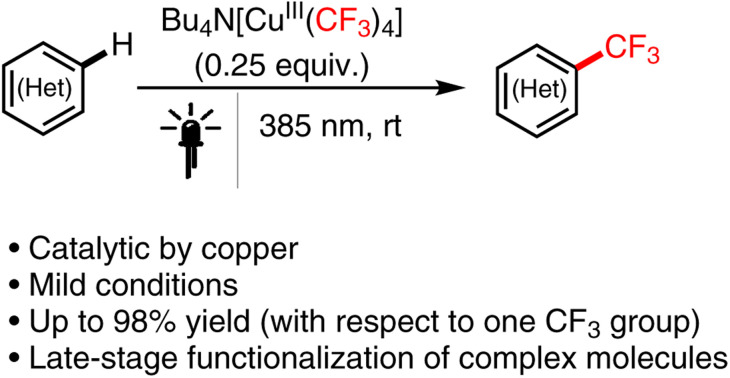

## Introduction

**Scheme 1:**
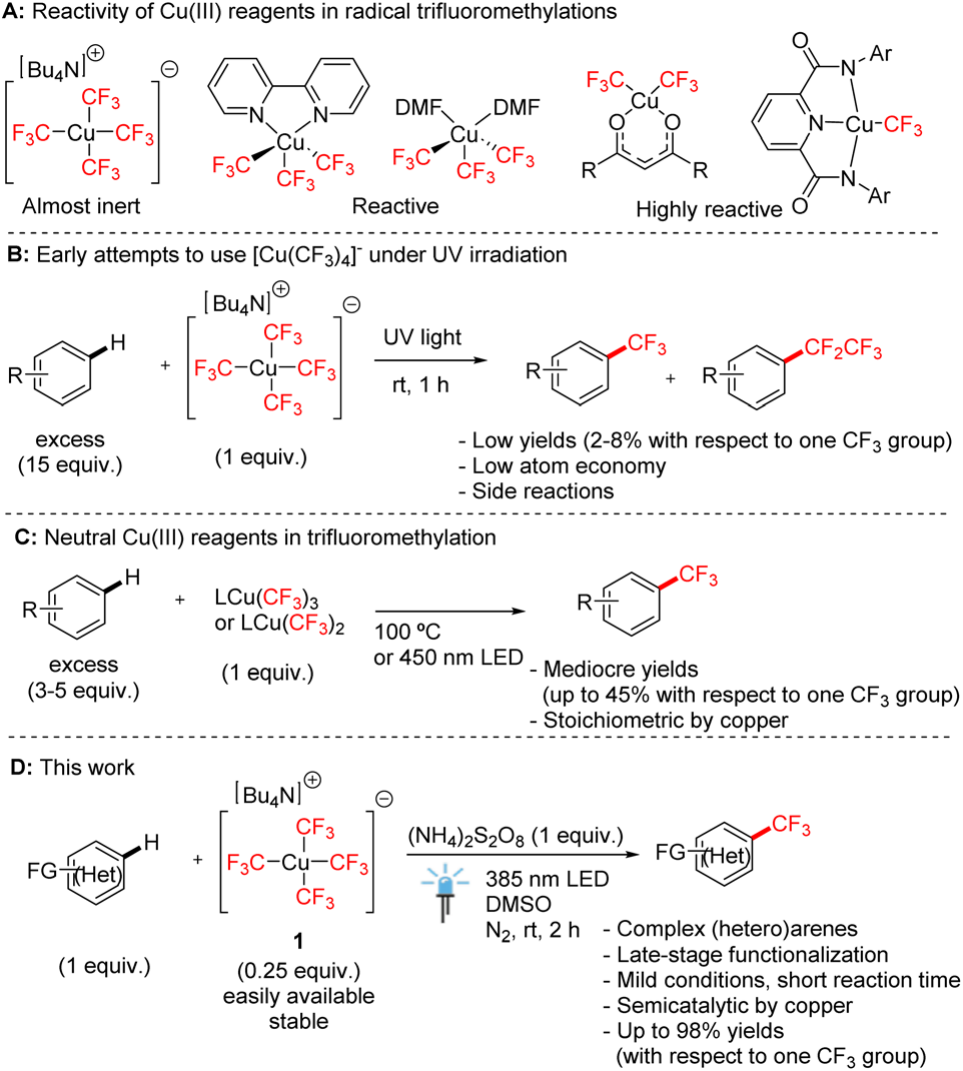
Reactivity of Cu(iii) complexes in aromatic C–H trifluoromethylation.

Cu(iii) trifluoromethyl compounds have received wide interest from organic and inorganic chemists due to their exceptional stability and versatile reactivity (Scheme 1A).^1^ Since the breakthrough discovery by Grushin in 2015, who described a simple procedure for the preparation of tetrakis(trifluoromethyl)cuprate salts [Cu(CF_3_)_4_]^−^ from CuCl with air as the only oxidant,^2^ Cu(iii) trifluoromethyl complexes have become candidates for applications in organic synthesis, especially as trifluoromethylation agents.^1*a*^ However, homoleptic tetrakis(trifluoromethyl)-cuprate(iii) salts are known to be poor trifluoromethylation agents. They have been shown to be activated to release trifluoromethyl radicals by harsh UV irradiation with only low efficiency (Scheme 1B),^3^ or by electrospray ionization.^4^ Therefore, neutral complexes with bidentate *N*-donor ligands, such as (bpy)Cu(CF_3_)_3_ (ref. 5) and (phen)Cu(CF_3_)_3_,^6^ are the most explored copper(iii) trifluoromethyl transfer reagents, even though they exhibit limited atom economy in trifluoromethylation.

We have recently described oxygen-donor solvate complexes (DMF)_2_Cu(CF_3_)_3_,^7^ and bis(trifluoromethyl)-1,3-diketonates^8^ bearing two trifluoromethyl groups which showed high reactivities in trifluoromethylation reactions. Moreover, a highly reactive Cu(iii) complex with only one trifluoromethyl group stabilized by a pyridine-2,6-dicarboxamide ligand has recently been reported.^9^ All these complexes are used as versatile stoichiometric C–H trifluoromethylation reagents under mild conditions (Scheme 1C), the reactivity of which increases with the decreasing number of CF_3_ ligands. However, no reported transformation starting from Cu(iii) trifluoromethyl complexes to date achieved the complete utilization of all trifluoromethyl groups in reactions, highlighting the need for an improved atom efficiency. For example, the yields of trifluoromethylation based on one trifluoromethyl group do not exceed 45% even with the most reactive complexes^7^ and it is extremely difficult to make use of all CF_3_ ligands in the complex. We aimed to develop a new strategy that overcomes this limitation. Given that the Bu_4_N[Cu(CF_3_)_4_] salt is the most stable and inexpensive Cu(iii) trifluoromethyl species,^2,7^ a way to utilize it with efficiency would be highly desirable.

Recently, we reported the cleavage of this homoleptic anion by Brønsted acids such as triflic acid, resulting in the formation of solvated Cu(CF_3_)_3_ species.^7^ However, this homoleptic anion is still commonly deemed poorly reactive in radical cleavage and trifluoromethylation.^10^ Herein, we report a new method of mild light-mediated trifluoromethylation of arenes and heteroarenes, including biomolecules with Bu_4_N[Cu(CF_3_)_4_] in substoichiometric amount (0.25 equiv. of copper) to make use of all four trifluoromethyl groups (Scheme 1D).

## Results and discussion

We commenced our studies with attempts to trifluoromethylate 1,3,5-trimethoxybenzene (2), a typical electron-rich arene, with cuprate salt 1 in the most atom-efficient manner possible, employing light as a cost-efficient energy source (Table 1). We were intrigued by the phenomenon of photoinduced homolytic cleavage of Cu(iii) trifluoromethyl complexes by blue monochromatic LED light of wavelengths far above their absorption maxima, which is attributed to spin-forbidden HOMO to LUMO + 1 excitation.^5*c*,7^ Therefore, we attempted to use an LED source of violet 385 nm light to excite the tetrakis(trifluoromethyl)-cuprate anion in the presence of a persulfate oxidant to facilitate the formation of CF_3_ radicals. To make use of all four trifluoromethyl groups, we employed a 4 : 1 ratio of arene/[Cu], which would indicate an improvement on the commonly employed stoichiometric Cu–CF_3_ reagent by making the reaction substoichiometric.

**Table 1:** Optimization of substoichiometric trifluoromethylation of 1,3,5-trimethoxybenzene^a^

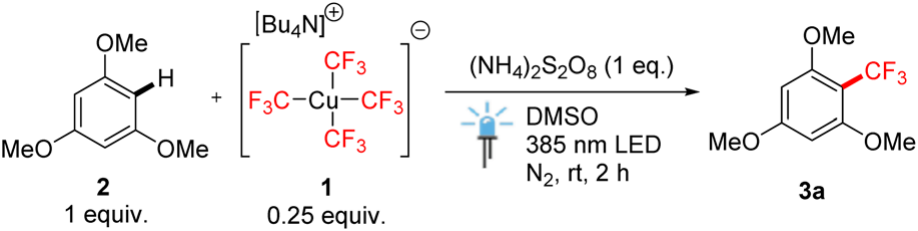		
Entry	Deviation from standard conditions	Yield (%)^b^
1	None	**88** (352)
2	MeCN as the solvent	49 (294)
3	Under air	81 (325)
4	K_2_S_2_O_8_ as the oxidant	84 (337)
5	Na_2_S_2_O_8_ as the oxidant	81 (324)
6	0.125 equiv. of [Cu]	50 (**400**)
7	No light, 275 nm or 465 nm LED light	<5
8	No oxidant	<10
9	With Ph_4_P[Cu(CF_3_)_4_] salt	75 (298)

a Reaction conditions: 2 (0.4 mmol), 1 (0.25 equiv.), solvent (1 ml), rt, 385 nm LED light, 2 h.

b Yields were determined by ^19^F NMR with PhCF_3_ as an internal standard and are based on one CF_3_ group of 1; values in parentheses indicate yields with respect to copper.

After extensive optimization of solvents, oxidants and the reagent ratio (see the SI for full details), we achieved the trifluoromethylation of 2 in 88% yield with respect to the arene and one trifluoromethyl group, which equals to a yield of 352% with respect to the Cu(iii) complex (Table 1, entry 1). Switching from dimethylsulfoxide to acetonitrile as the solvent decreased the yield (entry 2), and potassium or sodium persulfates turned out to be slightly less efficient oxidants than ammonium persulfate (entries 4 and 5). The model reaction was only slightly affected by the presence of air (entry 3). Importantly, when the cuprate salt was used in 0.125 equiv. (8 : 1 arene/[Cu] ratio), the CF_3_ groups reacted with the substrate quantitatively (400% yield based with respect to copper, entry 6). Control experiments demonstrated that the trifluoromethylation was suppressed in the absence of light or if different irradiation wavelengths were employed, as well as in the absence of an oxidant (entries 7 and 8). The use of tetraphenyl-phosphonium cuprate salt resulted in a slightly lower yield compared to the initially used tetrabutylammonium salt 1 (entry 9).

**Scheme 2:**
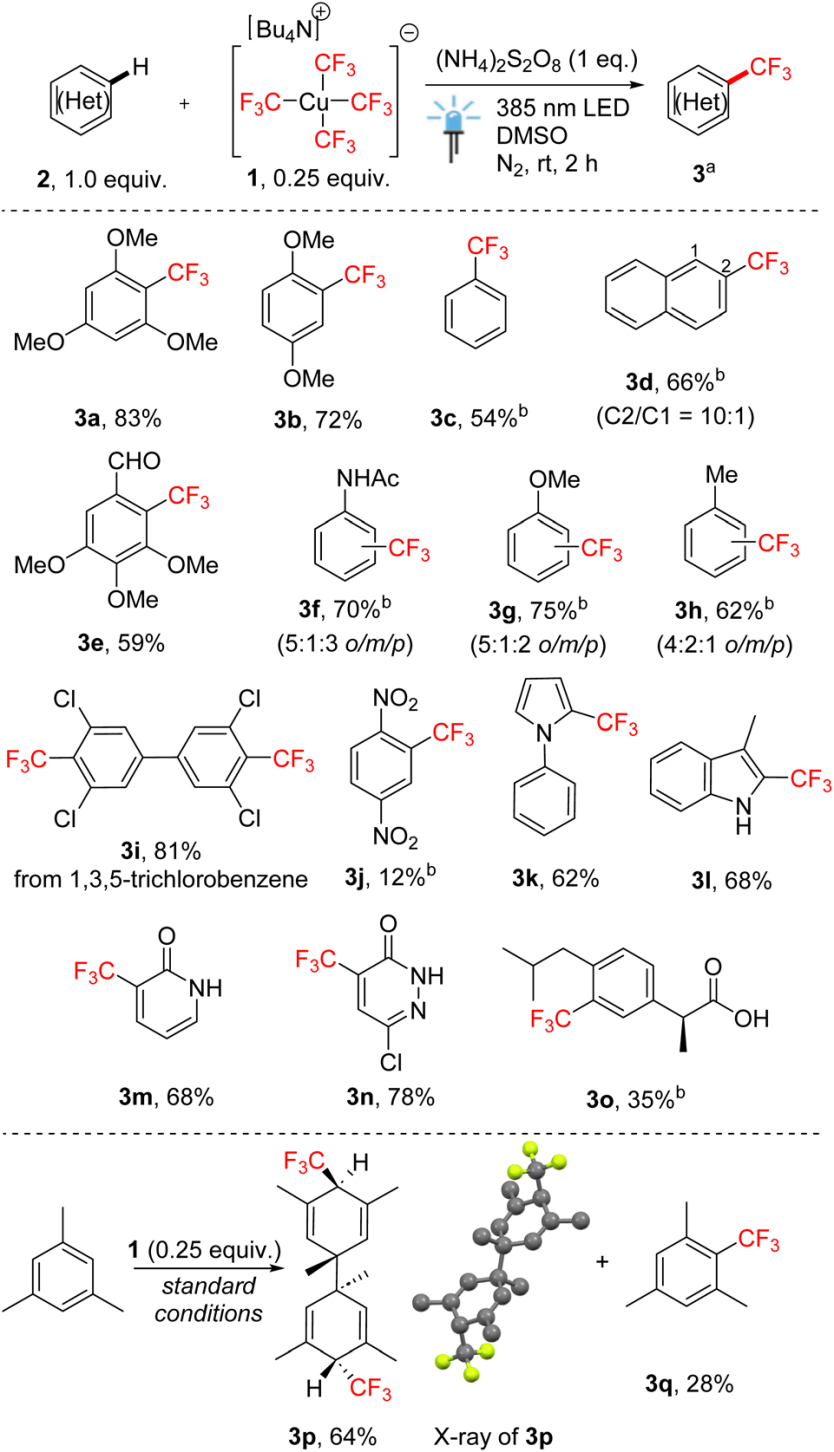
Scope of trifluoromethylation of arenes and heteroarenes 2 with tetrakis(trifluoromethyl)cuprate(iii) salt 1. ^a^Isolated yields after column chromatography, unless stated otherwise. ^b 19^F NMR yield.

Under the optimized conditions (Table 1, entry 1), we investigated the method's potential scope of applicability on simple arenes (Scheme 2). High product yields were obtained for electron-rich symmetrical tri- and dimethoxybenzenes (products 3a and 3b). Unsubstituted benzene afforded a moderate yield of product 3c, which matched the reactivity tendency of the electrophilic CF_3_ radical. Naphthalene predominantly afforded compound 3d produced by β-trifluoromethylation. The method tolerated the presence of the formyl group (product 3e) or the acetamide moiety (3f). Monosubstituted electron-rich arenes mainly afforded mixtures of regioisomers in moderate to good yields (3f–3h), whereas chlorinated biphenyl derivative 3i formed from 1,3,5-trichlorobenzene by radical addition, dimerization, and HCl elimination. The highly electron-deficient substrate dinitrobenzene underwent trifluoromethylation with rather low efficiency (product 3j). Finally, nitrogen heterocycles such as *N*-phenylpyrrole, 3-methylindole, 2-pyridone and 6-chloropyridazin-3(2*H*)-one were tolerated giving α-trifluoromethylation products 3k–3n in good yields. Ibuprofen can be also trifluoromethylated in comparatively lower yield. It is worth mentioning that mesitylene, under standard conditions, predominantly afforded the product of radical addition and dimerization (3p), the structure of which has been confirmed by X-ray crystallography. This outcome indirectly supports a radical pathway. Analogous dimerization has been reported previously, although in a different context.^13^

**Scheme 3:**
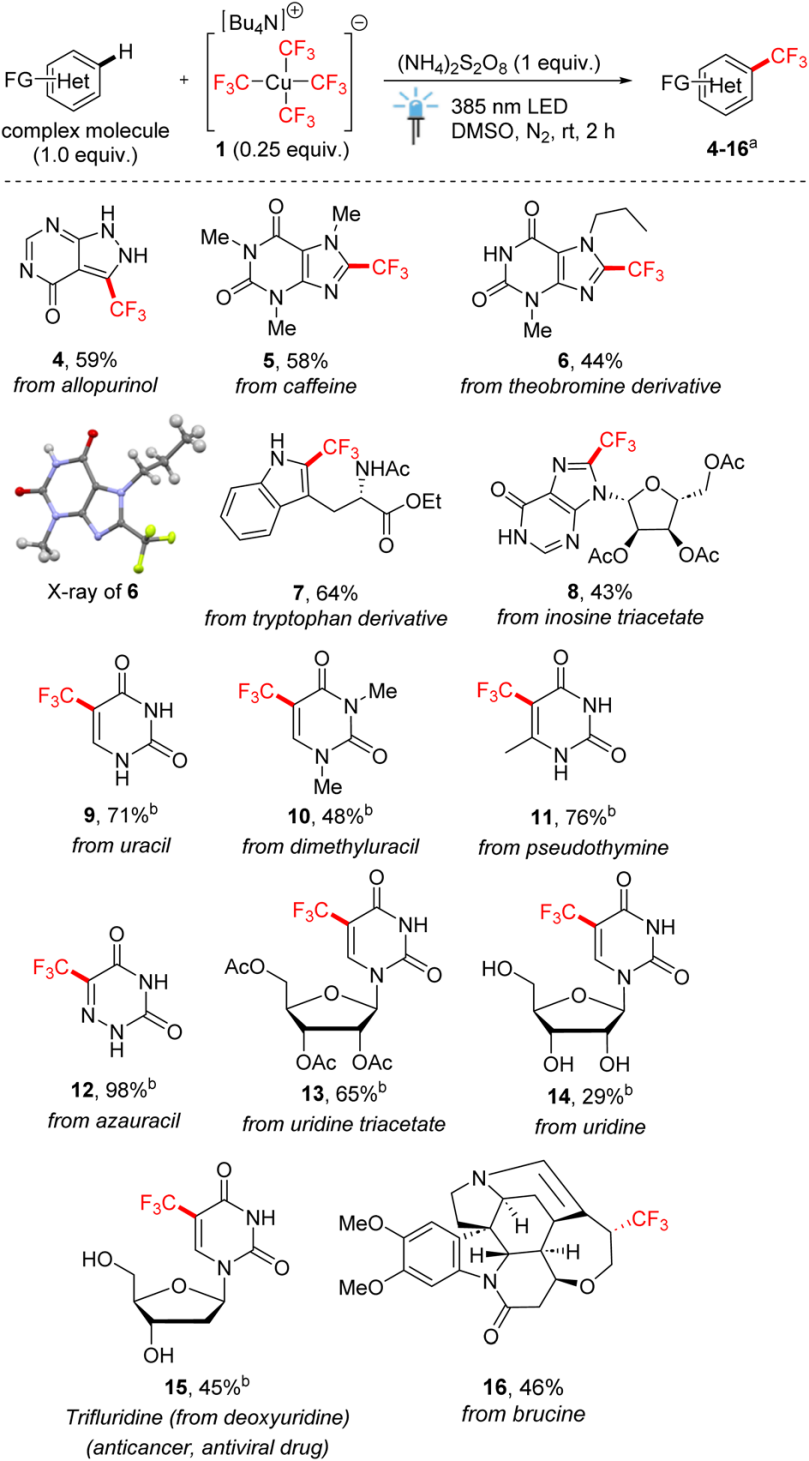
Late-stage trifluoromethylation of complex heterocycles with 1 (0.25 equiv.). ^a^Isolated yields after column chromatography. ^b^DMSO/H_2_O (3 : 1) as the solvent and Na_2_S_2_O_8_ as the oxidant.

Encouraged by the excellent atom economy and good functional group tolerance of the method, we tested the applicability of copper-mediated trifluoromethylation to the late-stage functionalization of more complex heterocycles (Scheme 3). The anti-gout medication allopurinol was trifluoromethylated smoothly under standard conditions in good yield (product 4). The procedure also proved applicable to xanthine derivatives, such as caffeine and *N*-propylated theobromine derivative (products 5 and 6). Tryptophan ester bearing amide and indole moieties afforded product 7 also in a good yield. Next, trifluoromethylation of nucleosides and nucleobases was explored. In some cases, adding water as a co-solvent improved the solubility of substrates with unprotected hydroxy groups, demonstrating the water-friendly nature of the present method. Inosine triacetate underwent site-selective trifluoromethylation of the fused imidazole ring (product 8). Uracil and its derivatives smoothly underwent trifluoromethylation to give products 9–11. Azauracil, which is an important inhibitor of DNA synthesis, afforded product 12 in excellent yield. Notable results were obtained for uridine nucleosides, including the synthesis of the well-established anticancer and antiviral drug trifluridine (15) from deoxyuridine as the starting substrate. Finally, brucine was trifluoromethylated in a stereoselective fashion.

**Fig. 1:**
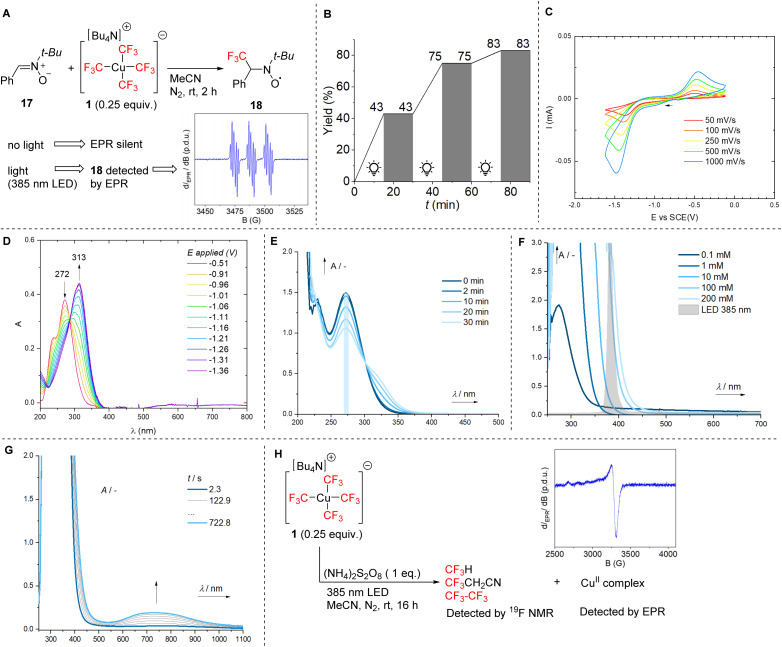
Mechanistic studies of trifluoromethylation with 1. A: EPR detection of the trifluoromethyl radical. B: Lights on/off experiment with a model reaction of trifluoromethylation of 1,3,5-trimethoxybenzene with 1. C: Cyclic voltammetry of 1 (1 mM) in 0.1 M Bu_4_NPF_6_ in MeCN at different scan rates (glassy carbon working electrode). D: Spectroelectrochemical investigation of the reduction of 1. E: Absorption spectra upon irradiation of 1. F: Concentration-dependent absorption spectra of 1. G: Absorption spectra during trifluoromethylation of 1,3,5-trimethoxybenzene with 1 according to standard conditions. H: EPR detection of a square planar Cu(ii) complex.

Having gathered data on a range of trifluoromethylated arenes and heteroarenes, including some prominent biomolecules, we gained further insight into the mechanism of the trifluoromethylation reaction presented. To confirm the key role of the trifluoromethyl radical in this transformation, we performed electron paramagnetic resonance (EPR) analysis of 1 in the presence of spin trap 17 (Fig. 1A). Trifluoromethyl radical adduct 18 was detected by EPR under light irradiation and only an extremely low EPR signal intensity was observed in the absence of light. The crucial role of the light was additionally supported by an on/off experiment (Fig. 1B), in which the reaction was completely shut down upon turning off the irradiation.

The reduction potential of 1 was determined by cyclic voltammetry (*E*_pc_*vs.* SCE = −1.42 V, Fig. 1C). Spectroelectro-chemical analysis of the reduction of 1 showed a decrease in absorbance at 272 nm and the formation of a new absorption band (*λ*_max_ = 313 nm), with a clear isosbestic point (Fig. 1D). A similar phenomenon was observed by absorption spectroscopy, where the absorption at 313 nm increased after irradiation of 1 at its *λ*_max_, albeit with a lower conversion (Fig. 1E). Although absorption of compound 1 shows no overlap with the LED light source used in the trifluoromethylation process (385 nm) at low concentrations, at higher concentrations it exhibits significant absorbance, enabling an effective photoreaction (Fig. 1F). The preparative trifluoromethylation reactions were typically performed at a 0.1 M concentration of 1. Real-time analysis of the absorption spectra during the trifluoromethylation of 1,3,5-trimethoxybenzene with 1 revealed the gradual formation of a new broad band at 600–900 nm (Fig. 1G). This corresponds most likely to the Cu(ii)-CF_3_ species^11^ and Cu(ii) by-products, given that all CF_3_ groups capable of stabilizing Cu(iii) and Cu(i) are consumed in the reaction. EPR analysis following the reaction of 1 in MeCN with the oxidant in the absence of an arene substrate indicated the formation of a Cu(ii) complex (most likely symmetrical square planar), and ^19^F NMR analysis showed the formation of fluoroform, 3,3,3-trifluoropropionitrile, and hexafluoroethane arising from the reaction of trifluoromethyl radicals with hydrogen atoms or fragments of the homolyzed solvent, and dimerization respectively (Fig. 1H).

**Scheme 4:**
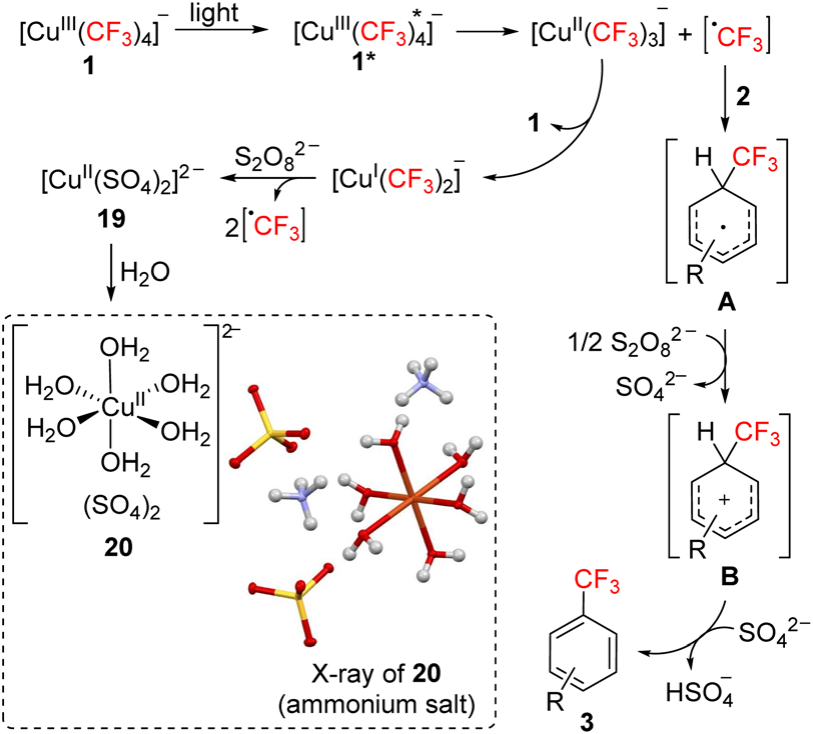
Proposed reaction mechanism of trifluoromethyla-tion of arenes 2 with Bu_4_N[Cu(CF_3_)_4_] (1).

Based on these observations, we propose that the described trifluoromethylation proceeds by the following mechanism (Scheme 4). Excitation of cuprate salt 1 produces its excited complex 1*, which decomposes into a trifluoromethyl radical and a [Cu^ii^(CF_3_)_3_]^−^ intermediate. Radical addition to 2 affords radical intermediate A, which upon oxidation to cationic intermediate B and proton transfer to a sulfate anion furnishes product 3. The [Cu^ii^(CF_3_)_3_]^−^ intermediate can disproportionate to regenerate 1 and form bis(trifluoromethyl)cuprate(i) anion. Oxidation of the Cu(i) species releases the remaining two trifluoromethyl radicals and generates a square-planar Cu(ii) sulfate complex 19, corresponding to the complex observed by EPR. The addition of water enabled the isolation of product 20, a known^12^ light-blue (hence the absorption at 600–900 nm) octahedral hexa–aqua complex 20 that has been characterized by X-ray crystallography (Scheme 4).

## Conclusions

We have developed an efficient and atom-economical method for the C–H trifluoromethylation of (hetero)arenes, including biomolecules, using the readily accessible and stable tetrakis(trifluoromethyl)cuprate(iii) salt in substoichiometric amount. This light-driven protocol enables the full utilization of all four CF_3_ groups, overcoming a long-standing limitation of copper(iii) trifluoromethylation chemistry. The reactions proceed under mild conditions and exhibit excellent functional group tolerance, enabling the late-stage functionalization of pharmaceuticals and nucleosides. Mechanistic studies provide evidence that the reactions take place through a radical pathway. These findings expand the synthetic utility of Cu(iii) species and set the stage for their future application in organic synthesis.

## Author contributions

P. P. and V. M. conceived the idea, performed experiments and partially wrote the manuscript, O. M. performed experiments, L. Š. and S. B. performed photochemical and electrochemical measurements, T. S. partially wrote the manuscript, J. T. performed EPR measurements, B. K. performed X-ray analysis, P. B. led the project, obtained funding and partially wrote the manuscript. All authors have given approval to the final version of the manuscript.

## Conflicts of interest

There are no conflicts to declare.

## Supplementary Material

SC-OLF-D5SC07405C-s001

SC-OLF-D5SC07405C-s002

## Data Availability

The data supporting this article have been included as part of the supplementary information (SI). Supplementary information is available. See DOI: https://doi.org/10.1039/d5sc07405c. CCDC 2474866–2474868 contain the supplementary crystallographic data for this paper.^14^
