# Supplementary material for: Photochemical Cu(iii)-mediated trifluoromethylation of (hetero)arenes and biomolecules
Source: Chem Sci. 2025 Nov 11;17(1):430–5. doi: 10.1039/d5sc07405c (PMC12620705; doi:10.1039/d5sc07405c)

Supplementary Information (SI) for Chemical Science.
This journal is © The Royal Society of Chemistry 2025

Supplementary material for

Photochemical Cu(III)-mediated trifluoromethylation of (hetero)arenes and biomolecules

Petr Pospíšil,[a,b] Vladimir Motornov,[a] Ondřej Michal,[a,c] Lucie Wohlrábová,[a,c] Soňa Boháčová,[a] Tomáš Slanina,[a] Ján Tarábek,[a] Blanka Klepetářová[a] and Petr Beier[a]

[a] Institute of Organic Chemistry and Biochemistry of the Czech Academy of Sciences, Flemingovo náměstí 2, 166 00 Prague 6, Czech Republic

[b] Institute of Organic Chemistry and Technology, Faculty of Chemical Technology, University of Pardubice, Studentská 573, Pardubice, 532 10, Czech Republic

[c] Department of Physical Chemistry, University of Chemistry and Technology, Prague, Technická 5, 166 28 Prague, Czech Republic

Content

General information S2
Synthesis of tetrakis(trifluoromethyl)cuprate(III) S4
Optimization of reaction conditions S4
General procedure: Synthesis of trifluoromethylated arenes and heteroarenes S6
Characterization data of trifluoromethylated products S6
Isolation of ammonium copper(II) sulfate hexahydrate S14
UV-Vis and fluorescence spectroscopy S15
Cyclic voltammetry S20
Photo-EPR studies S26
Crystallographic data S33
References S37
Copies of NMR spectra S39

General information

Reactions with air-sensitive materials were carried out under nitrogen atmosphere using standard Schlenk techniques. All solvents were dried by activated molecular sieves (3 Å) and stored under nitrogen. All commercially available chemicals were used as received unless stated otherwise. Flash column chromatography was performed using silica gel 60 (230-400 mesh particle size). Automated flash column chromatography was performed on Teledyne ISCO CombiFlash Rf+ Lumen Automated Flash Chromatography System with UV/Vis detection. ^{1}H, ^{13}C, and ^{19}F NMR spectra were measured at ambient temperature using 5 mm diameter NMR tubes. ^{13}C NMR spectra were proton decoupled. The chemical shift values (δ) are reported in ppm relative to internal Me_4Si (0 ppm for ^{1}H and ^{13}C NMR) or residual solvents and internal $CFCl_3$ (0 ppm for ^{19}F NMR). Coupling constants (J) are reported in Hertz. Structural elucidation was aided by the additional acquisition of various 2D spectra (^{1}H-^{1}H COSY, ^{1}H-^{13}C HSQC, ^{1}H-^{13}C HMBC). GCMS spectra were recorded on a gas chromatograph coupled with a quadrupole mass-selective electron impact (EI) detector (70 eV). High resolution MS spectra (HRMS) were recorded on a Waters Micromass AutoSpec Ultima or an Agilent 7890A GC coupled with a Waters GCT Premier orthogonal acceleration time-of-flight detector using electron impact (EI) ionization or chemical ionization (CI), Q-Tof micro (Waters) is a quadrupole orthogonal acceleration time-of-flight tandem mass spectrometer using atmospheric-pressure chemical ionization (APCI). UV-vis spectrometry was measured using Agilent Cary 8454 UV-vis (Agilent). As light source LED lamps were used: LED385 (λ_{max} = 387 nm, P = 5 W, Figure S1).

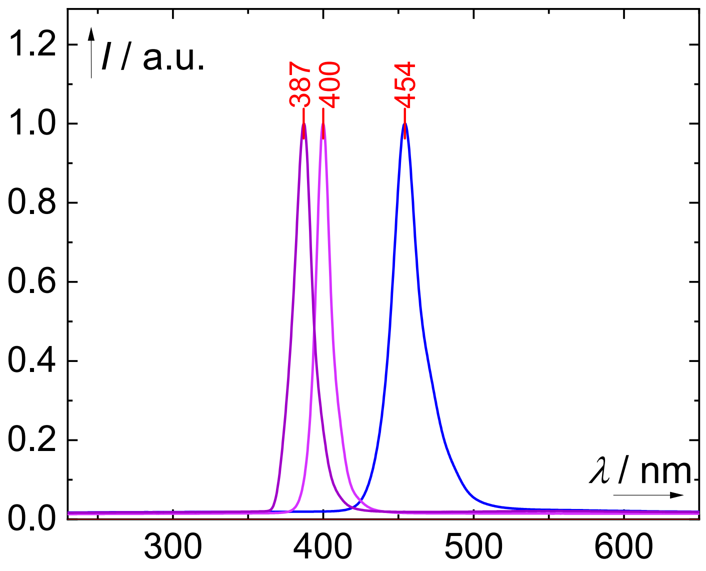

Figure S1. Normalized emission spectra of the used LED light sources

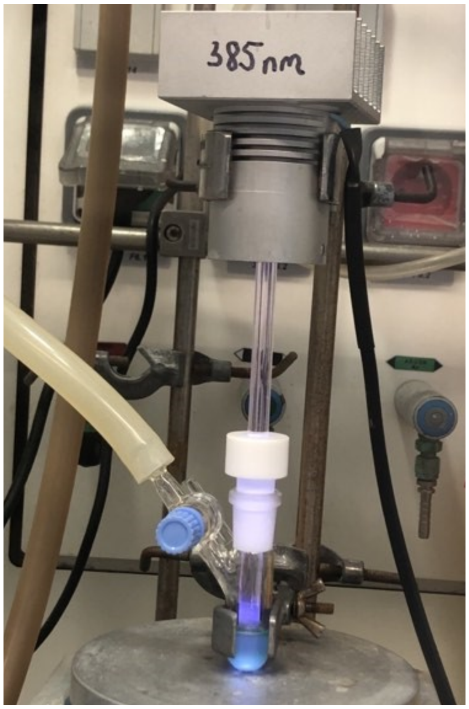

Figure S2. Reaction setup.

Synthesis of tetrakis(trifluoromethyl)cuprate(III)

Tetrakis(trifluoromethyl)cuprate(III) (**1**) was prepared according to the procedure published in the literature.[1]

Optimization of reaction conditions

Table S1. Screening reaction conditions in trifluoromethylation of 1,3,5-trimethoxybenzene with **1**.

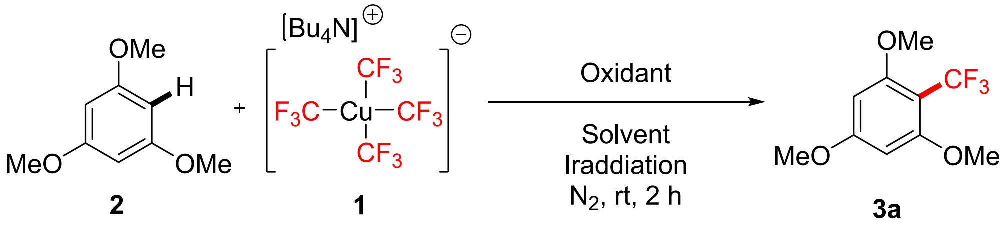

Entry	Solvent	Equiv. of **2**	Oxidant (equiv.)	λ LED (nm)	Yield of **3a**[a] (%)
1	MeCN	6	$(NH_4)_2S_2O_8$ (6)	darkness	0
2	MeCN	6	$(NH_4)_2S_2O_8$ (6)	275	0
3	MeCN	6	$(NH_4)_2S_2O_8$ (6)	455–475	2
4	DMF	5	$(NH_4)_2S_2O_8$ (5)	385	26 (132)
5	MeCN	6	$Na_2S_2O_8$ (6)	385	10 (60)
6	MeCN	6	$K_2S_2O_8$ (6)	385	35 (211)
7	MeCN	6	PIDA (6)	385	30 (181)
8	MeCN	6	Oxone (6)	385	22 (133)
9	MeCN	6	$(NH_4)_2S_2O_8$ (6)	385	49 (294)
10	MeCN	4	$(NH_4)_2S_2O_8$ (4)	385	58 (232)
11	MeCN	5	$(NH_4)_2S_2O_8$ (2)	385	6 (31)
12	DMSO	4	$(NH_4)_2S_2O_8$ (4)	385	88 (352)
13	DMSO	8	$(NH_4)_2S_2O_8$ (8)	385	50 (400)
14	DMSO	4	$Na_2S_2O_8$ (4)	385	81 (324)
15	DMSO	4	$K_2S_2O_8$ (4)	385	84 (337)
16	DMSO/water (3:1)	4	$(NH_4)_2S_2O_8$ (4)	385	75 (299)

Reaction conditions: **1** (0.1 mmol, 1 equiv.), solvent (1 ml), rt, 385 nm LED, 2 h. [a] Yields were determined by ^{19}F NMR with $PhCF_3$ as an internal standard and are based on one CF_3 group of **1**, in parentheses yields based on copper.

Table S2. Cation screening in trifluoromethylation of 1,3,5-trimethoxybenzene with **1**.

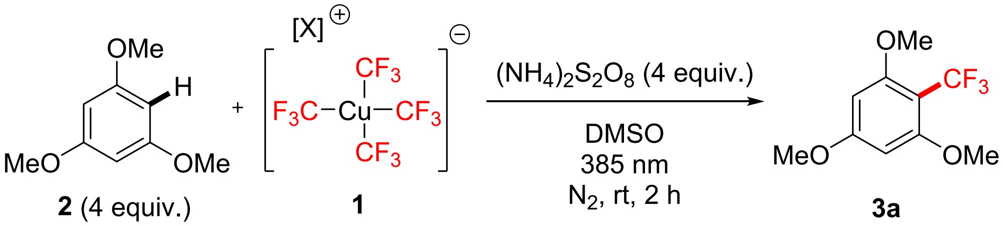

Entry	Cation [X]	Yield of **3a**[a] (%)
1	NBu_4	88 (352)
2	PPh_4	75 (298)

Reaction conditions: **1** (0.1 mmol, 1 equiv.), solvent (1 ml), rt, 385 nm LED, 2 h. [a] Yields were determined by ^{19}F NMR with $PhCF_3$ as an internal standard and are based on one CF_3 group of **1**, in parentheses yields based on copper.

Table S3. Atmosphere screening in trifluoromethylation of 1,3,5-trimethoxybenzene with **1**.

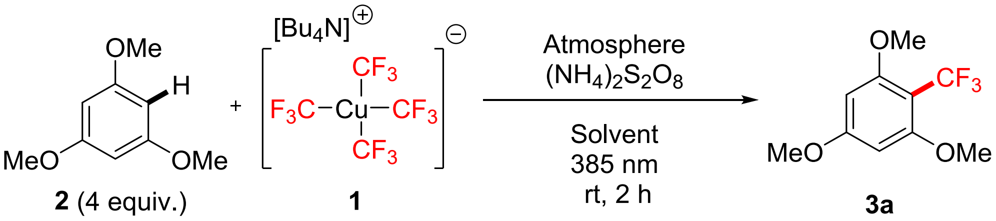

Entry	Solvent	Atmosphere	Oxidant (eq.)	Yield of **3a**[a] (%)
1	MeCN	Nitrogen	6	49 (294)
2	MeCN	Air	6	15 (90)
3	DMSO	Nitrogen	4	88 (352)
4	DMSO	Air	4	81 (325)

Reaction conditions: **1** (0.1 mmol, 1 equiv.), solvent (1 ml), rt, 385 nm LED, 2 h. [a] Yields were determined by ^{19}F NMR with $PhCF_3$ as an internal standard and are based on one CF_3 group of **1**, in parentheses yields based on copper.

General procedure: Synthesis of trifluoromethylated arenes and heteroarenes

An oven-dried Schlenk flask was charged with tetrabutylammonium tetrakis(trifluoromethyl)cuprate(III) (59 mg, 0.1 mmol, 0.25 equiv.), substrate (0.4 mmol, 1 equiv.) and $(NH_4)_2S_2O_8$ (92 mg, 0.4 mmol, 1 equiv.) then it was evacuated and backfilled with nitrogen (3×). DMSO (1 ml) was added under inert atmosphere. LED light (λ_{max} = 387 nm) (see Figure S2) was introduced into the flask via a glass rod and after sealing under nitrogen atmosphere the solution was irradiated for 2 h at room temperature. The reaction was followed by ^{19}F NMR. DCM or EtOAc was added, and the organic layer was washed with water (2×) and with brine (1×). The organic layer was dried over Na_2SO_4 and purified by column chromatography (silica gel).

Characterization data of trifluoromethylated products

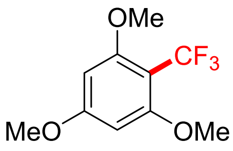

1,3,5-Trimethoxy-2-(trifluoromethyl)benzene (**3a**): Prepared from 1,3,5-trimethoxybenzene following the *General procedure*. Column chromatography (hexane/EtOAc, 5:1) afforded pure product as a white solid (91 mg, 83%). ^{1}H NMR (400 MHz, $CDCl_3$) δ 6.13 (s, 2H), 3.83 (s, 9H). ^{13}C {^{1}H} NMR (101 MHz, $CDCl_3$) δ 163.6, 160.5 (q, J = 1.5 Hz), 124.5 (q, J = 273.3 Hz), 100.5 (q, J = 30.1 Hz), 91.4, 56.4, 55.5. ^{19}F NMR (376 MHz, $CDCl_3$) δ –54.62 (s). HRMS (EI, *m/z*) Calcd for $C_{10}H_{11}F_3O_3$ $[M]^+$: 236.0655; Found: 236.0656. The above analytical data agreed with previously published data.[2]

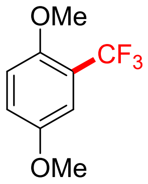

1,4-Dimethoxy-2-(trifluoromethyl)benzene (**3b**): Prepared from 1,4-dimethoxybenzene following the *General procedure*. Column chromatography (hexane/DCM, 4:1) afforded pure product as a colorless oil (59 mg, 72%). ^{1}H NMR (400 MHz, $CDCl_3$) δ 7.12 (d, J = 3.1 Hz, 1H), 7.02 (dd, J = 9.0, 3.1 Hz, 1H), 6.94 (d, J = 9.0 Hz, 1H), 3.86 (s, 3H), 3.79 (s, 3H). ^{13}C {^{1}H} NMR (101 MHz, $CDCl_3$) δ 153.1, 151.7 (q, J = 1.8 Hz), 123.6 (q, J = 272.5 Hz), 119.5 (q, J = 31.0 Hz), 118.2, 113.7, 113.0 (q, J = 5.4 Hz), 56.7, 56.0. ^{19}F NMR (376 MHz, $CDCl_3$) δ –62.92 (s). HRMS (EI, *m/z*) Calcd for $C_9H_9F_3O_2$ $[M]^+$: 206.0549; Found: 206.0548. The above analytical data agreed with previously published data.[3]

(Trifluoromethyl)benzene (**3c**): Prepared from benzene following the *General procedure*. Crude

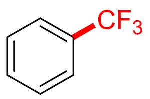

product 54% ^{19}F NMR yield. ^{19}F NMR (376 MHz, $CDCl_3$) δ –63.18 (s). The above analytical data agreed with previously published data.[4]

2-(Trifluoromethyl)naphthalene (**3d**) and *1-(trifluoromethyl)naphthalene* (**3d***): Prepared from

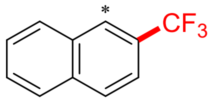

naphthalene following the *General procedure*. **3d:**3**d*** = 10:1. Crude product 66% ^{19}F NMR yield. **3d**: ^{19}F NMR (376 MHz, $CDCl_3$): δ –60.21 (s). **3d***: ^{19}F NMR (376 MHz, $CDCl_3$): δ –59.92 (s). The above analytical data agreed with previously published data.[3]

3,4,5-Trimethoxy-2-(trifluoromethyl)benzaldehyde (**3e**): Prepared from 3,4,5-

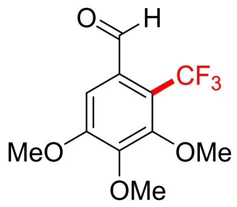

trimethoxybenzaldehyde following the *General procedure*. Column chromatography (hexane/EtOAc, 5:1) afforded pure product as a white solid (62 mg, 59%). ^{1}H NMR (400 MHz, $CDCl_3$) δ 10.31 (q, J = 2.5 Hz, 1H), 7.33 (s, 1H) 3.94 (s, 6H), 3.93 (s, 3H). ^{13}C {^{1}H} NMR (101 MHz, $CDCl_3$) δ 189.3 (q, J = 6.1 Hz), 155.9, 152.9 (q, J = 2.4 Hz) 147.4, 131.0, 124.3 (q, J = 275.1 Hz), 118.0 (q, J = 31.3 Hz), 107.4, 62.1, 61.1, 56.4. ^{19}F NMR (376 MHz, $CDCl_3$) δ –51.59 (d, J = 2.7 Hz). HRMS (EI, m/z) Calcd for $C_{11}H_{11}F_3O_4$ $[M]^+$: 264.0604; Found: 264.0608. The above analytical data agreed with previously published data.[2]

2-(Trifluoromethyl)acetanilide (**3f**^{1}), *3-(trifluoromethyl)acetanilide* (**3f**^{2}), *4-(trifluoromethyl)-acetanilide* (**3f**^{3})

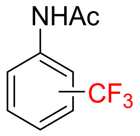

and *4-(trifluoromethyl)acetanilide* (**3f**^{3}): Prepared from *N*-phenylacetamide following the *General procedure*. **3f**^{1}**: 3f**^{2}:**3f**^{3} = 5:1:3. Crude product 70%. ^{19}F NMR yield. **3f**^{1}: ^{19}F NMR (376 MHz, $CDCl_3$): δ –61.14 (s). **3f**^{2}: ^{19}F NMR (376 MHz, $CDCl_3$): δ –63.27 (s). **3f**^{3}: ^{19}F NMR (376 MHz, $CDCl_3$): δ –62.63 (s). The above analytical data agreed with previously published data.[2]

2-(trifluoromethyl)anisole (**3g**^{1}), *3-(trifluoromethyl)anisole* (**3g**^{2}), *4-(trifluoromethyl)anisole*

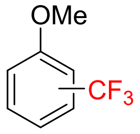

(**3g**^{3}): Prepared from anisole following the *General procedure*. **3g**^{1}**:3g**^{2}**:3g**^{3} = 5:1:2. Crude product 75%. ^{19}F NMR yield. **3g**^{1}: ^{19}F NMR (376 MHz, $CDCl_3$): δ –62.66 (s). **3g**^{2}: ^{19}F NMR (376 MHz, $CDCl_3$): δ –62.97 (s). **3g**^{3}: ^{19}F NMR (376 MHz, $CDCl_3$): δ –61.68 (s). The above analytical data agreed with previously published data.[5]

1-Methyl-2-(trifluoromethyl)benzene (**3h**[1]), 1*-methyl-3-(trifluoromethyl)benzene* (**3h**[2]), *1- methyl-4-(trifluoromethyl)benzene* (**3h**[3]): Prepared from toluene following the *General procedure*. **3h**[1]**: 3h**[2]:**3h**[3] = 4:1:2. Crude product 62% ^{19}F NMR yield. ^{19}F NMR yield. **3h**[1]: ^{19}F NMR (376 MHz, $CDCl_3$): δ –62.16 (s). **3h**[2]: ^{19}F NMR (376 MHz, $CDCl_3$): δ –62.74 (s). **3h**[3]: ^{19}F NMR (376 MHz, $CDCl_3$): δ –63.08 (s). The above analytical data agreed with previously published data.[5]

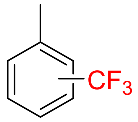

3,3',5,5'-Tetrachloro-4,4'-bis(trifluoromethyl)-1,1'-biphenyl (**3i**): Prepared from 1,3,5-trichlorobenzene following the *General procedure*. Column chromatography (pentane) afforded pure product as a white solid (139 mg, 81%). ^{1}H NMR (400 MHz, $CDCl_3$) δ 7.61 (q, J = 0.8 Hz, 4H). ^{13}C {^{1}H} NMR (101 MHz, $CDCl_3$) δ 141.2, 135.7 (q, J = 1.6 Hz), 129.2, 126.7 (q, J = 31.4 Hz), 122.4 (q, J = 276.5 Hz). ^{19}F NMR (376 MHz, $CDCl_3$) δ –56.38 (s). HRMS (EI, m/z) Calcd for $C_{14}H_4Cl_4F_6$ $[M]^+$: 425.8966; Found: 425.8980. The ^{1}H NMR data is in agreement with previously published data.[6]

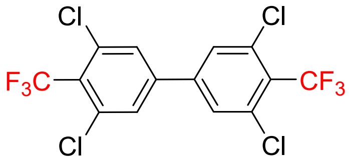

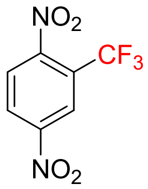

1,4-Dinitro-2-(trifluoromethyl)benzene (**3j**): Prepared from 1,4-dinitrobenzene following the *General procedure*. Crude product 12% ^{19}F NMR yield. ^{19}F NMR (376 MHz, $CDCl_3$) δ –58.81 (s). The above analytical data agreed with previously published data.[7]

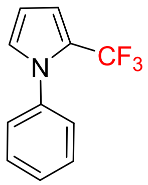

1-Phenyl-2-(trifluoromethyl)-1H-pyrrole (**3k**): Prepared from 1-phenyl-1*H*-pyrrole following the *General procedure*. Column chromatography (pentane) afforded pure product as a colorless liquid (52 mg, 62%). ^{1}H NMR (400 MHz, $CDCl_3$) δ 7.48–7.42 (m, 3H), 7.40–7.37 (m, 2H), 6.89 (t, J = 2.3 Hz, 1H), 6.74 (m, 1H), 6.28 (t, J = 3.3 Hz, 1H). ^{13}C {^{1}H} NMR (101 MHz, $CDCl_3$) δ 139.3, 129.1, 128.6, 127.4 (q, J = 2.1 Hz) 126.7, 125.8, 122.4 (q, J = 38.1 Hz), 121.3 (q, J = 266.9 Hz), 108.4. ^{19}F NMR (376 MHz, $CDCl_3$) δ –56.46. HRMS (EI, m/z) Calcd for $C_{11}H_8F_3N$ $[M]^+$: 211.0603; Found: 211.0601. The above analytical data agreed with previously published data.[8]

3-Methyl-2-(trifluoromethyl)-1H-indole (**3l**): Prepared from 3-methyl-1*H*-indole following the *General procedure*. Column chromatography (DCM/pentane, 1:1) afforded

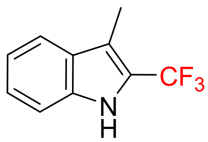

pure product as a white solid (54 mg, 68%). ^{1}H NMR (400 MHz, $CDCl_3$) δ 8.15 (br, 1H), 7.67 (d, J = 8.1 Hz, 1H), 7.4–7.33 (m, 2H), 7.24–7.2 (m, 1H), 2.5 (q, J = 1.9 Hz, 3H). ^{13}C {^{1}H} NMR (101 MHz, $CDCl_3$) δ 135.3, 128.2, 124.9, 122.3 (q, J = 268.5 Hz) 121.7 (q, J = 36.7 Hz), 120.5, 120.2, 114.2 (q, J = 3.0 Hz), 111.7, 8.4. ^{19}F NMR (376 MHz, $CDCl_3$) δ –59.14. HRMS (EI, m/z) Calcd for $C_{10}H_8F_3N$ $[M]^+$: 199.0603; Found: 199.0601. The above analytical data agreed with previously published data.[8]

3-(trifluoromethyl)pyridin-2(1H)-one (**3m**): Prepared from pyridin-2(1*H*)-one following the General procedure. Column chromatography (EtOAc) afforded pure product as a white solid (44 mg, 68%). ^{1}H NMR (400 MHz, $CDCl_3$) δ 13.36 (brs, 1H), 7.89–7.85 (m, 1H), 7.67–7.63 (m, 1H), 6.39 (t, J = 6.8 Hz, 1H). ^{13}C {^{1}H} NMR (101 MHz, $CDCl_3$) δ 161.5, 140.8 (q, J = 5.0 Hz), 139.4, 122.8 (q, J = 271.4 Hz), 120.4 (q, J = 31.1 Hz), 105.7 ^{19}F NMR (376 MHz, $CDCl_3$) δ –66.22 (s). HRMS (EI, m/z) Calcd for $C_6H_4F_3NO$ $[M]^+$: 163.0239; Found: 163.0235. The above analytical data agreed with previously published data. [25]

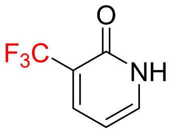

6-chloro-4-(trifluoromethyl)pyridazin-3(2H)-one (**3n**): Prepared from 6-chloropyridazin-3(2*H*)-one following the General procedure. Column chromatography (Et_2O) afforded pure product as a white solid (62 mg, 78%). ^{1}H NMR (400 MHz, $CDCl_3$) δ 7.6 (q, J = 1.0 Hz). ^{13}C {^{1}H} NMR (101 MHz, $CDCl_3$) δ 157.1, 138.5, 133.6 (q, J = 5.0 Hz), 131.6 (q, J = 33.7 Hz), 120.3 (q, J = 273.8 Hz). ^{19}F NMR (376 MHz, $CDCl_3$) δ –68.13 (s). HRMS (EI, m/z) Calcd for $C_5H_2ON_2ClF_3Na$ $[M+Na]^+$: 220.97000; Found: 220.97003

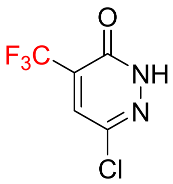

(S)-2-(4-isobutyl-3-(trifluoromethyl)phenyl)propanoic acid (**3o**): Prepared from (S)-2-(4-isobutylphenyl)propanoic acid following the General procedure. Crude product 35% ^{19}F NMR yield. ^{19}F NMR (376 MHz, $CDCl_3$) δ –59.24 (s). The above analytical data agreed with previously published data.[26]

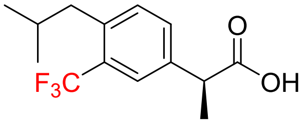

1,1',3,3',5,5'-Hexamethyl-4,4'-bis(trifluoromethyl)-[1,1'-bi(cyclohexane)]-2,2',5,5'-tetraene (**3p**): Prepared from mesitylene following the *General procedure*. Column chromatography (pentane) afforded pure product as a white solid (97 mg, 64%). ^{1}H NMR (400 MHz, $CDCl_3$) δ 5.77 (s, 4H), 3.1 (q, J = 8.4 Hz, 2H), 1.87 (s, 12H), 1.03 (s, 6H). ^{13}C {^{1}H} NMR (101 MHz, $CDCl_3$) δ 133.9, 126.4, 126.3 (q,

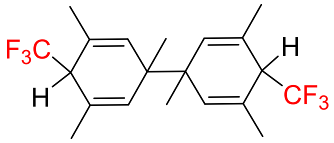

$J = 284.3$ Hz), 49.4 (q, $J = 26.0$ Hz), 43.5, 23.2 (q, $J = 2.5$ Hz), 22.2. ^{19}F NMR (376 MHz, $CDCl_3$) δ –64.76 (d, $J = 8.3$ Hz).

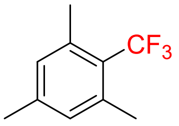

1,3,5-Trimethyl-2-(trifluoromethyl)benzene (**3q**): Prepared from mesitylene following the *General procedure.* Column chromatography (pentane) afforded pure product as a colorless liquid (21 mg, 28%). ^{1}H NMR (400 MHz, $CDCl_3$) δ 6.9 (s, 2H), 2.45 (q, $J = 3.4$ Hz, 6H), 2.3 (s, 3H). ^{13}C {^{1}H} NMR (101 MHz, $CDCl_3$) δ 141.0, 137.4 (q, $J = 2.1$ Hz), 131.0, 126.3 (q, $J = 275.8$ Hz), 124.9 (q, $J = 28.6$ Hz), 21.5 (q, $J = 4.0$ Hz), 21.0. ^{19}F NMR (376 MHz, $CDCl_3$) δ –54.24 (s). HRMS (EI, m/z) Calcd for $C_{10}H_{11}F_3$ $[M]^+$: 188.0807; Found: 188.0807. The above analytical data agreed with previously published data.[5]

3-(Trifluoromethyl)-1,2-dihydro-4H-pyrazolo[3,4-d]pyrimidin-4-one (**4**): Prepared from allopurinol following the *General procedure*. Column chromatography (EtOAc) afforded pure product as a white solid (48 mg, 59%). ^{1}H NMR (400 MHz, MeOD) δ 8.08 (s). ^{13}C {^{1}H} NMR (101 MHz, MeOD) δ 158.0, 156.5, 150.1, 138.9 (q, $J = 40.3$ Hz), 121.9 (q, $J = 268.4$ Hz), 103.9. ^{19}F NMR (376 MHz, MeOD) δ –62.06. HRMS (ESI, m/z) Calcd for $C_6H_3ON_4F_3Na$ $[M+Na]^+$: 227.01512; Found: 227.01525. The above analytical data agreed with previously published data.[9]

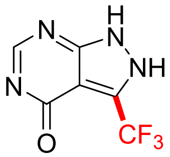

1,3,7-Trimethyl-8-(trifluoromethyl)-3,7-dihydro-1H-purine-2,6-dione (**5**): Prepared from caffeine following the *General procedure.* Column chromatography (cyclohexane/EtOAc, 2:1) afforded pure product as a white solid (61 mg, 58%). ^{1}H NMR (400 MHz, $CDCl_3$) δ 4.15 (q, $J = 1.3$ Hz, 3H), 3.58 (s, 3H), 3.41 (s, 3H). ^{13}C {^{1}H} NMR (101 MHz, $CDCl_3$) δ 155.6, 151.5, 146.6, 139.0 (q, $J = 40.1$ Hz), 118.3 (q, $J = 271.3$ Hz), 109.8, 33.3 (q, $J = 2.0$ Hz), 30.0, 28.3. ^{19}F NMR (376 MHz, $CDCl_3$) δ –62.89 (s). HRMS (ESI, m/z) Calcd for $C_9H_{10}O_2N_4F_3$ $[M+H]^+$: 263.07504; Found: 263.07530. The above analytical data agreed with previously published data.[5]

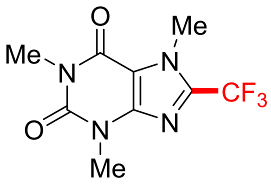

3-Methyl-7-propyl-8-(trifluoromethyl)-3,7-dihydro-1H-purine-2,6-dione (**6**): Prepared from 3-methyl-7-propyl-3,7-dihydro-1*H*-purine-2,6-dione following the *General procedure*. Reverse phase column chromatography (C18-silica gel, MeCN/H_2O) afforded product as a white solid (49 mg, 44%). ^{1}H NMR (400

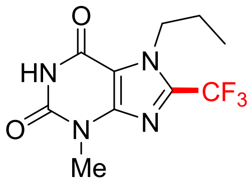

MHz, $CDCl_3$) δ 9.67 (br, 1H), 4.41–4.37 (m, 2H), 3.55 (s, 3H) 1.9 (sx, *J* = 7.5 Hz, 2H), 0.98 (t, *J* = 7.4 Hz, 3H). ^{13}C {^1H} NMR (101 MHz, $CDCl_3$) δ 154.8, 151.3, 148.7, 139.1 (q, *J* = 40.0 Hz), 118.3 (q, *J* = 271.4 Hz), 109.9, 49.0, 29.3, 24.7, 10.8. ^{19}F NMR (376 MHz, $CDCl_3$) δ –62.02. HRMS (EI, m/z) Calcd for $C_{10}H_{11}F_3N_4O_2$ $[M]^+$: 276.0829; Found: 276.0833.

Ethyl (S)-2-acetamido-3-(2-(trifluoromethyl)-1H-indol-3-yl)propanoate (**7**): Prepared from ethyl

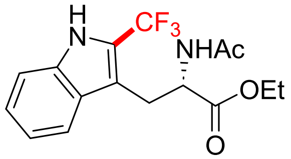

acetyl-*L*-tryptophanate following the *General procedure*. Reverse phase column chromatography (C18-silica gel, MeCN/H_2O) afforded product as a white solid (88 mg, 64%). ^1H NMR (400 MHz, $CDCl_3$) δ 9.41 (s, 1H), 7.7 (d, *J* = 8.1 Hz, 1H), 7.33–7.24 (m, 2H), 7.15 (ddd, *J* = 8.1, 6.8, 1.2 Hz, 1H), 6.2 (d, *J* = 8.2 Hz, 1H), 4.18–4.01 (m, 2H), 7.15 (dd, *J* = 14.9, 1.1 Hz, 1H), 4.99–4.93 (m, 1H), 4.41–4.37 (m, 2H), 3.4 (ddq, *J* = 14.1, 6.5, 1.3 Hz, 1H), 3.4 (ddq, *J* = 43.1, 6.5, 1.2 Hz, 1H), 1.16 (t, *J* = 7.1 Hz, 3H). ^{13}C {^1H} NMR (101 MHz, $CDCl_3$) δ 171.9, 170.2, 135.6, 127.4, 124.9, 122.8 (q, *J* = 36.6 Hz), 122.1 (q, *J* = 269.0 Hz), 120.8, 120.0, 112.2, 112.0 (q, *J* = 2.7 Hz), 61.8, 52.8, 27.1, 23.1, 13.9. ^{19}F NMR (376 MHz, $CDCl_3$) δ –58.27 (s). HRMS (ESI, m/z) Calcd for $C_{16}H_{17}O_3N_2F_3Na$ $[M+Na]^+$: 365.10835; Found: 365.10847. The above analytical data agreed with previously published data.[9]

2-(Acetoxymethyl)-5-(6-oxo-8-(trifluoromethyl)-1,6-dihydro-9H-purin-9-yl)tetrahydrofuran-3,4-diyl diacetate (**8**): Prepared from inosine triacetate following the

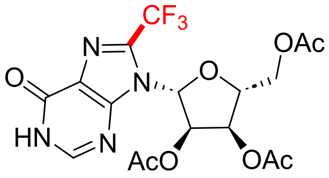

General procedure. Column chromatography (EtOAc) afforded pure product as a white solid (79 mg, 43%). ^1H NMR (400 MHz, $(CD_3)_2CO$) δ 11.66 (s, 1H), 8.36 (s, 1H), 6.28 (dd, *J* = 6.3, 4.5 Hz, 1H), 6.15 (d, *J* = 4.5 Hz, 1H), 5.82–5.79 (m, 1H), 4.51–4.45 (m, 2H), 4.35–4.27 (m, 1H), 2.12 (s, 3H), 2.07 (s, 3H), 2.00 (s, 3H). ^{13}C {^1H} NMR (101 MHz, $(CD_3)_2CO$) δ 205.4, 169.8, 169.3, 169.2, 155.8, 149.8, 148.1, 136.5 (q, *J* = 40.2 Hz), 124.5, 118.6 (q, *J* = 270.4 Hz), 80.5, 72.3, 70.0, 62.5, 19.7, 19.6, 19.4. ^{19}F NMR (376 MHz, $(CD_3)_2CO$) δ –60.77 (s). HRMS (ESI, m/z) Calcd for $C_{17}H_{17}O_8N_4F_3Na$ $[M+Na]^+$: 485.08907; Found: 485.08928. The ^1H NMR and ^{19}F NMR data agree with previously published data.[10]

5-(Trifluoromethyl)pyrimidine-2,4(1H,3H)-dione (**9**): Prepared from uracil following the *General*

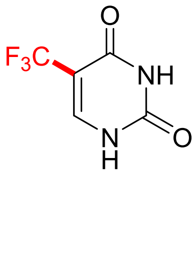

procedure. Column chromatography (hexane/EtOAc, 2:1 to 1:1) afforded pure product as a white solid (51 mg, 71%). ^1H NMR (400 MHz, DMSO-d_6) δ 11.59

(brs, 2H), 8.02 (s, 1H). ^{13}C {^{1}H} NMR (101 MHz, DMSO-d_6) δ 160.0, 150.7, 144.3 (q, J = 5.6 Hz), 123.0 (q, J = 268.8 Hz), 101.8 (q, J = 31.8 Hz). ^{19}F NMR (376 MHz, DMSO-d_6) δ –61.63 (s). HRMS (EI, m/z) Calcd for $C_5H_3F_3N_2O_2$ $[M]^+$: 180.0141; Found: 180.0137. The above analytical data agreed with previously published data.[11]

1,3-Dimethyl-5-(trifluoromethyl)pyrimidine-2,4(1H,3H)-dione (**10**): Prepared from 1,3-dimethyluracil following the *General procedure*. Column chromatography (hexane/EtOAc, 5:1) afforded pure product as a white solid (20 mg, 48%). ^{1}H NMR (300 MHz, $CDCl_3$) δ 7.68 (q, J = 1.2 Hz, 1H), 3.48 (s, 3H), 3.35 (s, 3H). ^{13}C {^{1}H} NMR (75 MHz, $CDCl_3$) δ 158.8, 151.1, 143.6 (q, J = 5.9 Hz), 122.1 (q, J = 269.8 Hz), 104.3 (q, J = 33.0 Hz), 37.9, 28.2. ^{19}F NMR (282 MHz, $CDCl_3$) δ –63.8 (d, J = 1.0 Hz). HRMS (ESI, m/z) Calcd for $C_7H_8F_3N_2O_2$ $[M+H]^+$: 209.0532; Found: 209.0533. The above analytical data agreed with previously published data.[11]

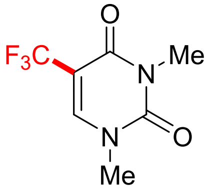

6-Methyl-5-(trifluoromethyl)pyrimidine-2,4(1H,3H)-dione (**11**): Prepared from pseudothymine following the *General procedure*. Column chromatography (DCM/EtOAc, 1:1) afforded pure product as a white solid (30 mg, 76%). ^{1}H NMR (300 MHz, CD_3OD) δ 2.34 (q, J = 2.7 Hz). ^{13}C {^{1}H} NMR (75 MHz, CD_3OD) δ 162.6, 157.9 (q, J = 1.9 Hz), 151.7, 125.2 (q, J = 270.2 Hz), 102.4 (q, J = 31.3 Hz), 18.0 (q, J = 4.1 Hz). ^{19}F NMR (282 MHz, CD_3OD) δ –57.5 (q, J = 2.6 Hz). HRMS (ESI, m/z) Calcd for $C_6H_5F_3N_2O_2Na$ $[M+Na]^+$: 217.0195; Found: 217.0190. The above analytical data agreed with previously published data.[11]

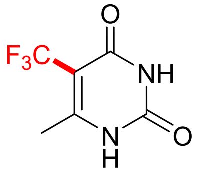

6-(Trifluoromethyl)-1,2,4-triazine-3,5(2H,4H)-dione (**12**): Prepared from azauracil following the *General procedure*. Column chromatography (DCM/EtOAc 2:1) afforded pure product as a white solid (71 mg, 98%). ^{1}H NMR (300 MHz, CD_3CN) δ 2.54 (s). ^{13}C {^{1}H} NMR (75 MHz, CD_3CN) δ 154.2, 149.1, 133.3 (q, J = 32.1 Hz), 120.8 (q, J = 272.2 Hz). ^{19}F NMR (282 MHz, CD_3CN) δ –68.5 (s). HRMS (ESI, m/z) Calcd for $C_4HF_3N_3O_2$ $[M+H]^-$: 180.0026; Found: 180.0029. The above analytical data agreed with previously published data.[12]

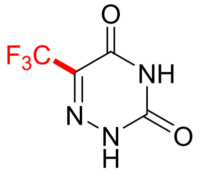

(2R,3R,4R,5R)-2-(acetoxymethyl)-5-(2,4-dioxo-5-(trifluoromethyl)-3,4-dihydropyrimidin-1(2H)-yl)tetrahydrofuran-3,4-diyl diacetate (**13**): Prepared from uridine

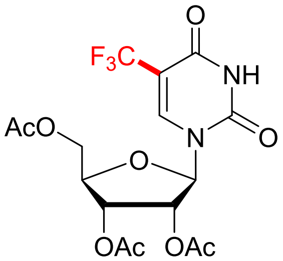

triacetate following the *General procedure*. Column chromatography (hexane/EtOAc, 1:2) afforded pure product as a colorless oil (57 mg, 65%), which solidified upon storage. ^{1}H NMR (300 MHz, $CDCl_3$) δ 9.35 (br s, 1H), 8.00 (q, J = 1.2 Hz, 1H), 6.06 (d, J = 4.9 Hz, 1H), 5.37–5.29 (m, 2H), 4.45–4.28 (m, 3H), 2.13 (m, 3H), 2.12 (m, 3H), 2.10 (m, 3H). ^{13}C {^{1}H} NMR (75 MHz, $CDCl_3$) δ 170.4, 169.9, 169.8, 158.3, 149.5, 140.3 (q, J = 6.2 Hz), 121.75 (q, J = 270.3 Hz), 106.2 (q, J = 33.2 Hz), 87.9, 80.6, 73.3, 70.0, 62.9, 20.5 (2Me), 20.4. ^{19}F NMR (282 MHz, $CDCl_3$) δ –63.5 (s). HRMS (ESI, m/z) Calcd for $C_{16}H_{16}F_3N_2O_9$ $[M+H]^-$: 437.0813; Found: 437.0870. The above analytical data agreed with previously published data.[13]

1-((2R,3R,4S,5R)-3,4-dihydroxy-5-(hydroxymethyl)tetrahydrofuran-2-yl)-5-(trifluoromethyl)pyrimidine-2,4(1H,3H)-dione (**14**): Prepared from uridine following the *General procedure*. Column chromatography (DCM/EtOAc, 1:1 to 1:2) afforded pure product as a white amorphous solid (36 mg, 29%). ^{1}H NMR (300 MHz, CD_3OD) δ 8.91 (q, J = 1.2 Hz, 1H), 5.89 (d, J = 2.7 Hz, 1H), 4.21–4.17 (m, 2H), 4.08–4.04 (m, 1H), 3.92 (dd, J = 12.1, 2.5 Hz, 1H), 3.76 (dd, J = 12.1, 2.2 Hz, 1H). ^{13}C {^{1}H} NMR (75 MHz, CD_3OD) δ 161.2, 151.5, 143.9 (q, J = 5.9 Hz), 123.9 (q, J = 269.1 Hz), 105.4 (q, J = 33.0 Hz), 91.5, 86.1, 76.4, 70.5, 61.2. ^{19}F NMR (282 MHz, CD_3OD) δ –64.4 (s). HRMS (ESI, m/z) Calcd for $C_{10}H_{11}F_3N_2O_6Na$ $[M+Na]^+$: 335.0461; Found: 335.0470. The above analytical data agreed with previously published data.[13]

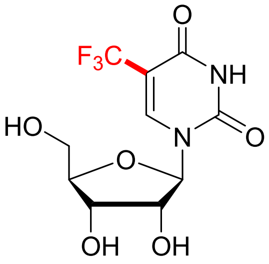

Trifluridine (**15**): Prepared from deoxyuridine following the *General procedure*. Column chromatography (DCM/EtOAc, 1:1) afforded pure product as a colorless oil (57 mg, 65%), which solidified upon storage. ^{1}H NMR (400 MHz, DMSO-d_6) δ 11.83 (brs, 1H), 8.73 (s, 1H), 6.08 (t, J = 6.0 Hz, 1H), 5.27 (d, J = 4.4 Hz, 1H), 5.21 (t, J = 4.5 Hz, 1H), 4.25 (p, J = 4.8 Hz, 1H), 3.83 (q, J = 3.1 Hz, 1H), 3.68 – 3.56 (m, 2H), 2.20 (t, J = 5.7 Hz, 2H). ^{13}C {^{1}H} NMR (101 MHz, DMSO-d_6) δ 159.1, 149.6, 142.3 (q, J = 6.3 Hz), 122.8 (q, J = 269.1 Hz), 102.7 (q, J = 31.9 Hz), 87.7, 85.5, 69.4, 60.3, 40.7. ^{19}F NMR (376 MHz, DMSO-d_6) δ –61.60 (s). HRMS (ESI, m/z) Calcd for $C_{10}H_{11}O_5N_2F_3Na$ $[M+Na]^+$: 319.05123; Found: 319.05134. The above analytical data agreed with previously published data.[13]

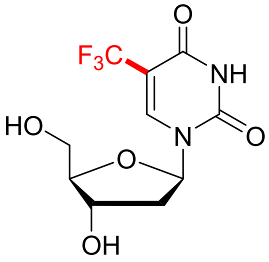

(3S,4aR,4a1R,5aS,8aR,8a1S,15aS)-10,11-dimethoxy-3-(trifluoromethyl)-2,3,4a1,5,5a,7,8,8a1,15,15a-decahydro-4aH,14H-4,6-(metheno)indolo[3,2,1-ij]oxepino[2,3,4-de]pyrrolo[2,3-h]quinolin-14-one (**16**): Prepared from brucine following the *General procedure*. Reverse phase column chromatography (C18-silica gel, MeCN/H_2O) afforded product as a white solid (85 mg, 46%).

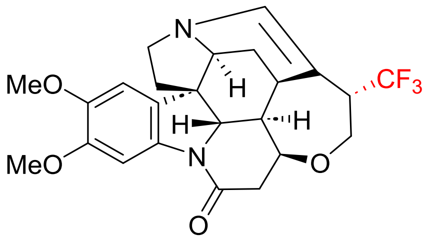

^{1}H NMR (400 MHz, C_6D_6) δ 8.27 (s, 1H), 6.56 (s, 1H), 5.54 (s, 1H), 4.02 (dd, J = 12.7, 6.7 Hz, 1H), 3.57–3.47 (m, 7H), 3.35–3.19 (m, 3H), 3.04–2.93 (m, 2H), 2.84–2.72 (m, 1H), 2.68–2.61 (m, 1H), 2.55 (dd, J = 16.2, 4.5 Hz, 1H), 2.18 (br s, 1H), 2.1 (dt, J = 13.3, 3.6 Hz, 1H), 1.9–1.82 (m, 1H), 1.59–1.52 (m, 1H), 1.43 (dt, J = 13.2, 2.6 Hz, 1H), 0.85–0.8 (m, 1H). ^{13}C {^{1}H} NMR (101 MHz, (C_6D_6) δ 169.5, 151.0, 147.5, 141.7, 136.9, 127.0 (q, J = 281.0 Hz), 124.4, 107.5, 104.9 (q, J = 1.9 Hz), 102.0, 78.4, 66.1 (q, J = 2.8 Hz), 64.7, 58.7, 57.0, 55.8, 54.0, 53.7, 51.6, 48.9 (q, J = 24.4 Hz), 45.6, 41.4, 27.2, 27.0. ^{19}F NMR (376 MHz, C_6D_6) δ –69.8 (d, J = 9.6 Hz). HRMS (ESI, m/z) Calcd for $C_{24}H_{25}O_4N_2F_3Na$ $[M+Na]^+$: 485.16586; Found: 485.16601. The above analytical data agreed with previously published data.[14]

Isolation of ammonium copper(II) sulfate hexahydrate

An oven-dried Schlenk flask was charged with tetrabutylammonium tetrakis(trifluoromethyl)cuprate(III) (291 mg, 0.5 mmol, 0.25 equiv.) and $(NH_4)_2S_2O_8$ (457 mg, 2 mmol, 1 equiv.) then it was evacuated and backfilled with nitrogen (3×). Dry MeCN (5 ml) was added under inert atmosphere. LED light (λ_{max} = 387 nm) was introduced into the flask via a glass rod and after sealing under a nitrogen atmosphere the solution was irradiated for 16 h at room temperature. The reaction was followed by ^{19}F NMR until all tetrakis(trifluoromethyl)cuprate(III) was consumed. MeCN was removed from the reaction mixture using syringe filter and the remaining solid in the Schlenk flask was treated with MeOH and sonicated. Blue precipitate formed, which was filtered using a frit. Crystallization of the blue precipitate from water led to the formation of ammonium copper(II) sulfate hexahydrate (110 mg).

UV-Vis and fluorescence spectroscopy

Absorption spectra and molar absorption coefficients were measured on an Agilent Cary 8454 UV-vis spectrophotometer with 1 cm quartz cuvettes.

A stock solution of **1** (200 mM in DMSO) was serially diluted to obtain concentrations of 100 mM, 10 mM, 1 mM, and 0.1 mM. For each dilution, UV-Vis absorption spectra were recorded in a 1 cm path length quartz cuvette at room temperature (~22 °C). The aim of the experiment was to assess whether **1** exhibits absorption around the irradiation wavelength (385 nm), as no significant absorption was observed at lower concentrations. The results revealed that **1** displays a measurable absorption around 385 nm only at higher concentrations (≥1 mM).

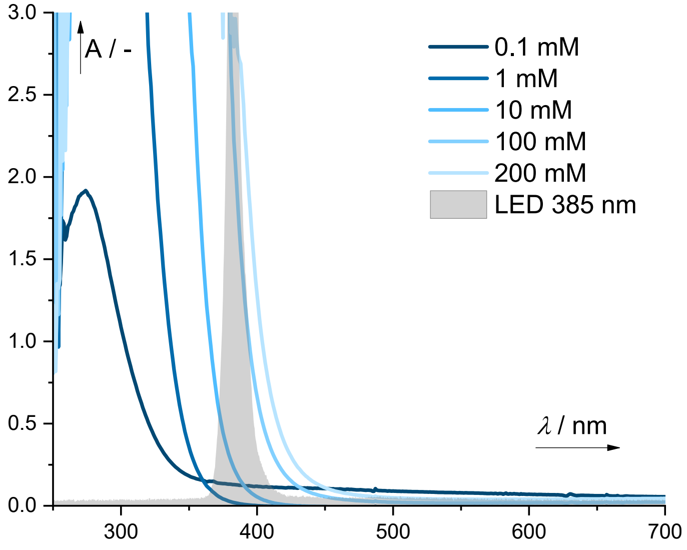

Figure S3. UV-Vis absorption spectra of **1** in MeCN at concentrations 0.1–200 mM).

A solution of **1** (10 μM), 1,3,5-trimethoxybenzene (50 μM), and $(NH_4)_2S_2O_8$ (50 μM) in MeCN (3 ml) was prepared in a 1 cm path length quartz cuvette and irradiated at 272 nm using a Duetta Horiba spectrometer with 5 nm slits. The experiment was conducted at room temperature (~22 °C). UV-Vis absorption spectra were recorded at the following time points: 0 min (prior to irradiation), 2 min, 10 min, 20 min, and 30 min.

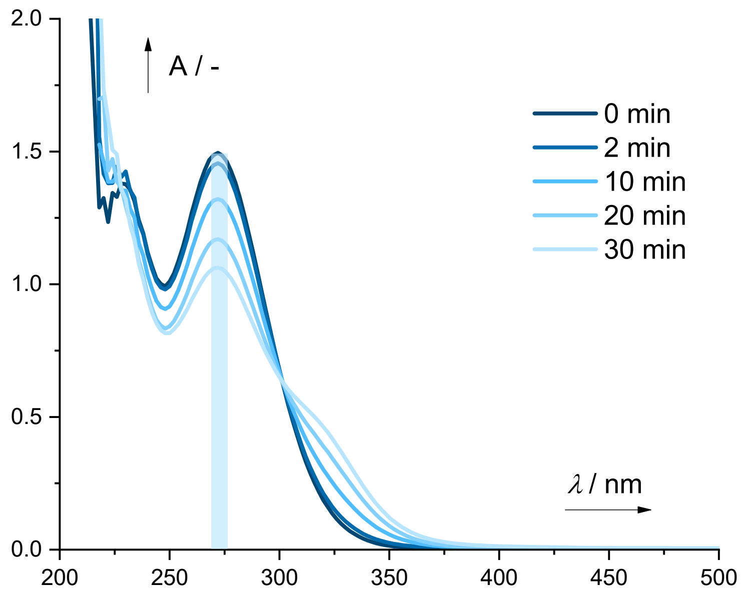

Figure S4. UV-vis absorption spectra of **1** (c = 10 µM), 1,3,5-trimethoxybenzene (c = 50 µM) and $(NH_4)_2S_2O_8$ (c = 50 µM), irradiated with 272 nm light in MeCN.

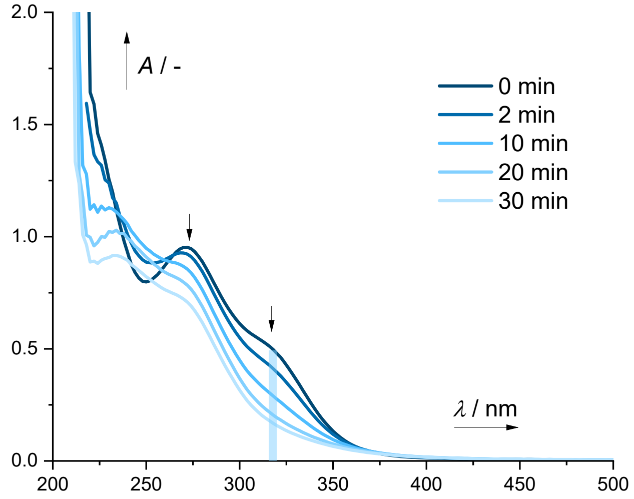

Figure S5. UV-vis absorption spectra of **1** (c = 10 µM), 1,3,5-trimethoxybenzene (c = 50 µM) and $(NH_4)_2S_2O_8$ (c =50 µM), irradiated with 315 nm LED light in MeCN.

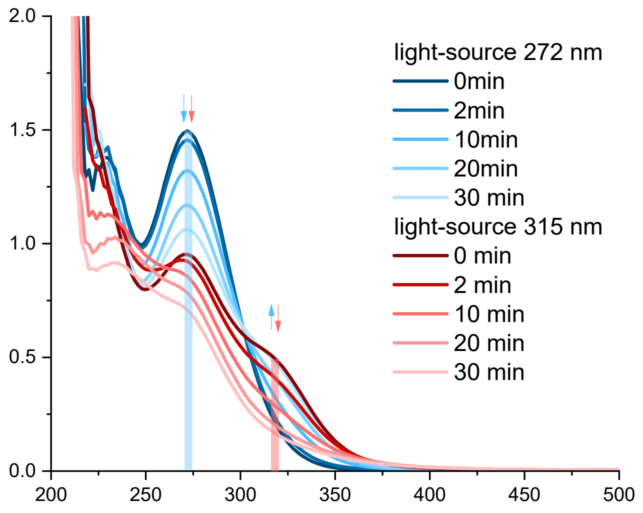

Figure S6. UV-Vis absorption spectra of a solution containing **1** (c = 10 µM), 1,3,5-trimethoxybenzene (c = 50 µM) and $(NH_4)_2S_2O_8$ (c =50 µM) in MeCN upon irradiation at 272 nm light and follow-up irradiation with 315 nm.

A solution of **1** (10 µM), 1,3,5-trimethoxybenzene (50 µM), and $(NH_4)_2S_2O_8$ (50 µM) in MeCN (3 ml) was prepared in a 1 cm path length quartz cuvette and irradiated at 385 nm using a Duetta Horiba spectrometer with 5 nm slits. The experiment was conducted at room temperature (~22 °C).

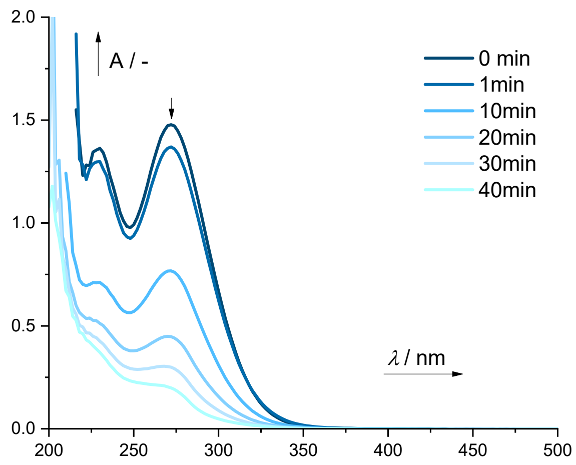

Figure S7. UV-Vis absorption spectra of a solution containing **1** (c = 10 µM), 1,3,5-trimethoxybenzene (c = 50 µM) and $(NH_4)_2S_2O_8$ (c = 50 µM mM) irradiated with 385 nm LED in MeCN.

A 100 mM solution of **1** in DMSO (3 ml) was prepared in a 1 cm path length quartz cuvette and irradiated with a 385 nm LED light source at room temperature (~22 °C). UV-Vis absorption spectra were recorded at 2-minute intervals over a total period of 12 minutes. The experiment aimed to demonstrate the photochemical behavior of **1** at higher concentration. Upon irradiation, a gradual increase in absorbance (550–1100 nm) was observed (characteristic of copper(II) complexes).[15]

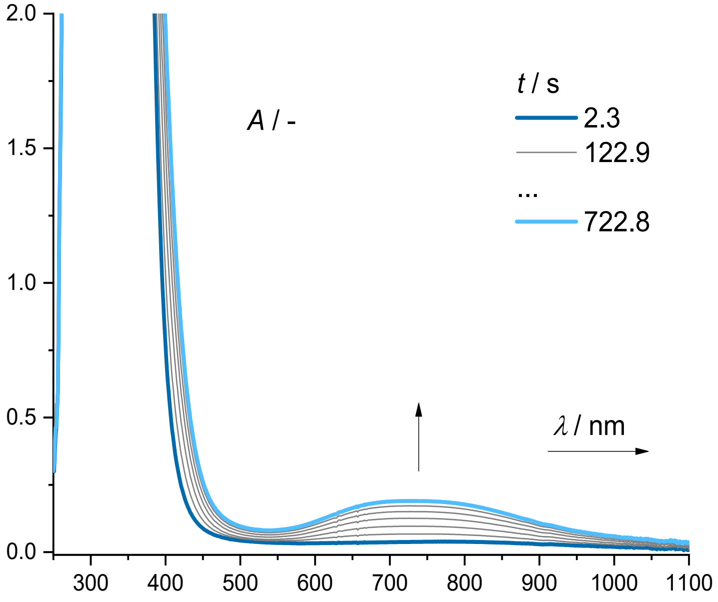

Figure S8. UV-Vis absorption spectra of a 100 mM solution of **1** in DMSO, measured every 2 minutes during 12 minutes of irradiation with a 385 nm LED light.

UV-Vis absorption spectra were recorded to investigate potential ground-state interactions (e.g., charge-transfer complex formation) between $Bu_4N[Cu(CF_3)_4]$ (Tetrakis), 1,3,5-trimethoxybenzene (TMB) and $(NH_4)_2S_2O_8$ (PS). All samples were prepared in MeCN (3.0 ml total volume) and measured in a 1 cm path length quartz cuvette at room temperature (~22 °C). The mixtures were composed such that the final concentrations of the individual components in each solution correspond to the following:

- **TMB:** TMB (0.05 mM)
- **TMB-PS:** TMB (0.05 mM), PS (0.05 mM)
- **Tetrakis:** Tetrakis (0.01 mM)

- **Tetrakis-PS:** Tetrakis (0.01 mM), PS (0.05 mM)
- **Tetrakis-PS-TMB:** Tetrakis (0.01 mM), PS (0.05 mM), TMB (0.05 mM)
- **Tetrakis-TMB:** Tetrakis (0.01 mM), TMB (0.05 mM)

A theoretical UV-Vis spectrum (**"combined"**) was generated by arithmetic summation of the individual spectra of TMB and Tetrakis, using their corresponding final concentrations. A perfect agreement between this calculated spectrum and the measured spectrum of the ternary mixture indicated that no new absorption features emerge upon mixing, suggesting the absence of ground-state electronic interactions or charge-transfer complex formation.

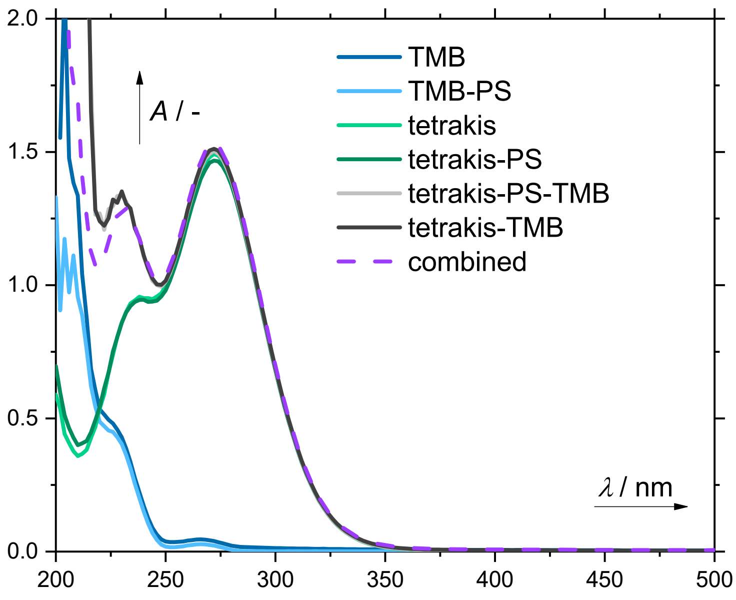

Figure S9. UV-Vis absorption spectra of ($Bu_4N[Cu(CF_3)_4]$ (tetrakis)), $(NH_4)_2S_2O_8$ (PS) and 1,3,5-trimethoxybenzene (TMB) in binary and ternary combinations in MeCN (3 ml). Final concentrations of components: 1,3,5-trimethoxybenzene (0.05 mM), $(NH_4)_2S_2O_8$ (0.05 mM), $Bu_4N[Cu(CF_3)_4]$ (0.01 mM).

Cyclic voltammetry

Cyclic voltammetry measurements were performed using Autolab PGSTAT204 potentiostat (Metrohm Autolab B.V.) under computer control (NOVA 2.1.5 software). A conventional three-electrode configuration consisted of glassy carbon or platinum working electrode (3 mm in diameter), platinum sheet counter electrode (1 cm^2) and Ag wire pseudo-reference electrode or standard calomel reference electrode. Glassy carbon working electrode was polished with 0.3 µm alumina on a polishing pad. Platinum working electrode was pre-treated by several cycles in blank. All CV measurements were performed in solution of 0.1 M Bu_4NPF_6 in MeCN (HPLC grade) at room temperature using the scan rate from 50 to 1000 mV/s. Prior to each experiment, the solutions were deoxygenated by bubbling with nitrogen, and nitrogen atmosphere was maintained throughout the course of the experiment. All potentials were referenced versus standard calomel electrode (ferrocene could not be used as internal standard, because it reacted with studied compound). CV of **1** is shown in Figure S10.

Spectroelectrochemical measurements were performed in OTTLE cell (Optically transparent thin-layer electro-chemical cell) with Pt mini-grid working and counter electrode and Ag wire pseudo-reference electrode. A potential sweep (5 mV/s) or potential pulse was applied to the cell and UV/Vis absorption spectra were recorded using an Agilent 8454 spectrometer.
For further details of OTTLE Cell, see:
https://research.reading.ac.uk/spectroelectrochemistry/optically-transparent-thin-layer-electrochemical-cells/room-temperature-ottle-cell/

Table S4. Redox potentials of **1** vs SCE (scan rate 250 mV/s).

Sample	E_{pc} (V)	E_{pa} (V)
Reduction 1	-1.43	-0.49
Reduction 2	-1.39; -2.10	-0.56; -0.01

E_{pc} = cathodic peak potential, E_{pa} = anodic peak potential
Measured on glassy carbon working electrode in 0.1 M Bu_4NPF_6 in MeCN

Redox potential of anodic waves depends on the switching potential and on the scan rate. For faster scan rates, 2 anodic waves were observed (Figure S11).

Spectroelectrochemistry revealed, that upon reduction a new species that absorb at 315 nm and can be assigned as Cu^{II} complex is formed. Further reduction gave species with absorbance at 227 nm, which would correspond to Cu^{I} complex (Figure S13). Initial Cu^{III} complex was not recovered during the return scan which indicates an irreversible change upon reduction. Cu^{II} complex was generated most efficiently by a pulse at –1.4 V. It would be stable upon reoxidation (Figure S14). On the other hand, after reduction to Cu^{I} complex and reoxidation to Cu^{II} complex, significant loss of absorbance at 315 nm was observed (Figure S13).

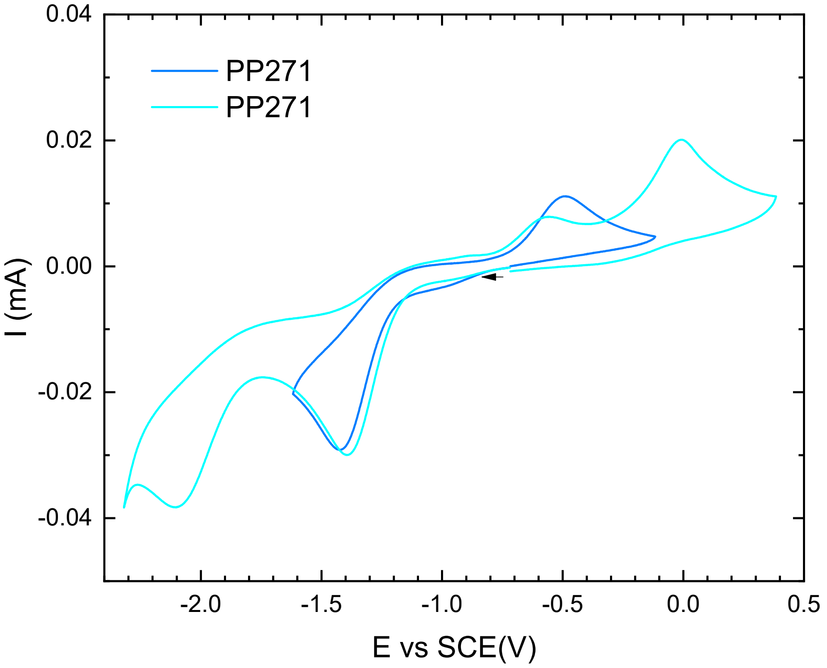

Figure S10. Cyclic voltammogram of **1** (c = 1 mM) in 0.1 M Bu_4NPF_6 in MeCN (glassy carbon working electrode, scan rate 250 mV/s).

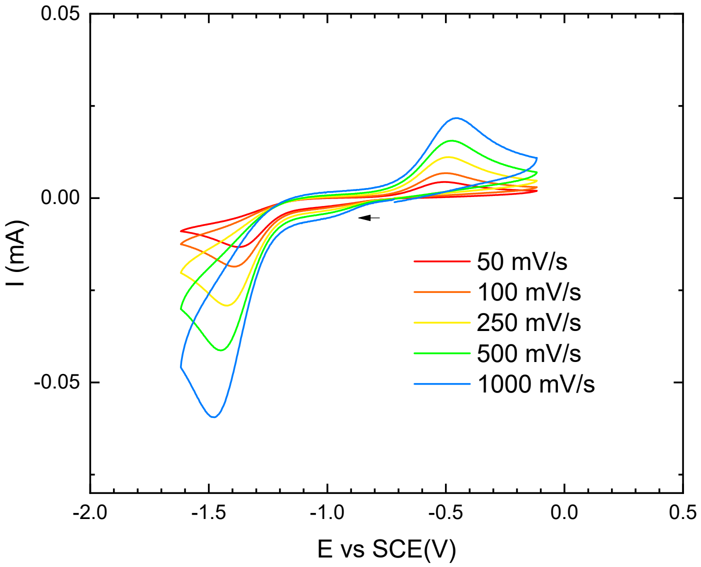

Figure S11. Cyclic voltammogram of **1** (c = 1 mM) in 0.1 M Bu_4NPF_6 in MeCN at different scan rates (glassy carbon working electrode).

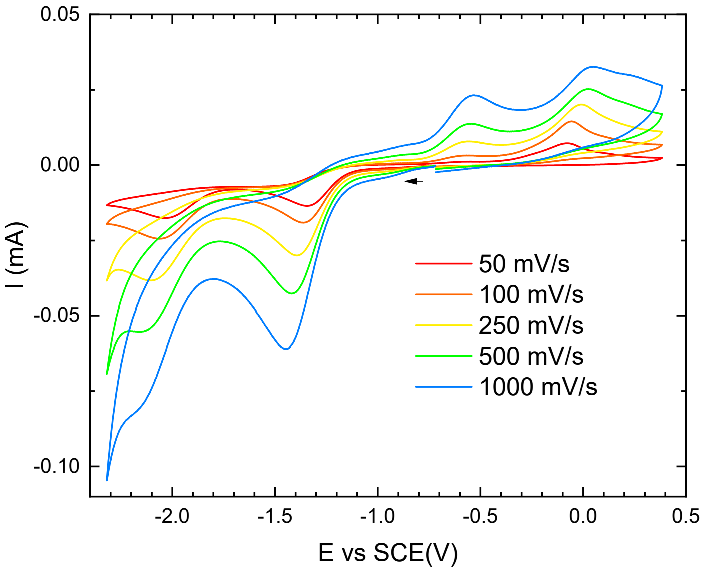

Figure S11. Cyclic voltammogram of **1** (c = 1 mM) in 0.1 M Bu_4NPF_6 in MeCN at different scan rates (glassy carbon working electrode).

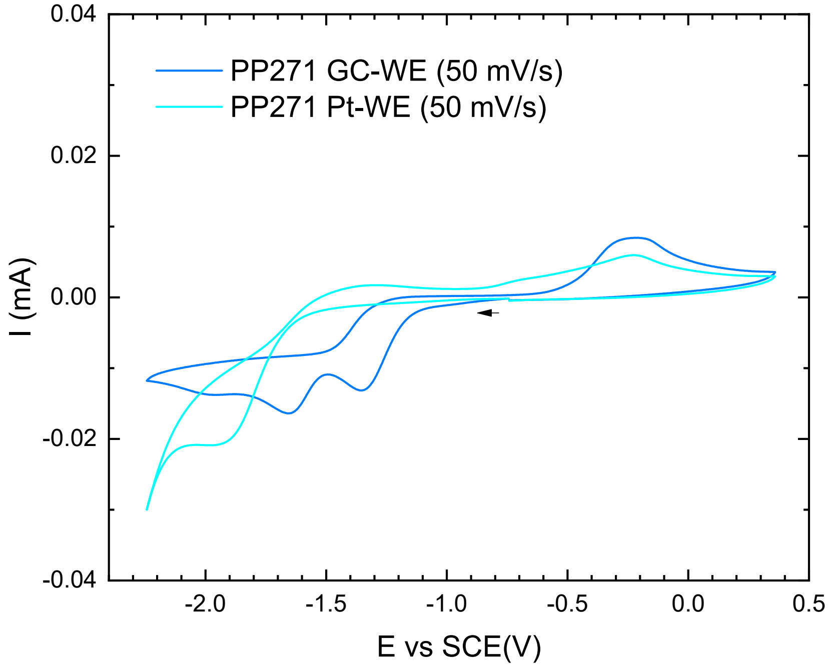

Figure S12. Cyclic voltammogram of **1** (*c* = 1 mM) in 0.1 M Bu_4NPF_6 in MeCN using different working electrode: glassy carbon and platinum (scan rate 50 mV/s).

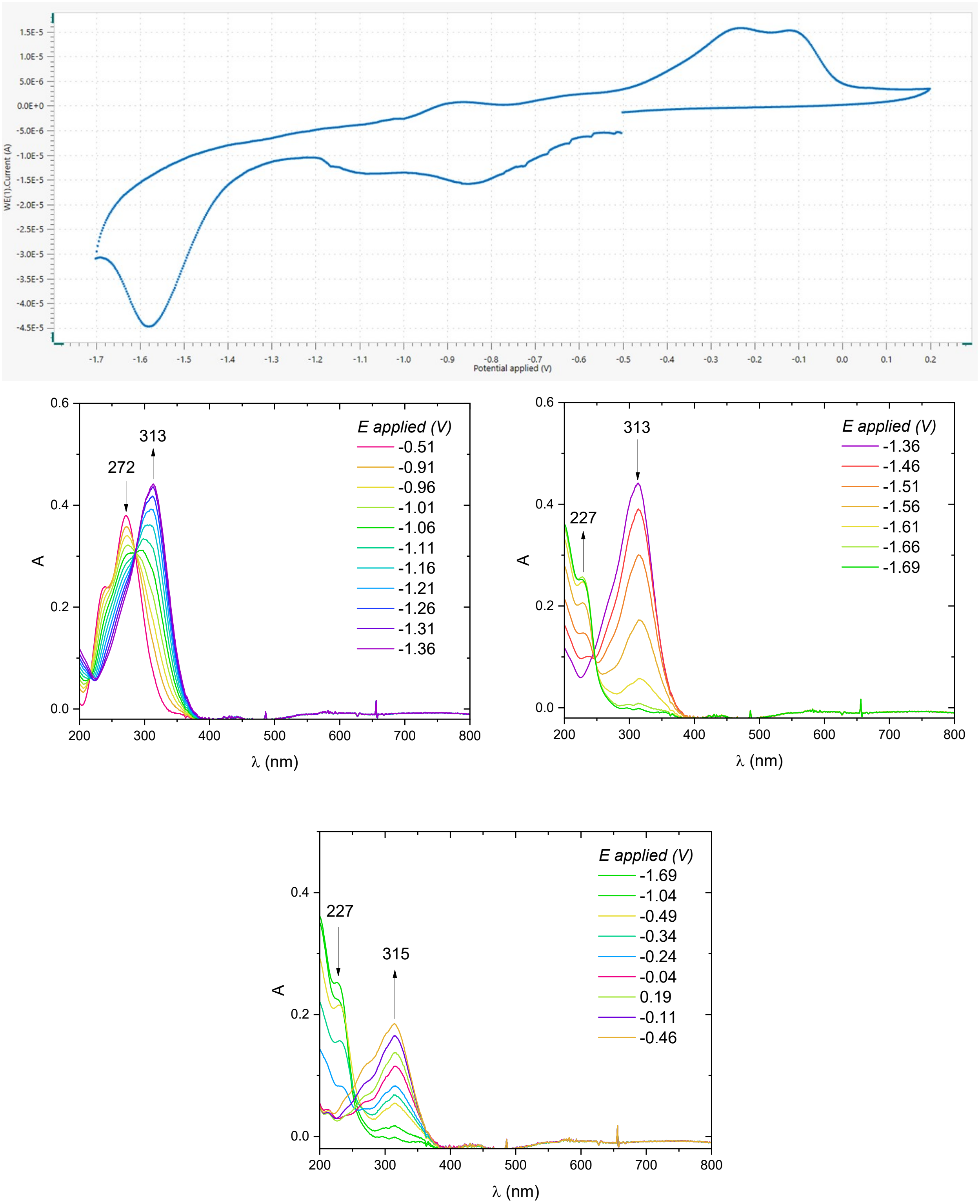

Figure S13. Spectroelectrochemistry – potential-dependent spectral changes of **1** during cyclic voltammetry in OTTLE.

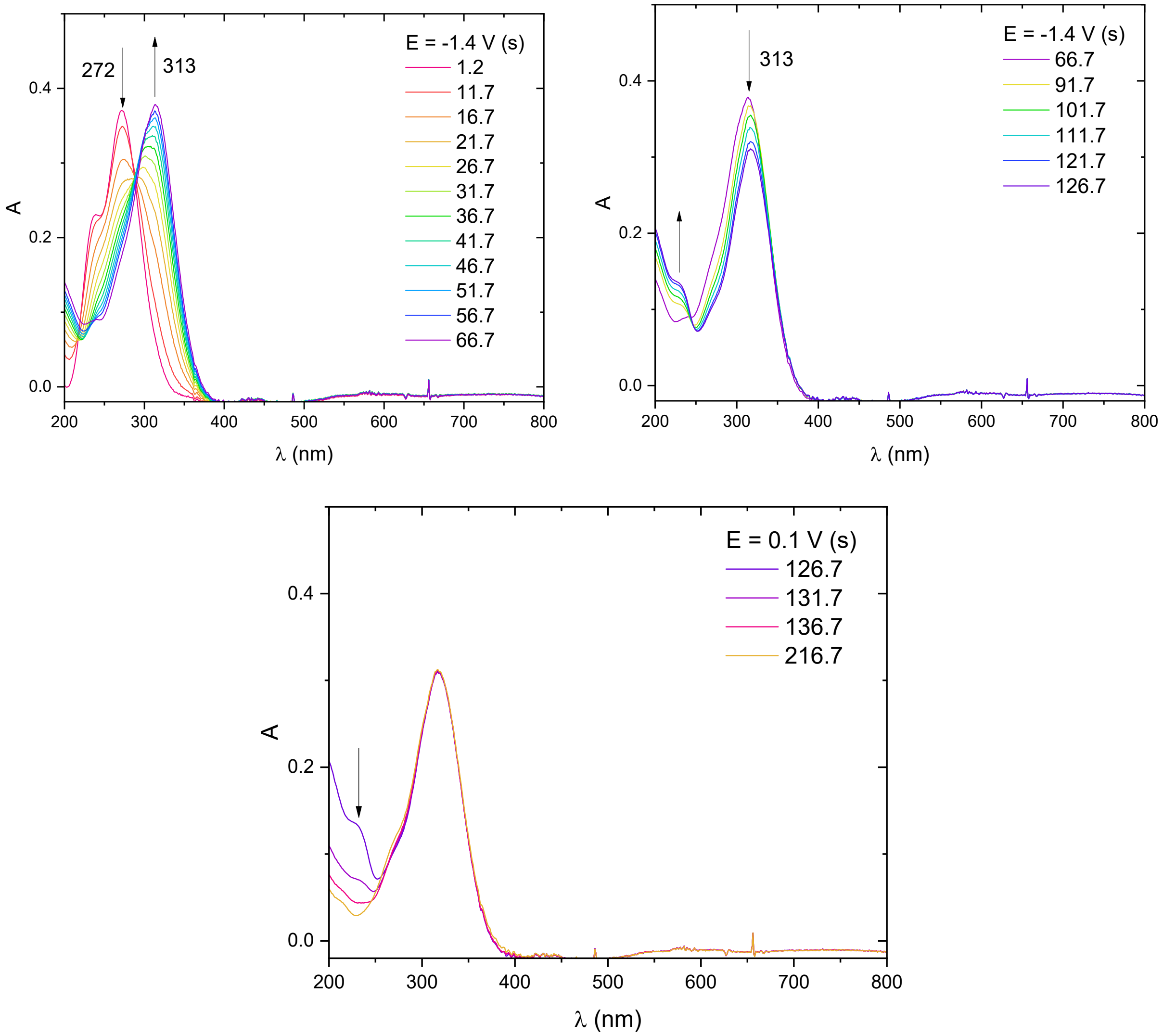

Figure S14. Spectroelectrochemistry of **1** – potential pulses applied: –1.4 V (time 1–127 s) followed by 0.1 V (time 127–217 s).

Photo-EPR studies

Experimental: All the EPR spectra were acquired on *Bruker* EMX^{plus} 10/12 CW (continuous wave) spectrometer equipped with a Premium-X-band microwave bridge and the standard rectangular probehead/cavity (ER160FCQ, *Bruker*). The g_{iso} value of radicals was determined using a built-in spectrometer frequency counter and an ER036TM NMR-Teslameter (*Bruker*). Typical uncertainty for the *g*-value determination reads 0.0002. Low-temperature experiments were performed directly in liquid N_2 (77 K). For this purpose, an acetonitrile solution was injected into the 3 mm (i.d.) EPR tube, which was inserted into "finger-dewar" (ER 167FDS, *Bruker*) pre-filled with the liquid nitrogen. Room-temperature spin trapping experiments were done by *in situ* irradiation (LED385 (λ_{max} = 387 nm, P = 5 W) of the reaction mixtures including the PBN (N-*tert*-Butyl-alpha-phenylnitrone in the total concentration of 10 mM) as a spin trapping agent. Common concentration of the $Bu_4N[Cu(CF_3)_4]$ for the spin trapping EPR experiment was 0.1 mM. The entire mixture in acetonitrile was injected into the special EPR flat cell (ER 165FCVT-Q, *Bruker*), in order to have sufficiently large irradiation area through the grid of the above-described rectangular cavity.

EPR calculations: Simulations were performed either by the above-described *Xenon* acquisition/processing software or by the standard Matlab *EasySpin* 6.0 toolbox.[16] Instrumental parameters such as microwave frequency, central field, sweep width and number of points were usually included in the simulation. In order to support the structure of F_3C-PBN radical adduct we ran density functional theory (DFT) calculations carried out by the *Gaussian 16* (A.03) quantum chemical package.[17] Geometry of PBN radical adducts were optimized by B3LYP/6-31+G(d,p) in the acetonitrile solvent environment using the universal solvation model based on solute electron density and on a continuum model.[18] The EPR hyperfine coupling constants (HFCCs, A) were calculated at the PBE0/EPR-II(H,F)/6-31G(d)(N) level. The C-PCM method[19,20] was used to model the mixture solvent (see above) environment.

Evaluation of the EPR study: First, EPR spectra were detected in the LED-off state before irradiation to exclude the presence of any transient/persistent radicals before the irradiative procedure (Figure S15), also EPR spectrum of PBN was measured before irradiation (Figure S16). Comparison of of Figures S17,S18 as well as S19 confirm that the primary PBN spin trapping adduct formed upon irradiation is formed by the reaction of PBN with $\bullet CF_3$ radical.

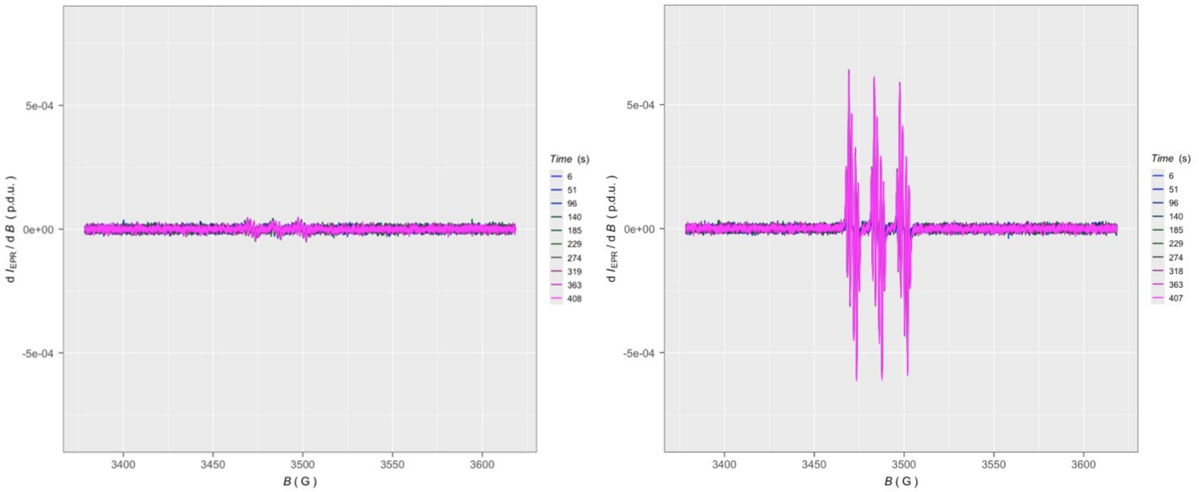

Figure S15. EPR spectra simultaneously recorded before and during continuous irradiation of the PBN with $Bu_4N[Cu(CF_3)_4]$ mixture corresponding to Figure S17, showing that only a negligible portion or radical adducts is formed before the irradiation.

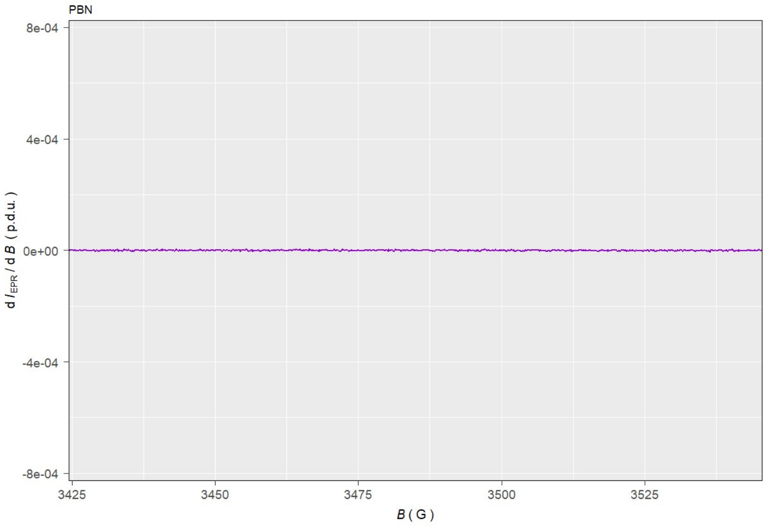

Figure S16. EPR spectrum of PBN (c = 10 mM) before irradiation in MeCN, indicating no formation of transient or persistent radical species.

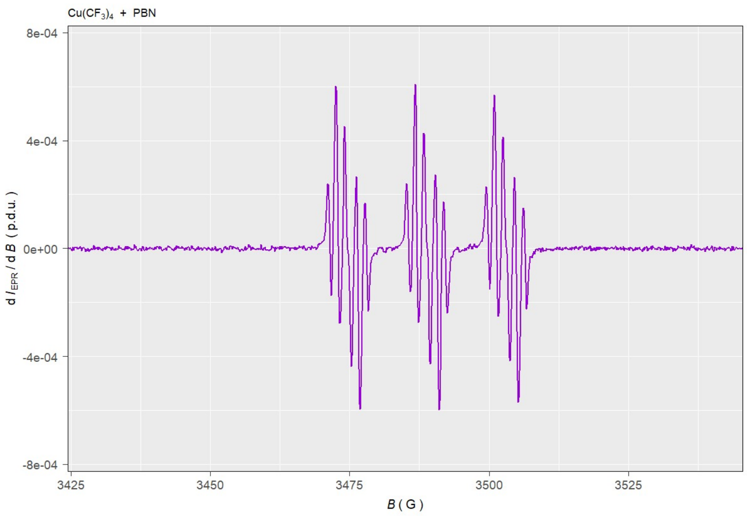

Figure S17. EPR spectrum of PBN-CF_3 radical adduct after irradiation of $Bu_4N[Cu(CF_3)_4]$ (c = 10 mM) with PBN (c = 10 mM) in MeCN.

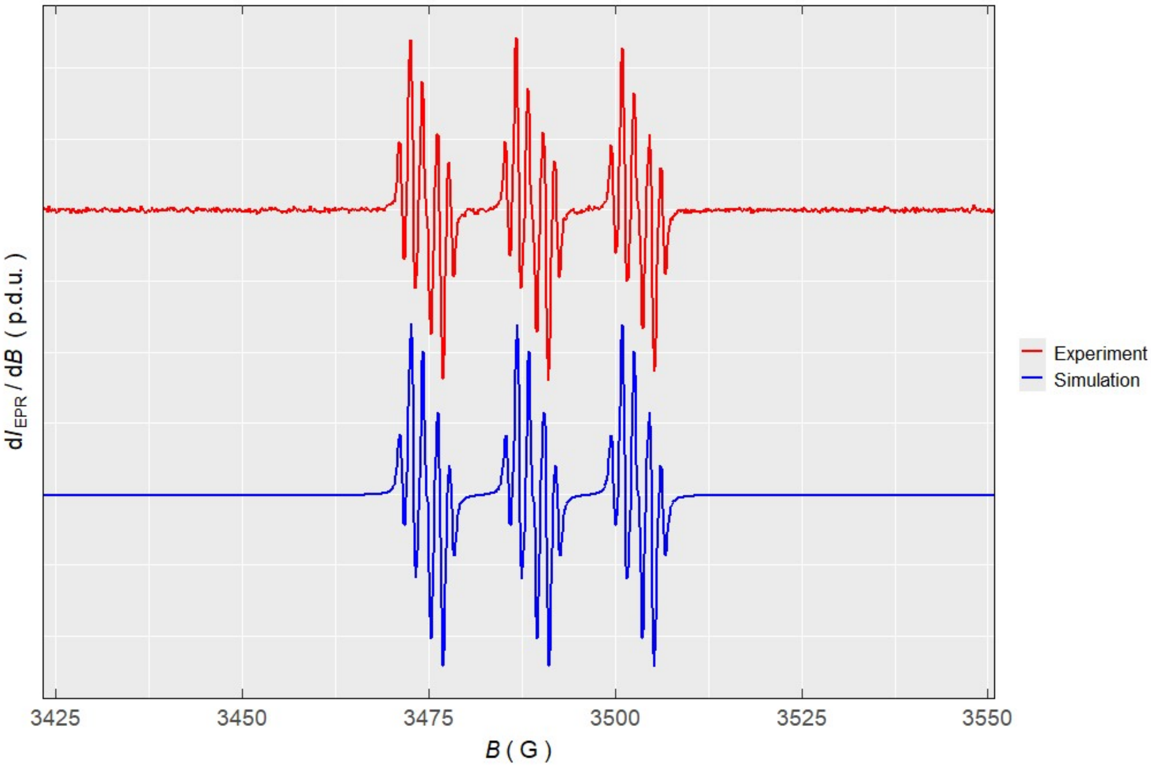

Figure S18. EPR spectrum of •PBN-CF_3 after irradiation of $Bu_4N[Cu(CF_3)_4]$ (c = 10 mM) with PBN (c = 10 mM), 1,3,5-trimethoxybenzene (c = 40 mM) and $(NH_4)_2S_2O_8$ (c = 40 mM) in MeCN. EPR simulation was performed by considering with the following hyperfine coupling constants

A(1 x 14N) = 39.82 (32.00) MHz, A(1 x 1H) = 5.82 (3.16) MHz, A(3 x 19F) = 4.21 (5.39) MHz. Couplings calculated by DFT (see EPR calculation for details) are presented in parentheses for comparison.

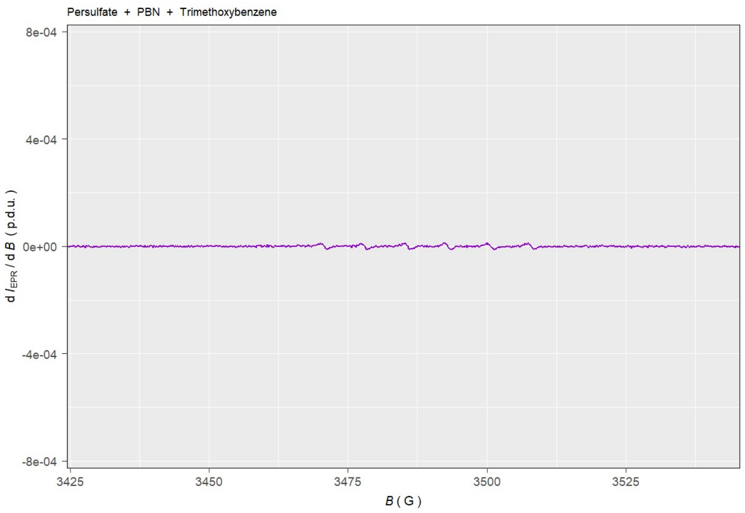

Figure S19. EPR spectrum after irradiation of PBN (c = 10 mM) with 1,3,5-trimethoxybenzene (c = 10 mM) and $(NH_4)_2S_2O_8$ (c = 10 mM) in MeCN.

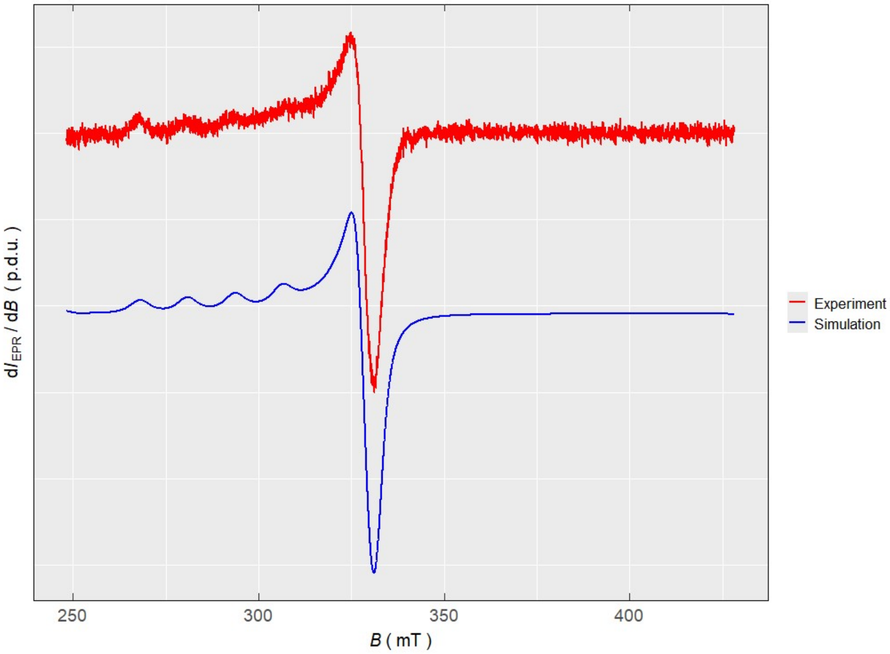

Figure S20. Comparison between the experimental and simulated EPR spectra of a Cu(II)-complex obtained at 77 K (liquid N_2) after irradiation of $Bu_4N[Cu(CF_3)_4]$ (c = 10 mM) with

$(NH_4)_2S_2O_8$ (c = 40 mM) in MeCN. EPR spectrum exhibit axial symmetry with $g_{||}$ = 2.37502, $g_{\perp}$ = 2.07786 and $A_{||}$(Cu(II)) = 414.98 MHz, $A_{\perp}$(Cu(II)) = 11.47 MHz, pointing to square-planar (octahedral) geometry of the formed Cu(II)-complex (see the crystallographic analysis vide infra).

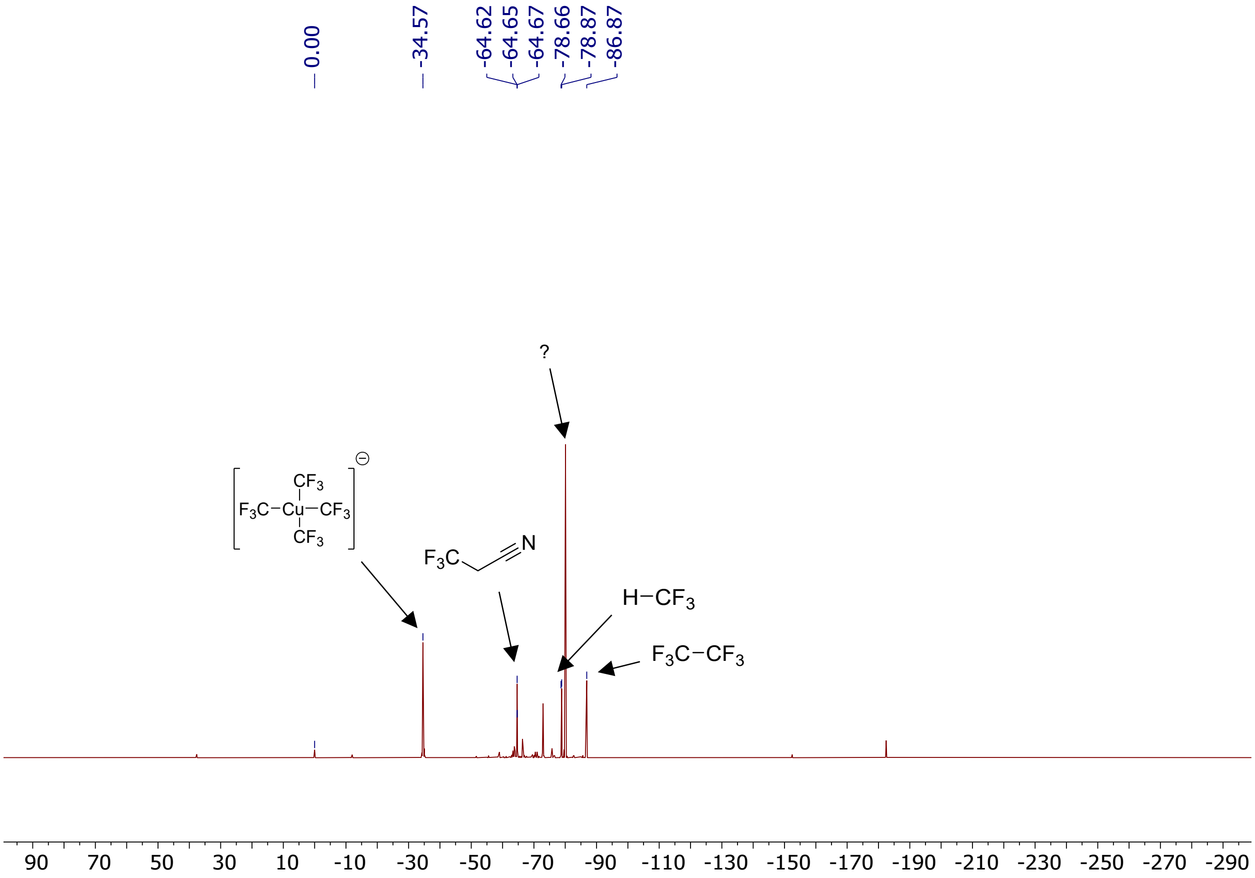

Figure S21. ^{19}F (376 MHz) NMR spectrum after irradiation of $Bu_4N[Cu(CF_3)_4]$ (c = 10 mM) with $(NH_4)_2S_2O_8$ (c = 40 mM) with 385 nm LED light, rt, 16 h in MeCN.

DFT-optimized structure of PBN-adduct:

A. PBN-CF_3 adduct

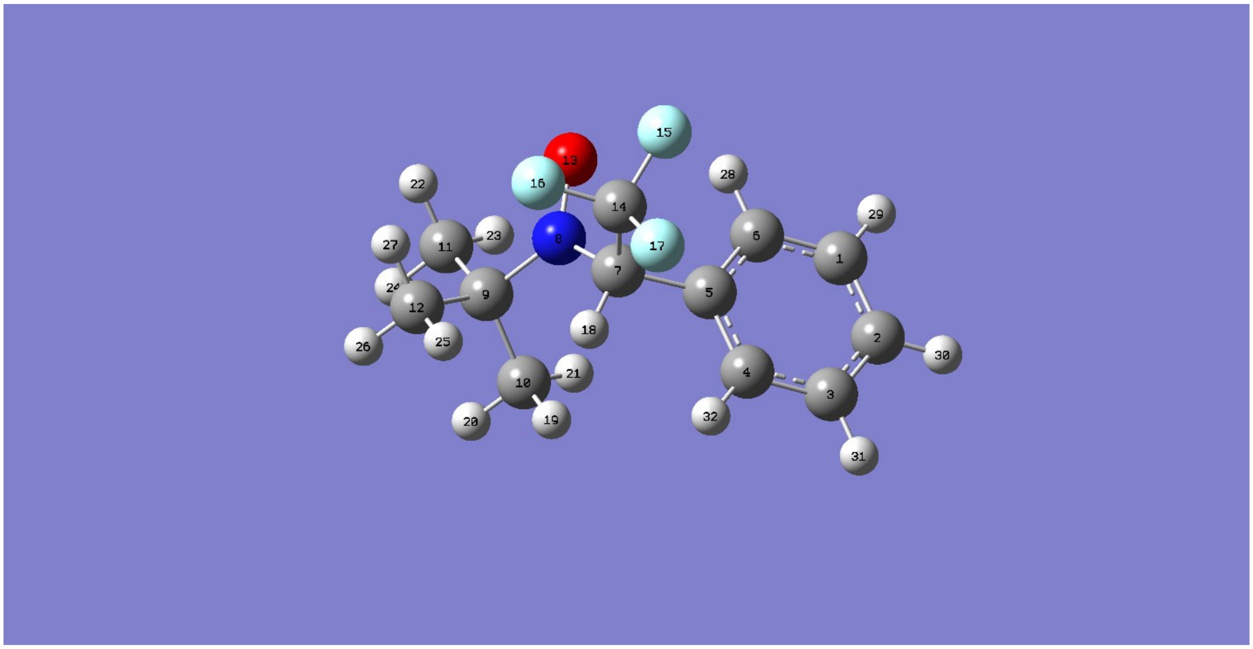

Figure S22. Optimized structure for PBN-CF_3 adduct. Atom numbering adopted from the Gaussian output (see also Table S5).

Table S5. Optimized geometry corresponding to cartesian coordinates for PBN-CF_3 adduct (see also Figure S21).

Number	Atom	X	Y	Z
1	C	3.066799	0.988789	1.254925
2	C	3.856863	1.220677	0.123794
3	C	3.338606	0.960312	-1.14812
4	C	2.036076	0.472123	-1.28594
5	C	1.241151	0.235623	-0.1553
6	C	1.766143	0.49606	1.119362
7	C	-0.1755	-0.29904	-0.3547
8	N	-1.20105	0.413035	0.424821
9	C	-2.18082	1.359333	-0.23119

**10**	C	-1.40382	2.394843	-1.06422
**11**	C	-2.96813	2.068924	0.874457
**12**	C	-3.13878	0.546495	-1.12298
**13**	O	-1.25636	0.17725	1.688484
**14**	C	-0.26278	-1.81605	-0.09417
**15**	F	0.208758	-2.21568	1.107212
**16**	F	-1.53618	-2.27295	-0.19101
**17**	F	0.463533	-2.48405	-1.03233
**18**	H	-0.43362	-0.20132	-1.41044
**19**	H	-0.84642	1.936882	-1.88636
**20**	H	-2.11679	3.099743	-1.50386
**21**	H	-0.7058	2.958759	-0.43757
**22**	H	-3.54065	1.362871	1.480325
**23**	H	-2.30712	2.636773	1.535184
**24**	H	-3.66631	2.765945	0.401227
**25**	H	-2.61106	0.028629	-1.92992
**26**	H	-3.86311	1.226553	-1.58323
**27**	H	-3.68816	-0.19266	-0.53157
**28**	H	1.15746	0.321731	1.999909
**29**	H	3.462517	1.190293	2.24646
**30**	H	4.867741	1.603264	0.233537
**31**	H	3.942339	1.140812	-2.03305
**32**	H	1.633698	0.277715	-2.27666

Crystallographic data

Single-crystal diffraction data of **3m** were collected using Bruker D8 VENTURE system equipped with a Photon 100 CMOS detector and a CuKα Incoatec microfocus sealed tube (λ = 1.54178 Å) at 180 K. The frames were integrated with the with Bruker SAINT[21] software package.

Single-crystal diffraction data of **6** and **20** were collected on a Rigaku HF007 diffractometer equipped with a rotating copper anode (Cu Kα radiation, λ = 1.54184 Å) and a HyPix-6000HE

hybrid photon counting detector at 100 K. The data were collected and processed using CrysAlisPro[22] software.

The structures were solved by charge-flipping methods using Superflip[23] and were refined by full-matrix least-squares on F^2 with CRYSTALS.[24] The positional and anisotropic thermal parameters of all non-hydrogen atoms were refined. Hydrogen atoms were initially located in the difference Fourier map but those attached to carbon atoms were recalculated into idealized positions and refined with riding constraints.

*Crystal data for **3m*** (colorless, 0.064 × 0.093 × 0.147 mm): $C_{20}H_{24}F_6$, triclinic, space group *P*-1, a = 7.1432(4) Å, b = 8.2660(5) Å, c = 8.5093(5) Å, α = 77.131(3)°, β = 68.244(2)°, γ = 454.86(5)°, V = 454.86(5) Å^3, Z = 1, M = 378.39, 12479 reflections measured, 1663 independent reflections. Final R = 0.0427, wR = 0.1193, GoF = 0.9755 for 1448 reflections with $I > 2\sigma(\mathrm{I})$ and 118 parameters. CCDC 2474866.

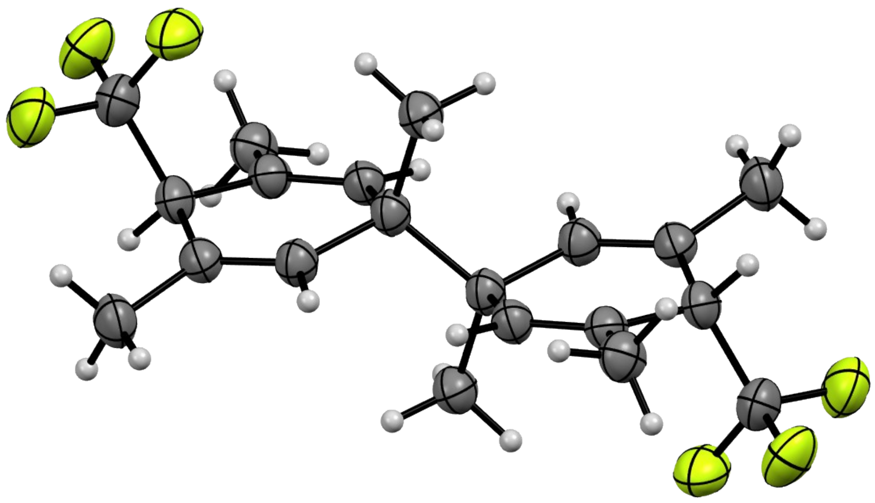

Figure S23. ORTEP diagram of **3m**.

*Crystal data for **6*** (colorless, 0.027 × 0.035 × 0.105 mm): $C_{10}H_{11}F_3N_4O_2$, monoclinic, space group $P2_1/c$, a = 9.6718(2) Å, b = 7.81519(14) Å, c = 15.5844(4) Å, β = 98.285(2)°, V = 1165.68(4) Å^3, Z = 4, M = 276.22, 8456 reflections measured, 2433 independent reflections. Final R = 0.0463, wR = 0.1240, GoF = 0.9849 for 1991 reflections with $I > 2\sigma(I)$ and 173 parameters. CCDC 2474867.

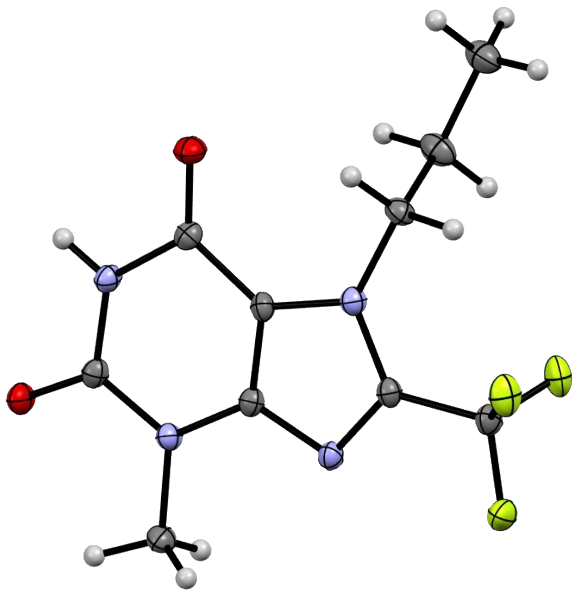

Figure S24. ORTEP diagram of **6**.

Crystal data for ***20*** (light blue, 0.091 × 0.201 × 0.345 mm): $CuH_{12}O_6.2(SO_4).2(NH_4)$, monoclinic, space group $P2_1/c$, $a = 6.35117(16)$ Å, $b = 12.2470(3)$ Å, $c = 9.1130(2)$ Å, $\beta = 106.270(3)°$, $V = 680.44(3)$ Å^3, $Z = 2$, $M = 399.85$, 9577 reflections measured, 1435 independent reflections. Final $R = 0.0269$, $wR = 0.0733$, $GoF = 0.9991$ for 1364 reflections with $I > 2\sigma(I)$ and 88 parameters. CCDC 2474868.

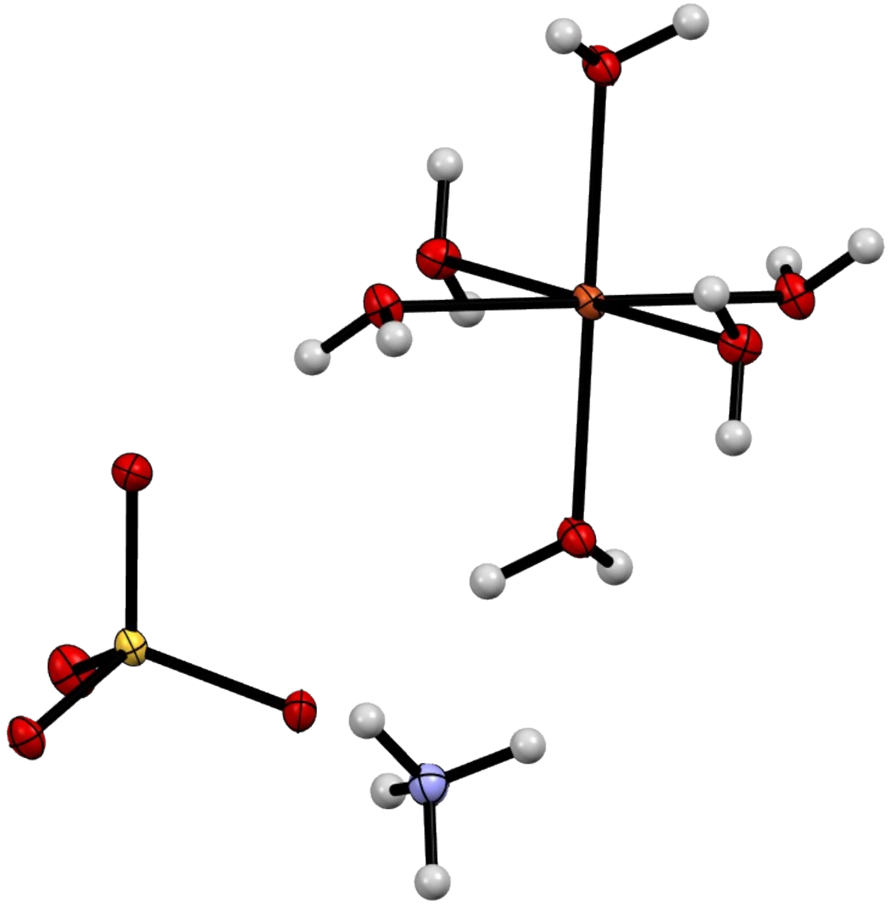

Figure S25. ORTEP diagram of **20**.

On/Off experiment

An oven-dried Schlenk flask was charged with tetrabutylammonium tetrakis(trifluoromethyl)cuprate(III) (59 mg, 0.1 mmol, 0.25 equiv.), 1,3,5-trimethoxybenzene (68 mg, 0.4 mmol, 1 equiv.) and $(NH_4)_2S_2O_8$ (92 mg, 0.4 mmol, 1 equiv.) then it was evacuated and backfilled with nitrogen (3×). DMSO (1 ml) and internal standard (Trifluoromethyl)benzene (13 μL) were added under inert atmosphere. LED light (λ_{max} = 387 nm) (see Figure S2) was introduced into the flask via a glass rod and after sealing under nitrogen atmosphere. Starting with lights turned on, aliquots of 40 μL were taken every 15 min. In the same interval, the light was turned on or off, respectively. The aliquots were diluted with $CDCl_3$, filtered through cotton and analyzed by ^{19}F NMR spectroscopy to determine the yield of product **3a**.

Table S6. Yield *versus* time profile for formation of **3a** in the on/off experiment.

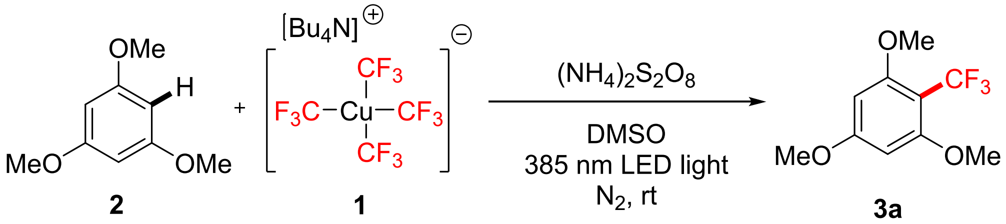

Entry	Light	*t* (min)	Yield of **3a** (%)
1	On	15	43
2	Off	30	43
3	On	45	75
4	Off	60	75
5	On	75	83
6	Off	90	83

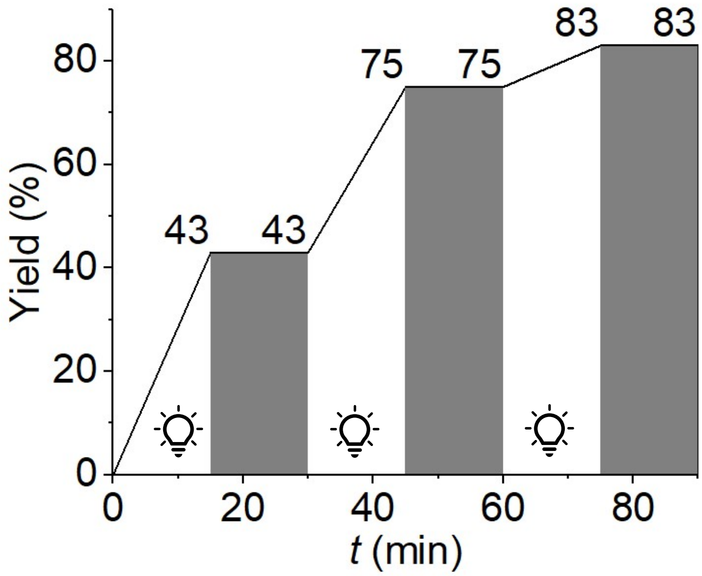

Figure S26. On/off experiment showing the influence of the visible light irradiation on the formation of **3a**.

References

[1] V. Motornov, M. Procházka, N. Alpuente, P. Salvador, P. Slavíček, B. Klepetářová, X. Ribas, P. Beier, "Introducing Weakly Ligated Tris(trifluoromethyl)copper(III)" *ChemistryEurope* **2024**, *2*, e202400004.

[2] S. Seo, J. B. Taylor, M. F. Greaney, "Silver-catalysed trifluoromethylation of arenes at room temperature" *Chem. Commun.* **2013**, *49*, 6385.

[3] K. Natte, R. V. Jagadeesh, L. He, J. Rabeah, J. Chen, C. Taeschler, S. Ellinger, F. Zaragoza, H. Neumann, A. Brückner, M. Beller, "Palladium-Catalyzed Trifluoromethylation of (Hetero)Arenes with CF_3Br" *Angew. Chem., Int. Ed.* **2016**, *55*, 2782–2786.

[4] A. Lishchynskyi, M. A. Novikov, E. Martin, E. C. Escudero-Adán, P. Novák, V. V. Grushin, "Trifluoromethylation of Aryl and Heteroaryl Halides with Fluoroform-Derived $CuCF_3$: Scope, Limitations, and Mechanistic Features" *J. Org. Chem.* **2013**, *78*, 11126–11146.

[5] Y. Ye, S. H. Lee, M. S. Sanford, "Silver-Mediated Trifluoromethylation of Arenes Using $TMSCF_3$" *Org. Lett.* **2011**, *13*, 5464–5467.

[6] J. Castaner, J. Riera, E. Molins, M. Mas, "Synthesis, UV-spectrum and molecular structure of 3,3',5,5'-tetrachloro-4,4'-bis(trichloromethyl)biphenyl, a dimeric analogue of α,α,α,2,4,6-hexachlorotoluene" *Anales de Química* **1994**, *90*, 387–396.

[7] O. Jacobson, Y. Bechor, A. Icar, N. Novak, A. Birman, H. Marom, L. Fadeeva, E. Golan, I. Leibovitch, M. Gutman, E. Even-Sapir, R. Chisin, M. Gozin, E. Mishani, "Prostate cancer PET bioprobes: Synthesis of [^{18}F]-radiolabeled hydroxyflutamide derivatives" *Bioorg. Med. Chem.* **2005**, *13*, 6195–6205.

[8] P. Liu, W. Liu, C.-J. Li, "Catalyst-Free and Redox-Neutral Innate Trifluoromethylation and Alkylation of Aromatics Enabled by Light" *J. Am. Chem. Soc.* **2017**, *139*, 14315–14321.

[9] I. Ghosh, J. Khamrai, A. Savateev, N. Shlapakov, M. Antonietti, B. König, "Organic semiconductor photocatalyst can bifunctionalize arenes and heteroarenes" *Science* **2019**, *365*, 360–366.

[10] Y. Kobayashi, K. Yamamoto, T. Asai, M. Nakano, I. Kumadaki, "Studies on organic fluorine compounds. Part 35. Trifluoromethylation of pyrimidine- and purine-nucleosides with trifluoromethyl–copper complex" *J. Chem. Soc., Perkin Trans. 1* **1980**, 2755–2761.

[11] Y. Huang, Y.-Y. Lei, L. Zhao, J. Gu, Q. Yao, Z. Wang, X.-F. Li, X. Zhang, C.-Y. He, "Catalyst-free and visible light promoted trifluoromethylation and perfluoroalkylation of uracils and cytosines" *Chem. Commun.* **2018**, *54*, 13662–13665.

[12] M. P. Mertes, S. E. Saheb, "5-Trifluoromethyl-6-azauracil" *J. Heterocycl. Chem.* **1965**, *2*, 491–491.

[13] S. Fernández-García, V. O. Chantzakou, F. Juliá-Hernández, "Direct Decarboxylation of Trifluoroacetates Enabled by Iron Photocatalysis**" *Angew. Chem., Int. Ed.* **2024**, *63*, e202311984.

[14] S. T. Shreiber, G. I. Puchall, D. A. Vicic, “Transformation of brucine into trifluoromethyl neobrucine using the homoleptic nickel catalyst $[Ni(CF_3)_4]^{2-}$” *Tetrahedron Lett.* **2022**, *97*, 153795.

[15] D. M. Grant, R. Kollrack, “Absorption spectra and configuration of the ammine complexes of copper (II)” *J. Inorg. Nucl. Chem.* **1961**, *23*, 25–29.

[16] S. Stoll, A. Schweiger, “EasySpin, a comprehensive software package for spectral simulation and analysis in EPR” *J. Magnetic Resonance* **2006**, *178*, 42–55.

[17] M. J. Frisch, G. W. Trucks, H. B. Schlegel, G. E. Scuseria, M. A. Robb, J. R. Cheeseman, G. Scalmani, V. Barone, G. A. Petersson, H. Nakatsuji, X. Li, M. Caricato, A. V. Marenich, J. Bloino, B. G. Janesko, R. Gomperts, B. Mennucci, H. P. Hratchian, J. V. Ortiz, A. F. Izmaylov, J. L. Sonnenberg, D. Williams-Young, F. Ding, F. Lipparini, F. Egidi, J. Goings, B. Peng, A. Petrone, T. Henderson, D. Ranasinghe, V. G. Zakrzewski, J. Gao, N. Rega, G. Zheng, W. Liang, M. Hada, M. Ehara, K. Toyota, R. Fukuda, J. Hasegawa, M. Ishida, T. Nakajima, Y. Honda, O. Kitao, H. Nakai, T. Vreven, K. Throssell, J. A. Montgomery, Jr., J. E. Peralta, F. Ogliaro, M. J. Bearpark, J. J. Heyd, E. N. Brothers, K. N. Kudin, V. N. Staroverov, T. A. Keith, R. Kobayashi, J. Normand, K. Raghavachari, A. P. Rendell, J. C. Burant, S. S. Iyengar, J. Tomasi, M. Cossi, J. M. Millam, M. Klene, C. Adamo, R. Cammi, J. W. Ochterski, R. L. Martin, K. Morokuma, O. Farkas, J. B. Foresman, D. J. Fox, Gaussian 16, Revision A.03, Gaussian, Inc., Wallingford CT, 2016.

[18] A. V. Marenich, C. J. Cramer, D. G. Truhlar, “Universal Solvation Model Based on Solute Electron Density and on a Continuum Model of the Solvent Defined by the Bulk Dielectric Constant and Atomic Surface Tensions” *J. Phys. Chem. B* **2009**, *113*, 6378–6396.

[19] V. Barone, M. Cossi, “Quantum Calculation of Molecular Energies and Energy Gradients in Solution by a Conductor Solvent Model” *J. Phys. Chem. A* **1998**, *102*, 1995–2001.

[20] M. Cossi, N. Rega, G. Scalmani, V. Barone, “Energies, structures, and electronic properties of molecules in solution with the C-PCM solvation model” *J. Comput. Chem.* **2003**, *24*, 669–681.

[21] *SAINT. Bruker AXS Inc., Madison, Wisconsin, USA, 2015.*

[22] *CrysAlisPro, Rigaku Oxford Diffraction, 2024.*

[23] A. Altomare, G. Cascarano, C. Giacovazzo, A. Guagliardi, M. C. Burla, G. Polidori, M. Camalli, “*SIRPOW* .92 – a program for automatic solution of crystal structures by direct methods optimized for powder data” *J. Appl. Crystallogr.* **1994**, *27*, 435–436.

[24] P. W. Betteridge, J. R. Carruthers, R. I. Cooper, K. Prout, D. J. Watkin, “*CRYSTALS* version 12: software for guided crystal structure analysis” *J. Appl. Crystallogr.* **2003**, *36*, 1487–1487.

[25] A. Dang-Nguyen, K. C. Legaspi, C. T. McCarty, D. K. Smith and J. Gustafson. A Light- Promoted Innate Trifluoromethylation of Pyridones and Related N-Heteroarenes. *Org. Lett.*, **2023** *25*, 4898–4902.

[26] D. A. Nagib and D. W. MacMillan. Trifluoromethylation of arenes and heteroarenes by means of photoredox catalysis. *Nature* **2011**, 480, 224-228.

Copies of NMR spectra

^{1}H NMR spectrum of **3a** ($CDCl_3$, 400 MHz)

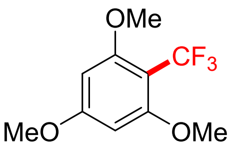

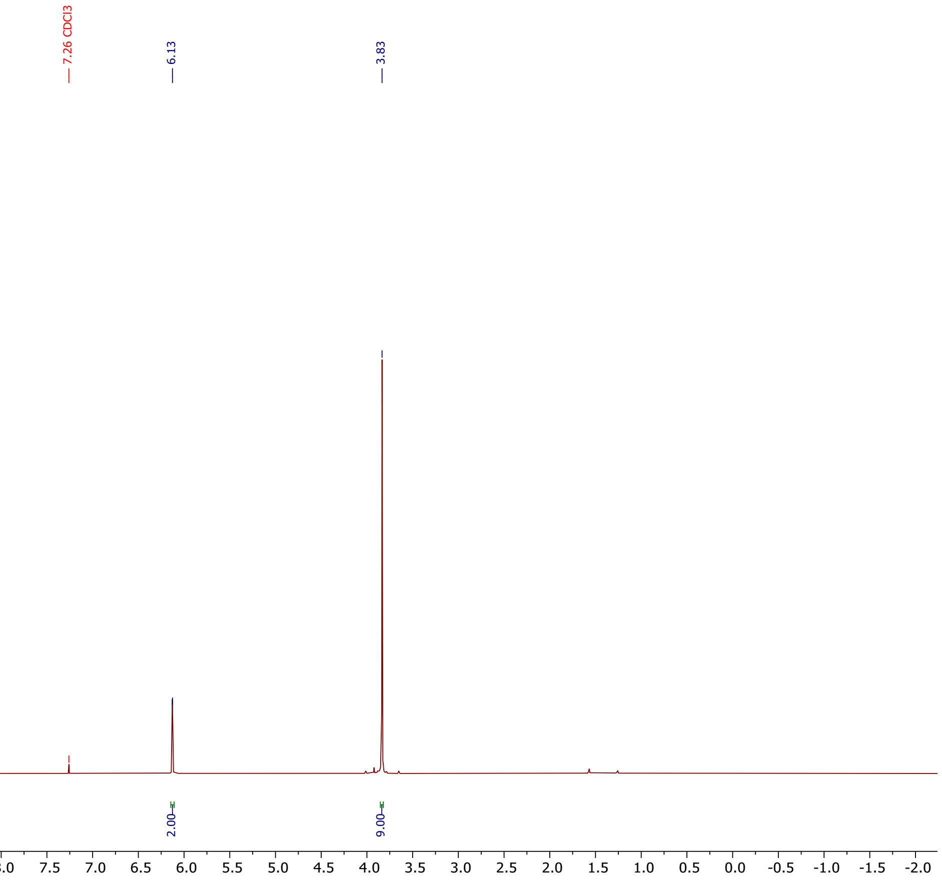

^{13}C NMR spectrum of **3a** ($CDCl_3$, 101 MHz)

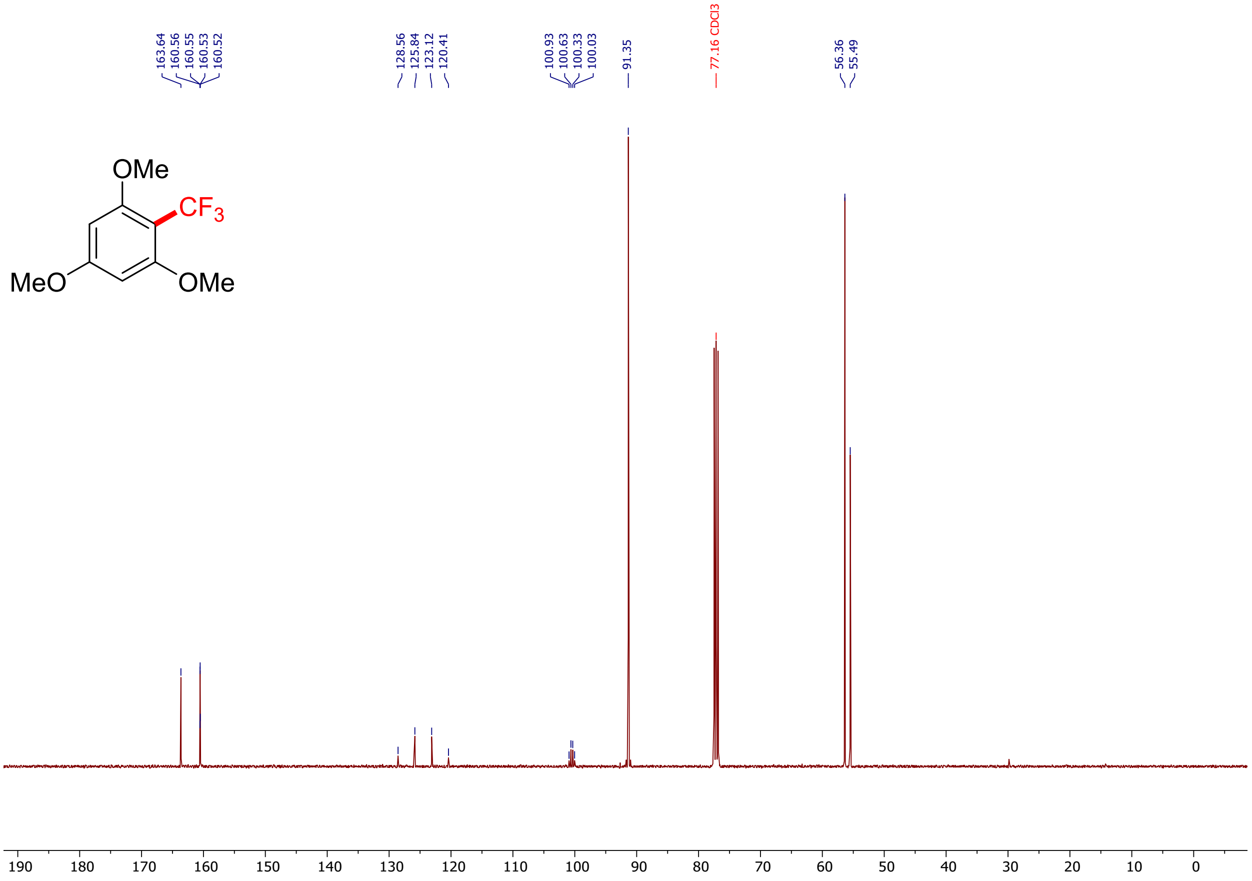

^{19}F NMR spectrum of **3a** ($CDCl_3$, 376 MHz)

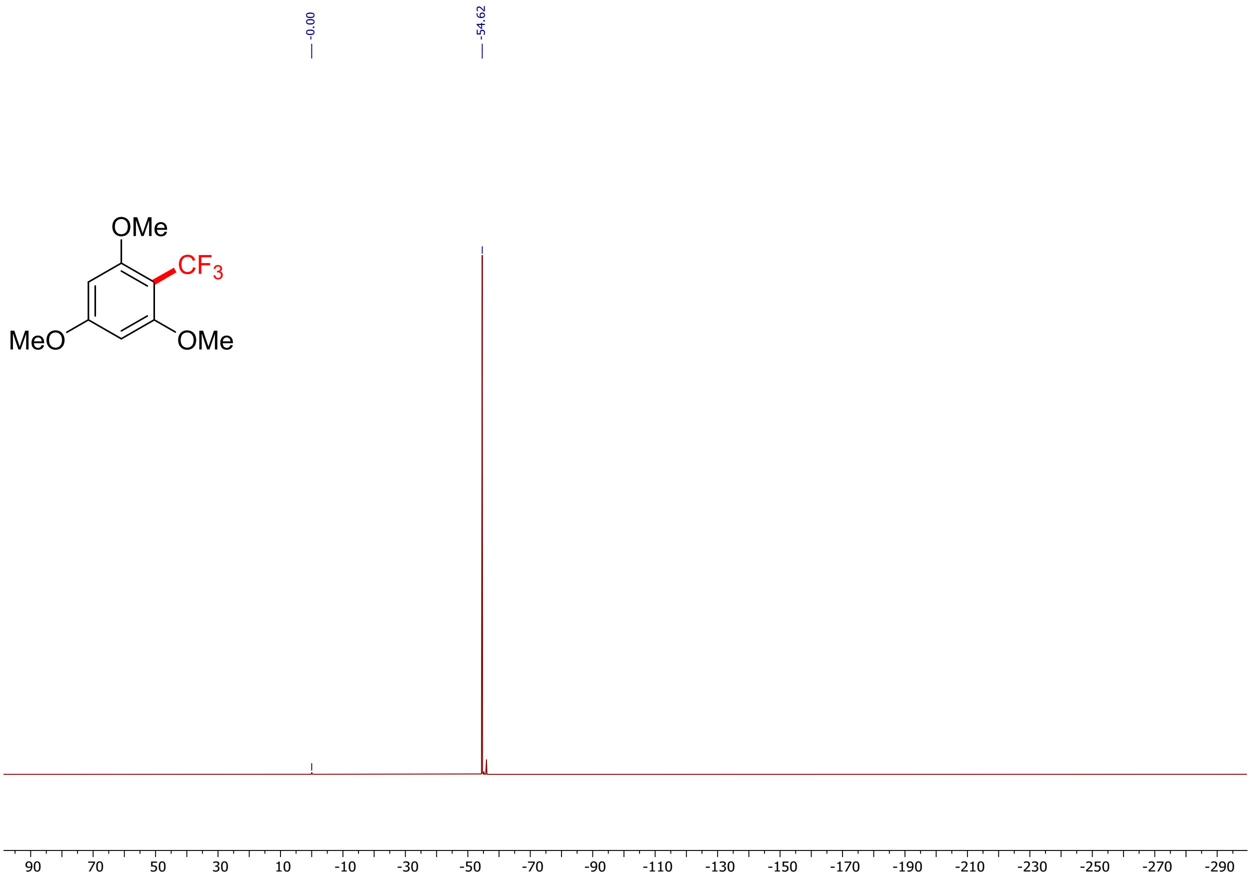

^{1}H NMR spectrum of **3b** ($CDCl_3$, 400 MHz)

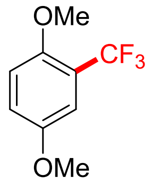

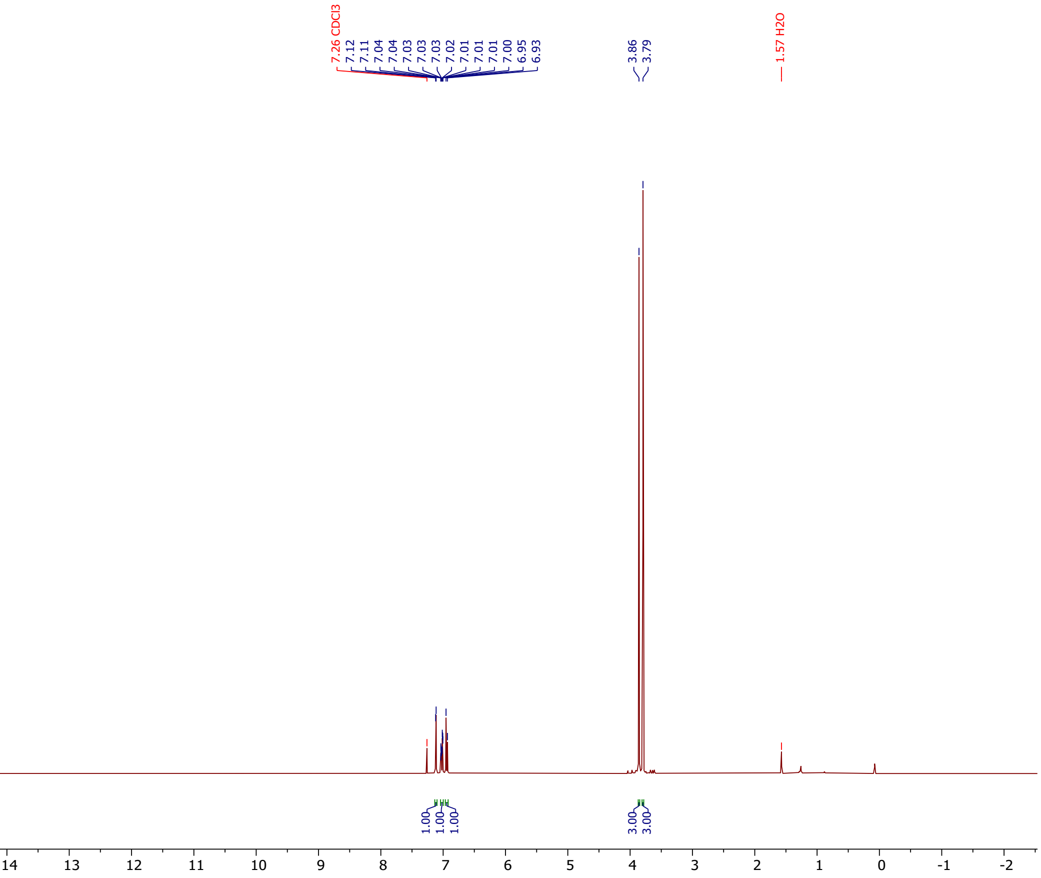

^{13}C NMR spectrum of **3b** ($CDCl_3$, 101 MHz)

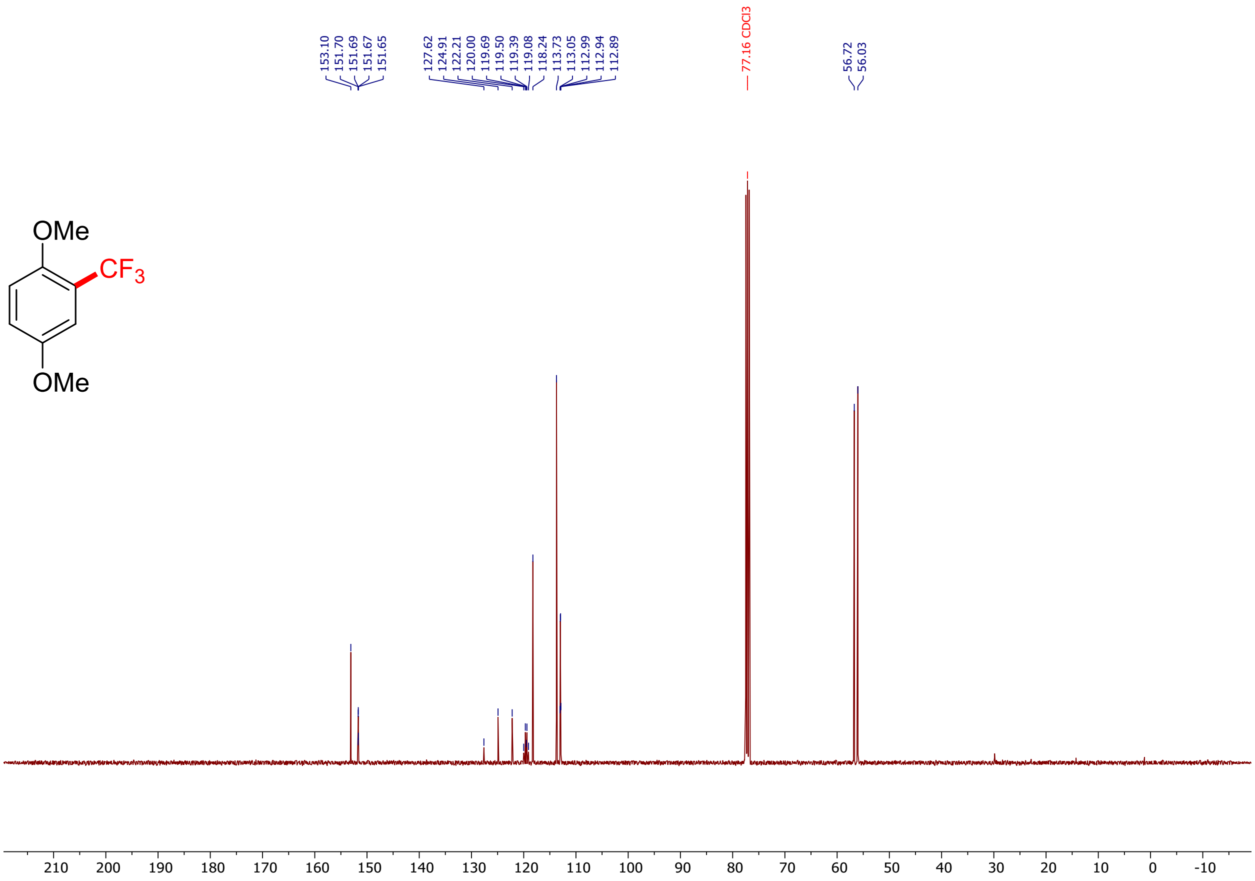

^{19}F NMR spectrum of **3b** ($CDCl_3$, 376 MHz)

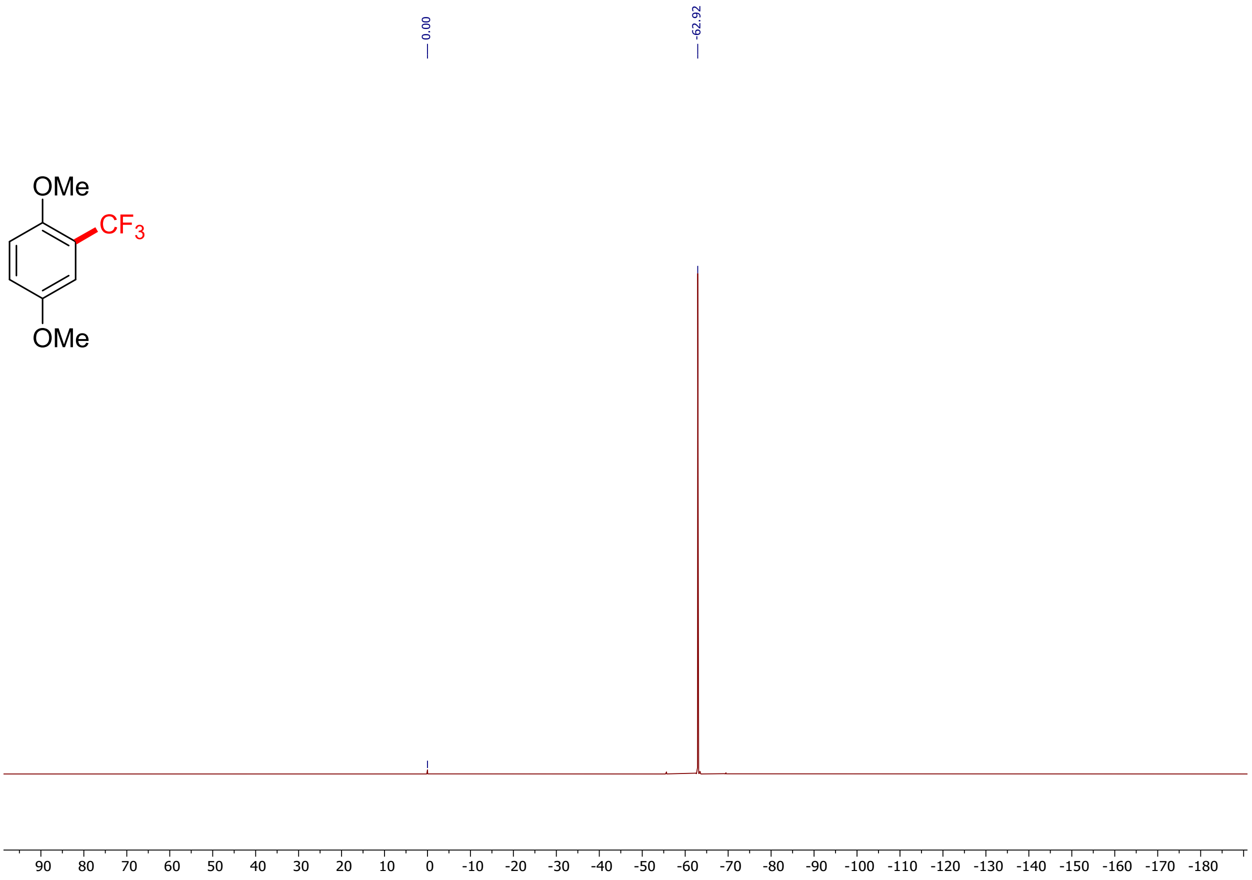

^{19}F NMR spectrum of crude **3c** ($CDCl_3$, 376 MHz)

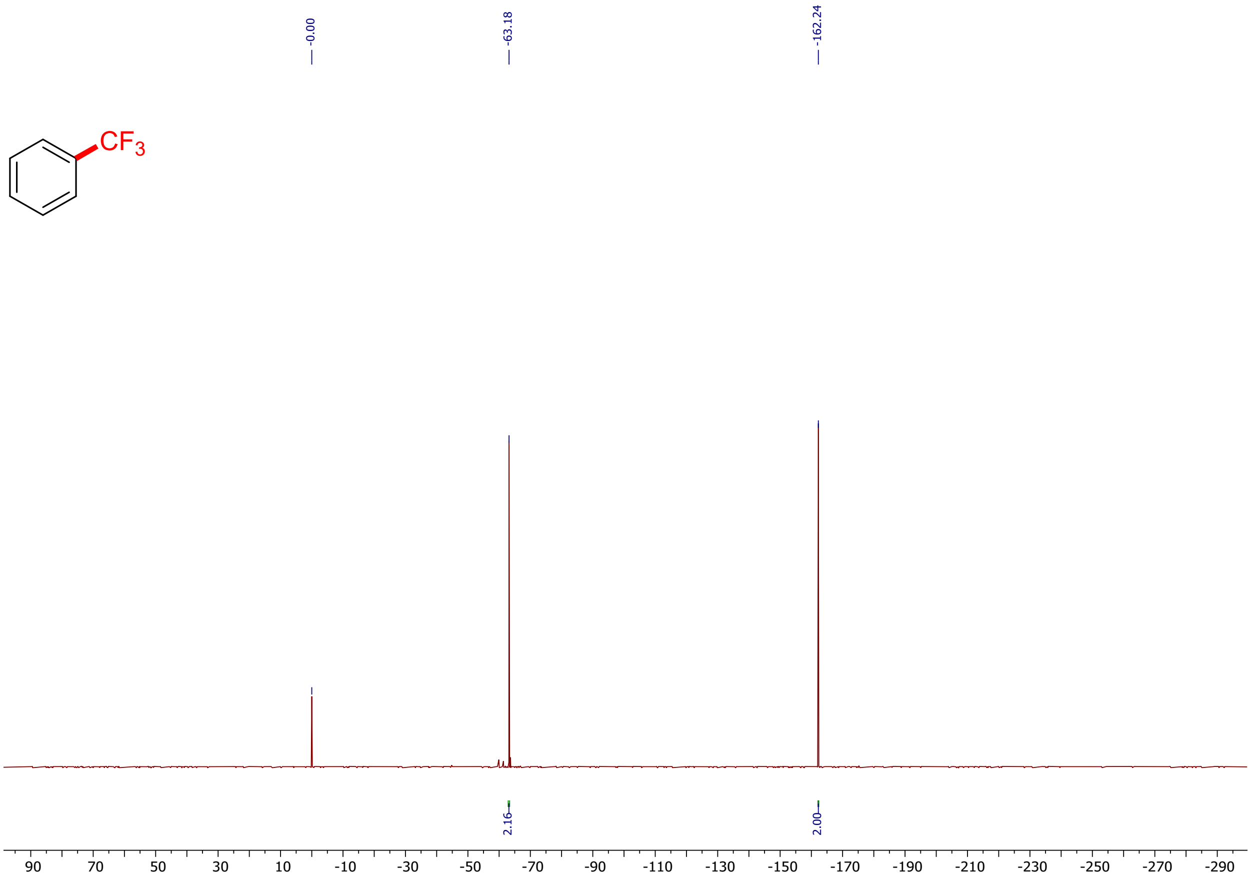

^{19}F NMR spectrum of crude **3d** ($CDCl_3$, 376 MHz)

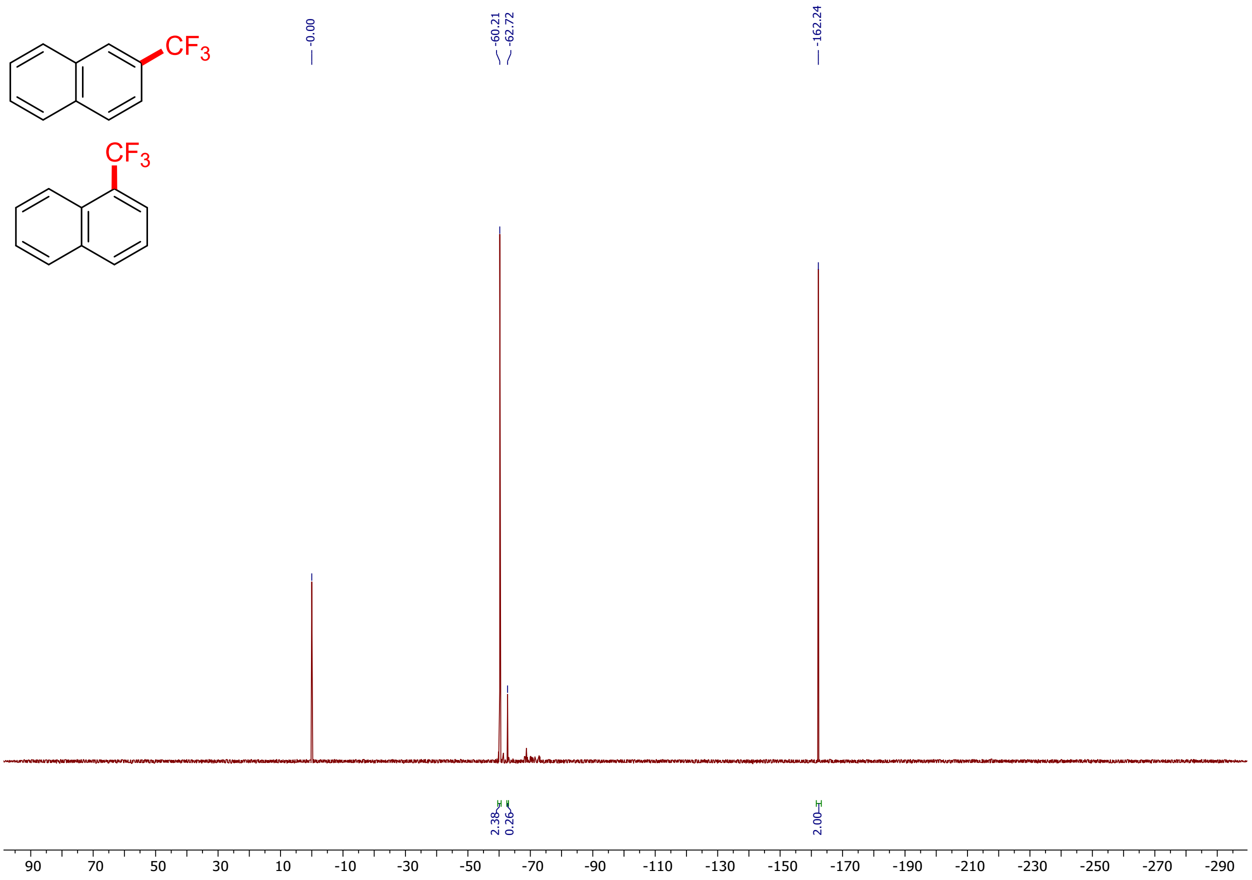

^1H NMR spectrum of **3e** ($CDCl_3$, 400 MHz)

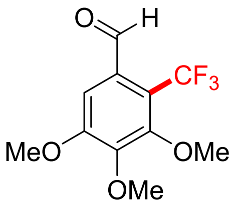

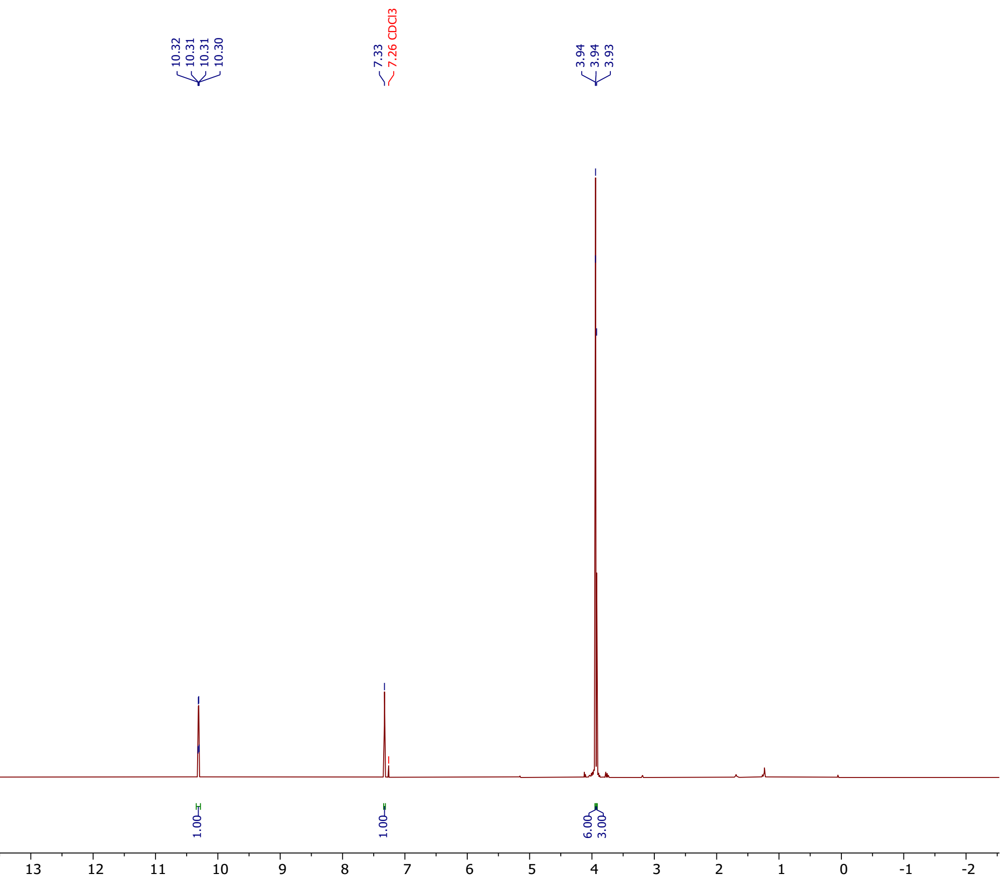

^{13}C NMR spectrum of **3e** ($CDCl_3$, 101 MHz)

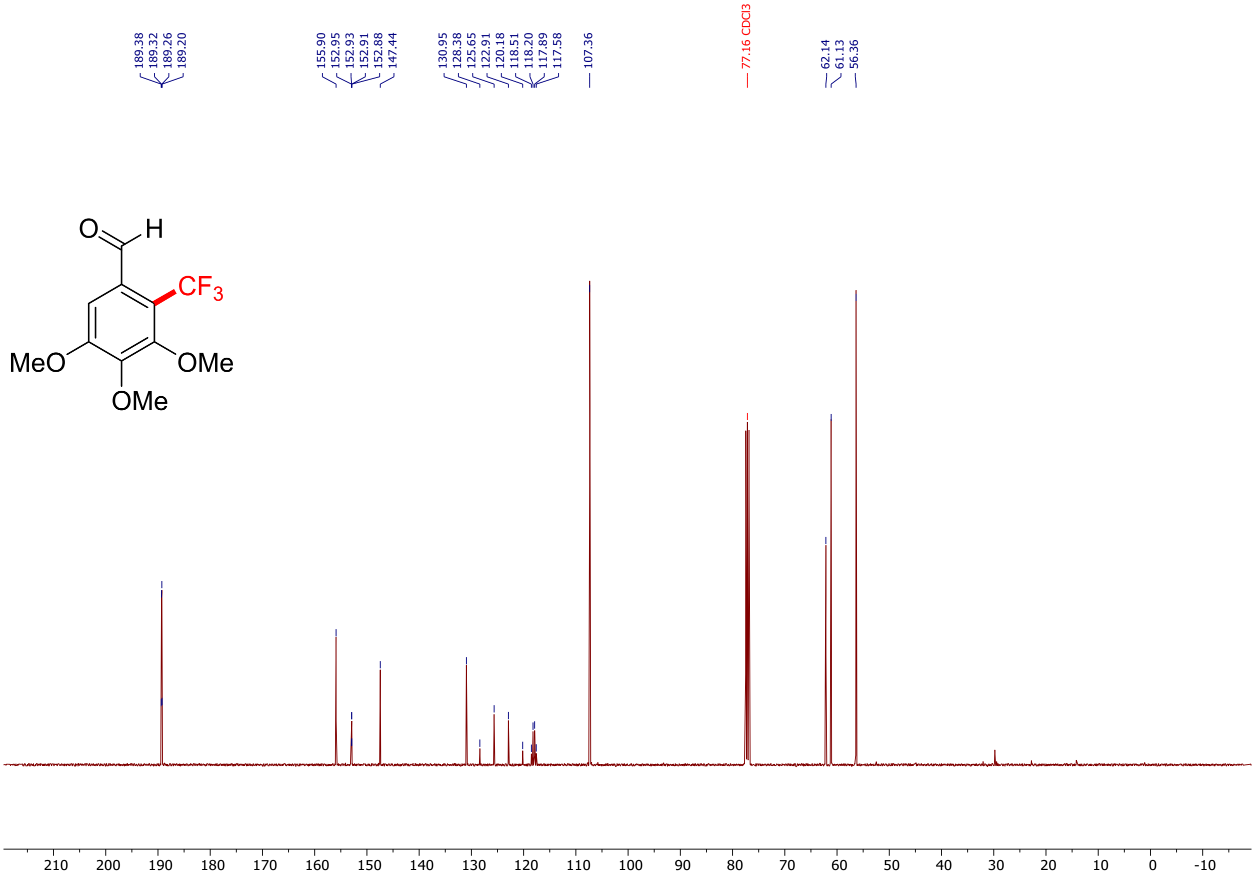

^{19}F NMR spectrum of **3e** ($CDCl_3$, 376 MHz)

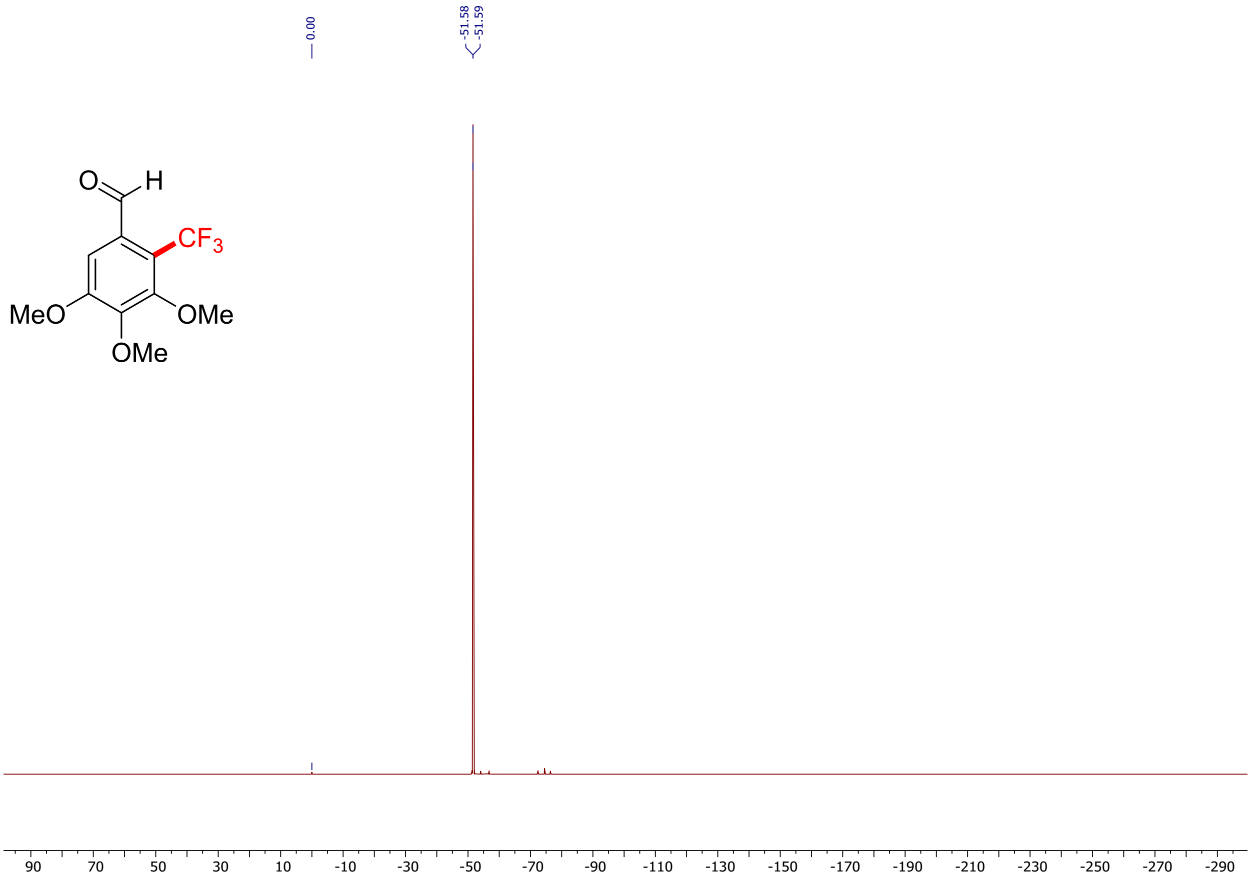

^{19}F NMR spectrum of crude **3f** ($CDCl_3$, 376 MHz)

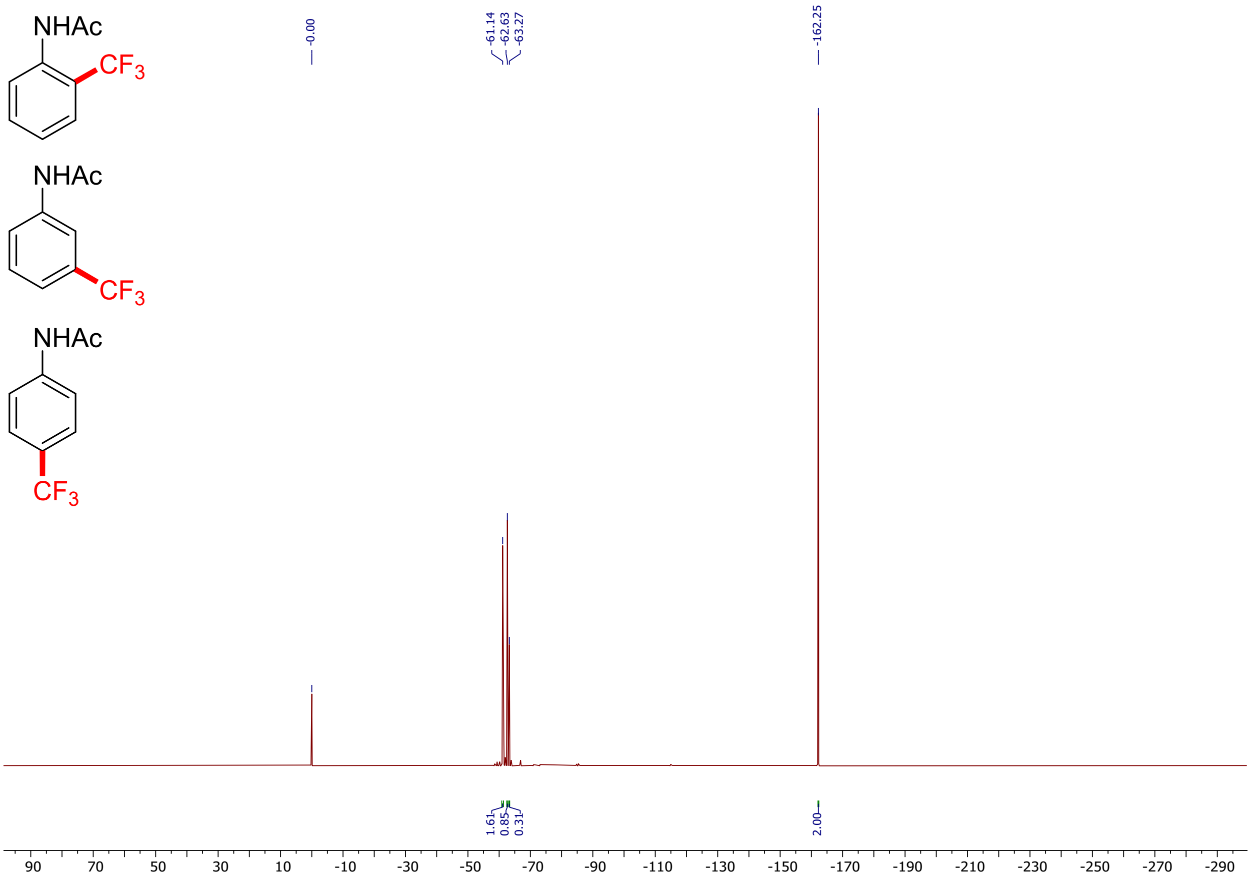

^{19}F NMR spectrum of crude **3g** ($CDCl_3$, 376 MHz)

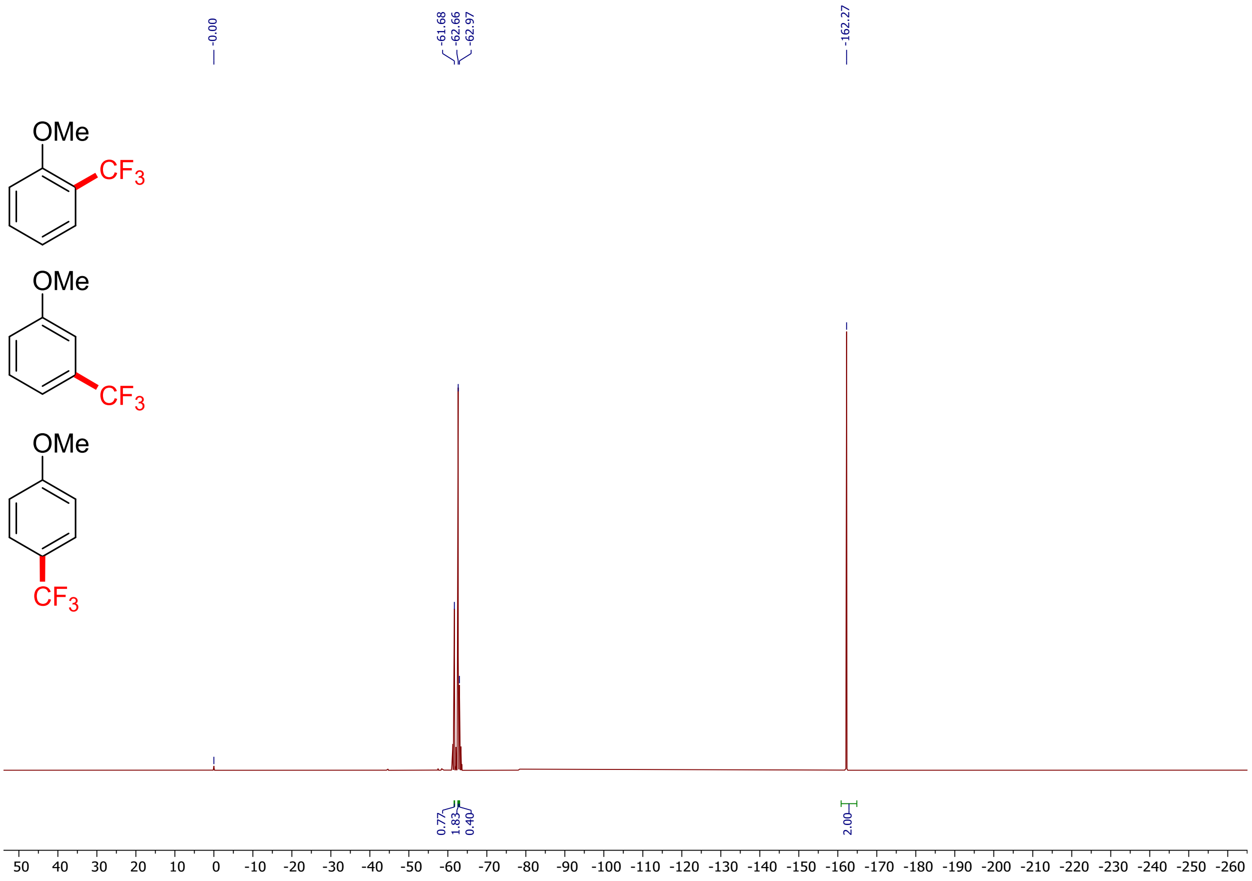

^{19}F NMR spectrum of crude **3h** ($CDCl_3$, 376 MHz)

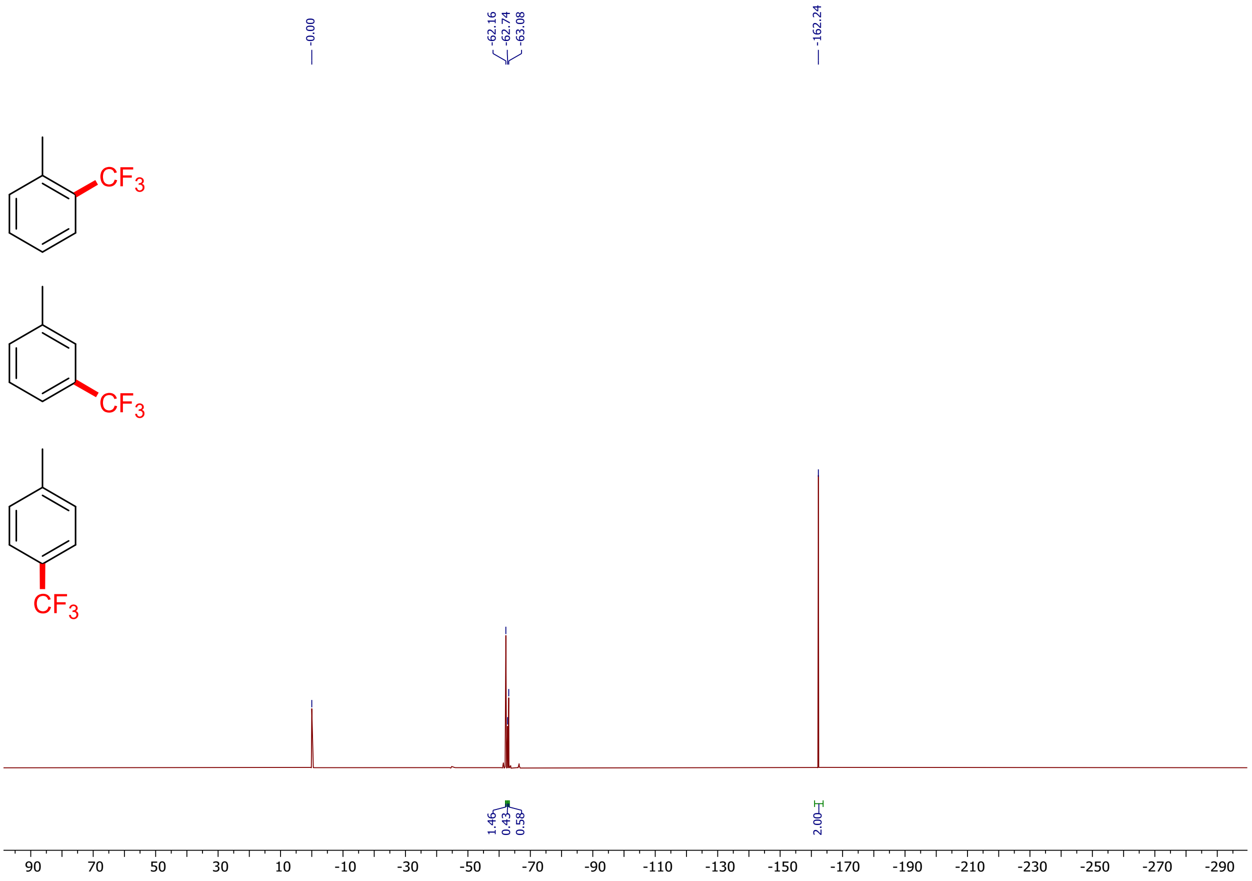

^{1}H NMR spectrum of **3i** ($CDCl_3$, 400 MHz)

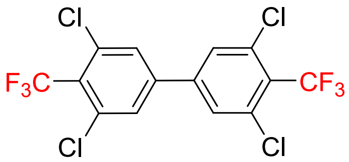

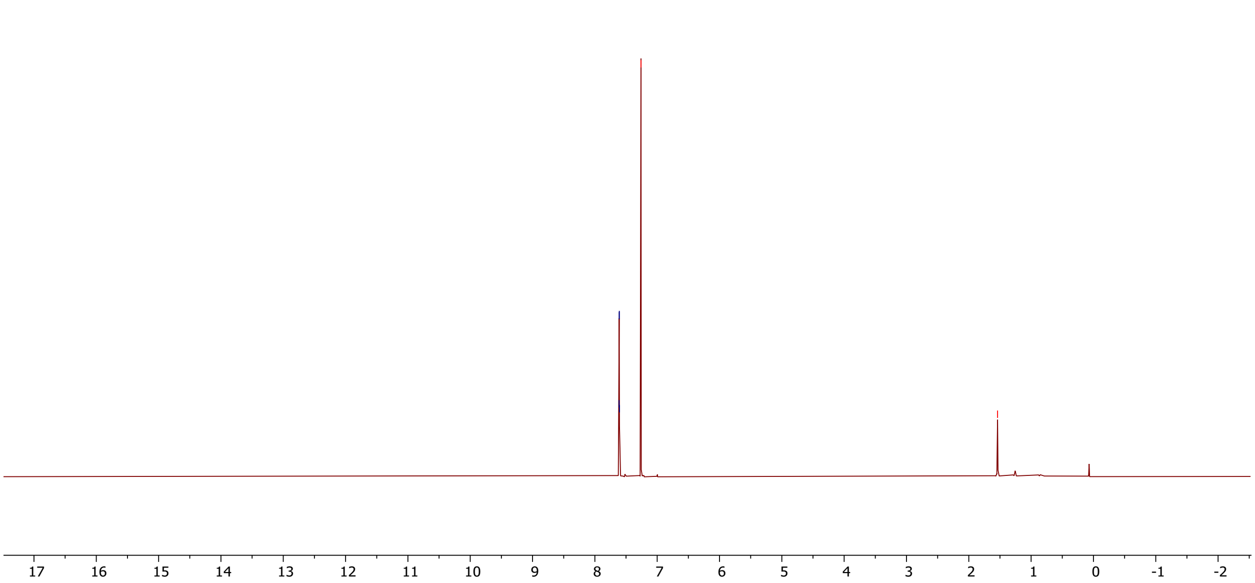

^{13}C NMR spectrum of **3i** ($CDCl_3$, 101 MHz)

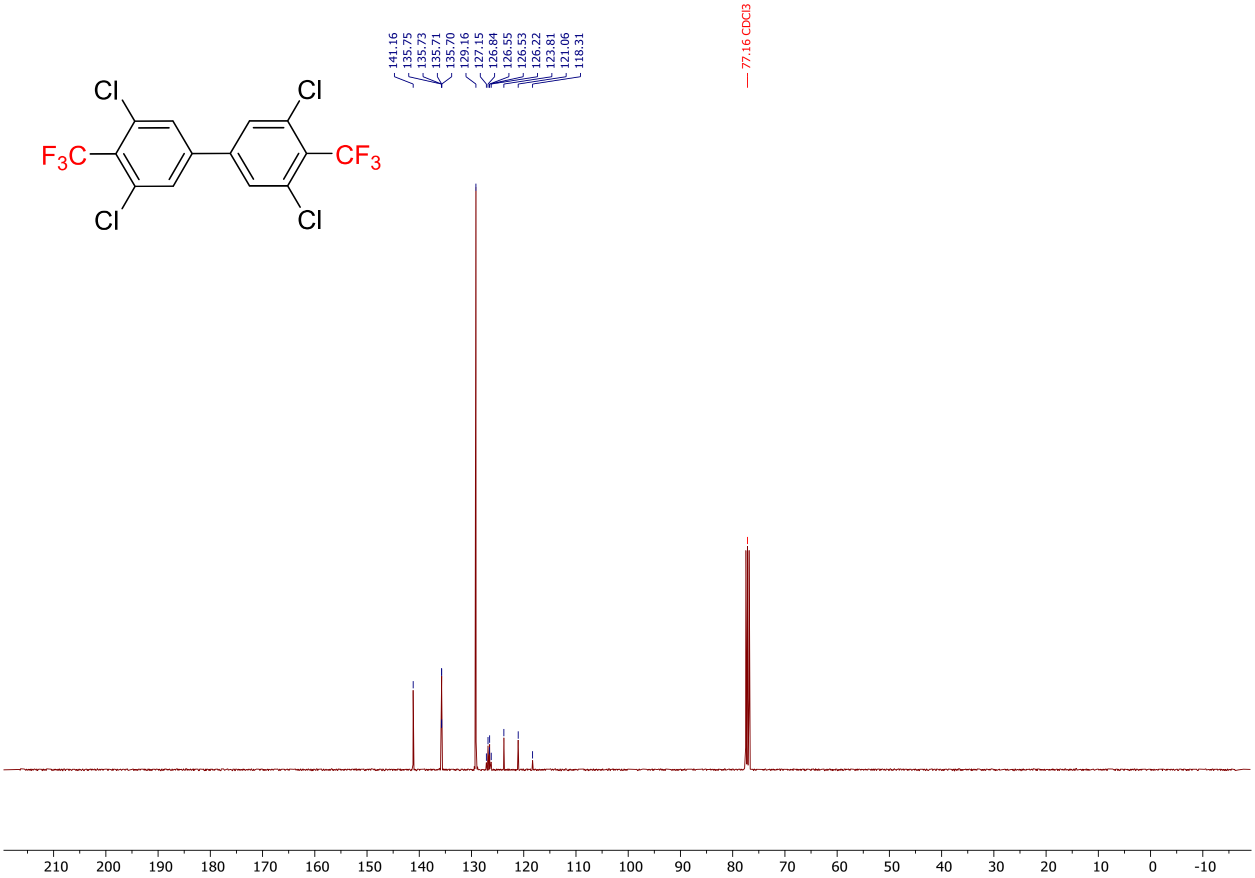

^{19}F NMR spectrum of **3i** ($CDCl_3$, 376 MHz)

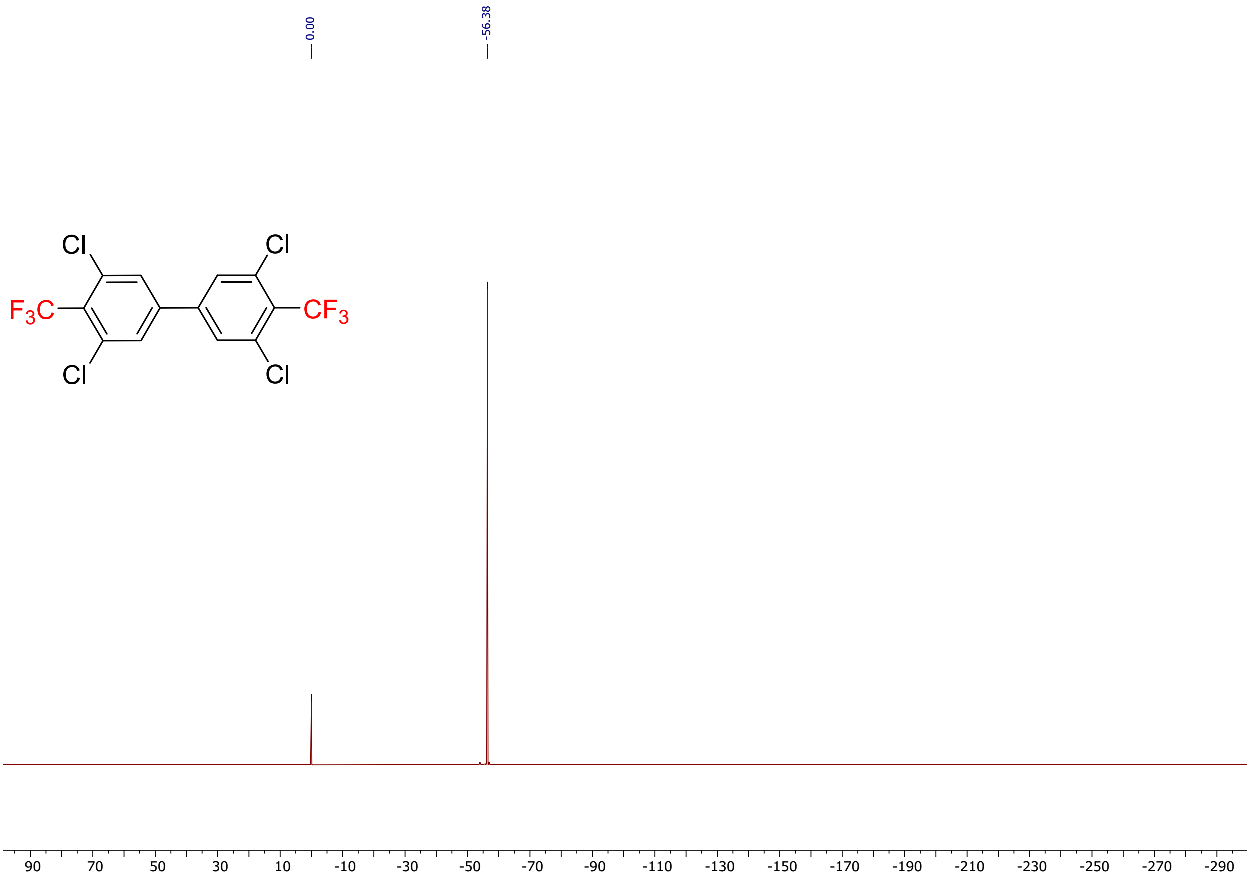

^{19}F NMR spectrum of crude **3j** ($CDCl_3$, 376 MHz)

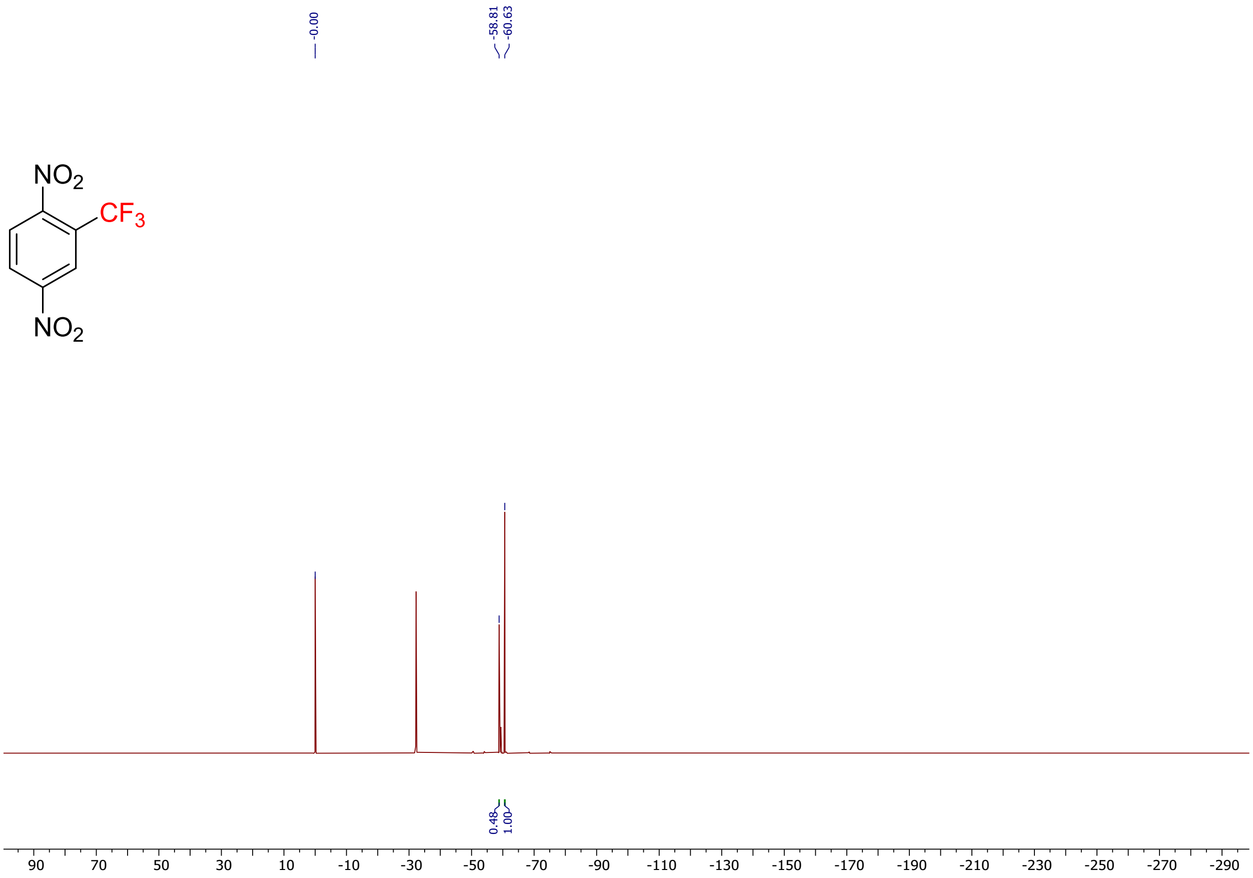

^1H NMR spectrum of **3k** ($CDCl_3$, 400 MHz)

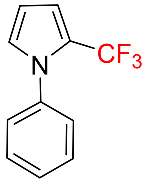

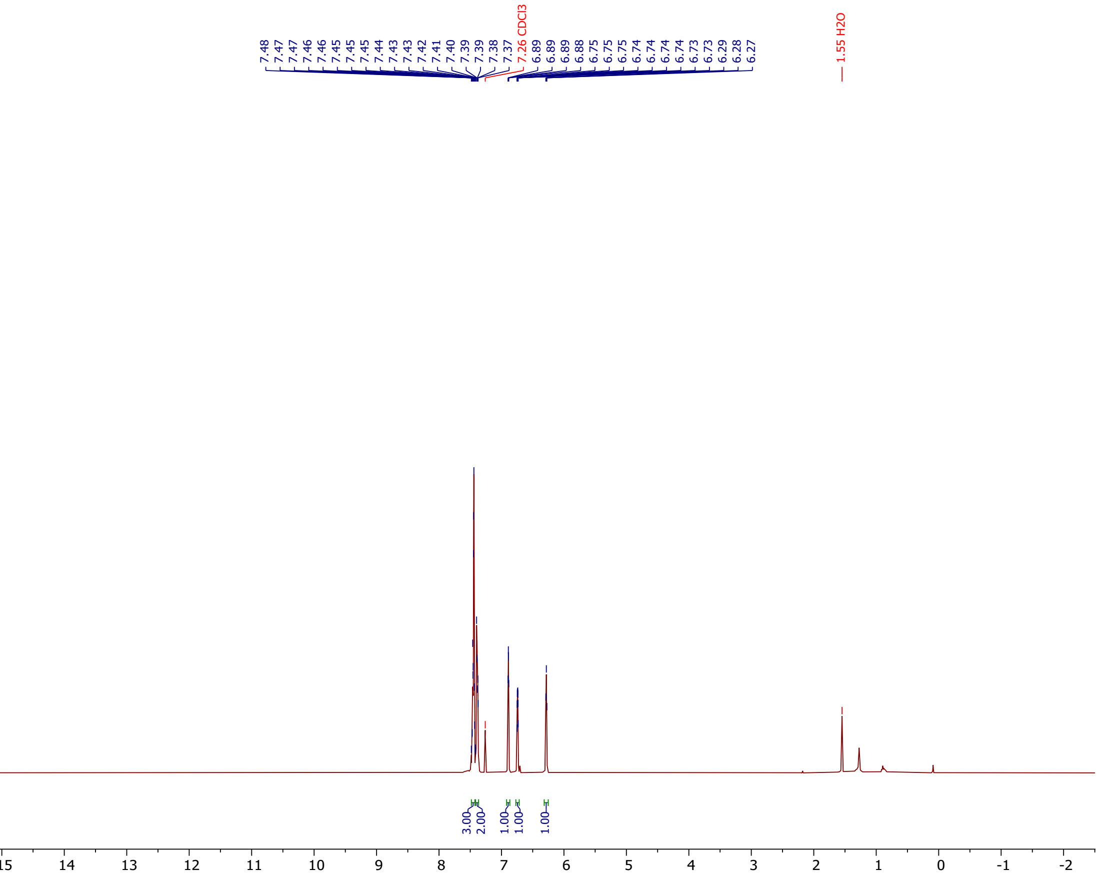

^{13}C NMR spectrum of **3k** ($CDCl_3$, 101 MHz)

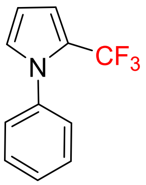

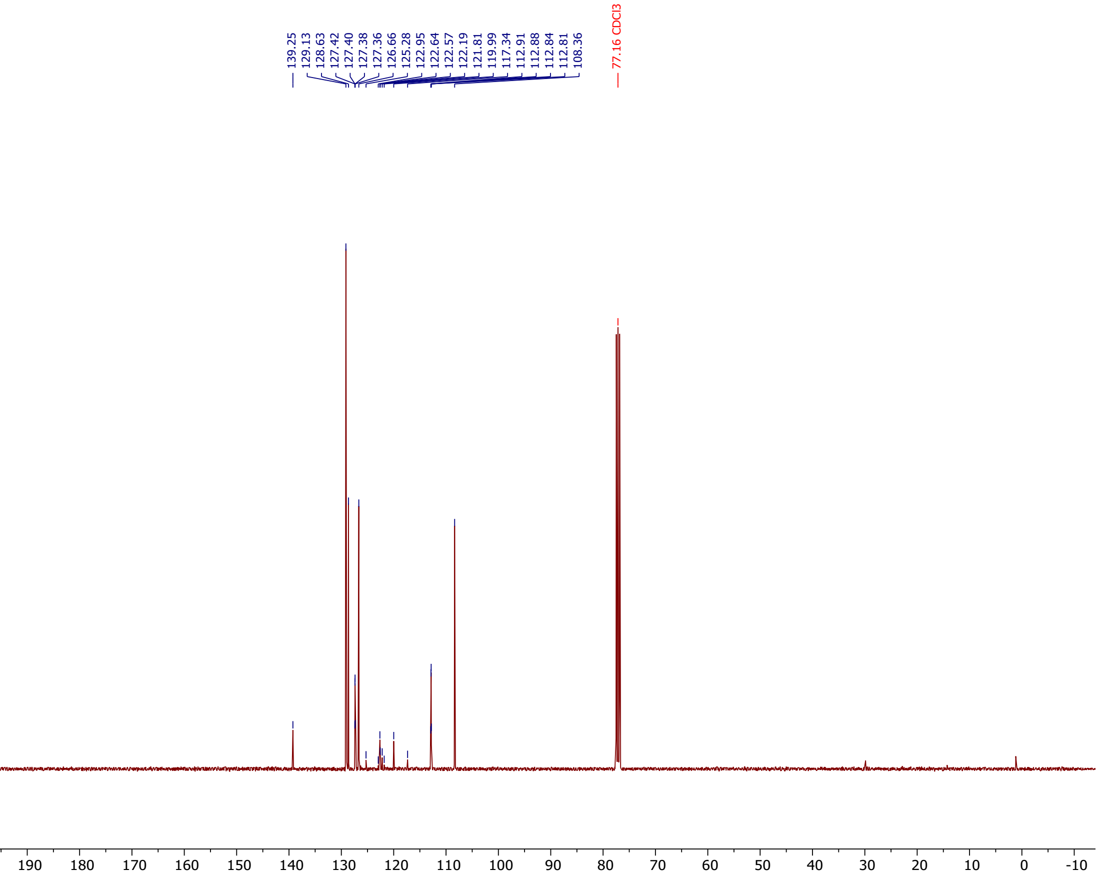

^{19}F NMR spectrum of **3k** ($CDCl_3$, 376 MHz)

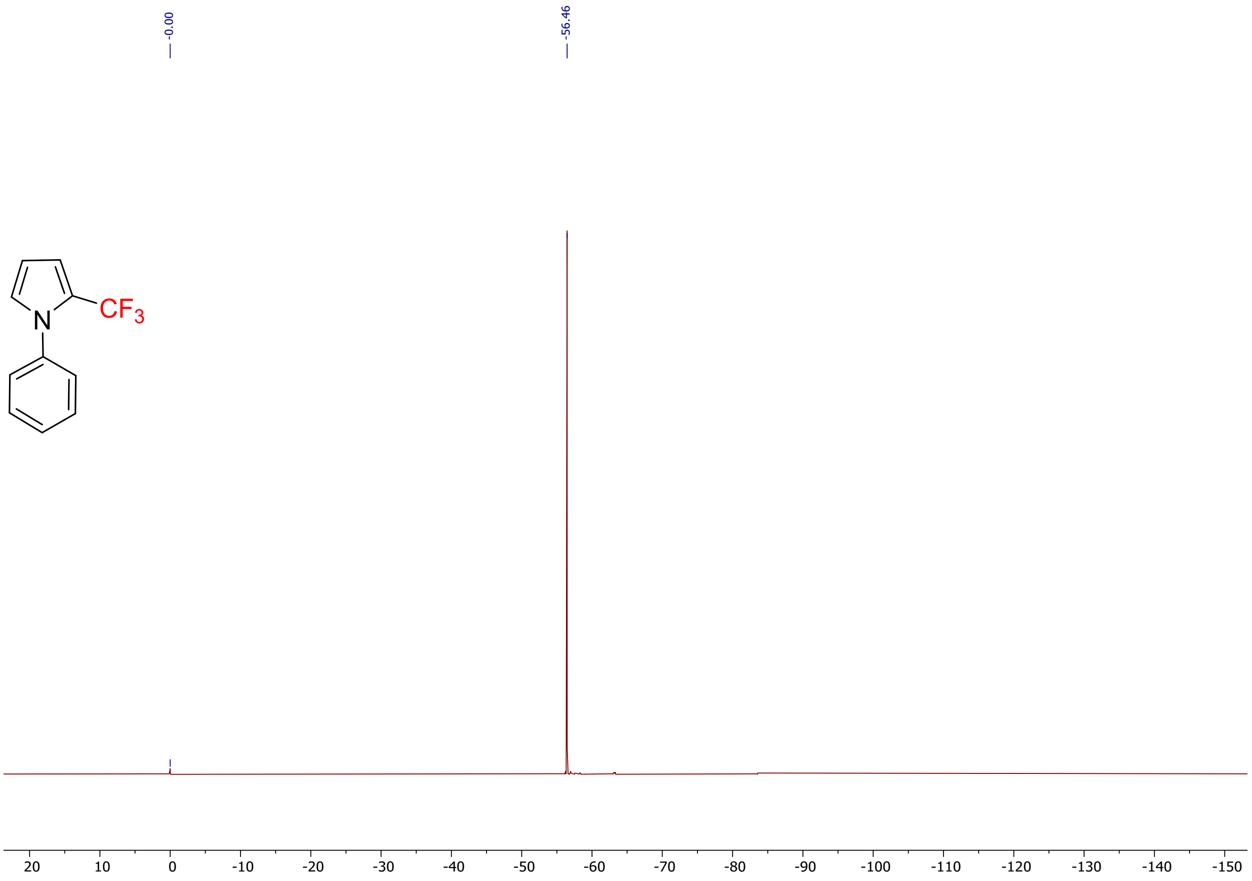

^{1}H NMR spectrum of **3l** ($CDCl_3$, 400 MHz)

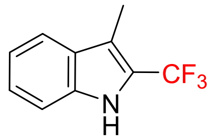

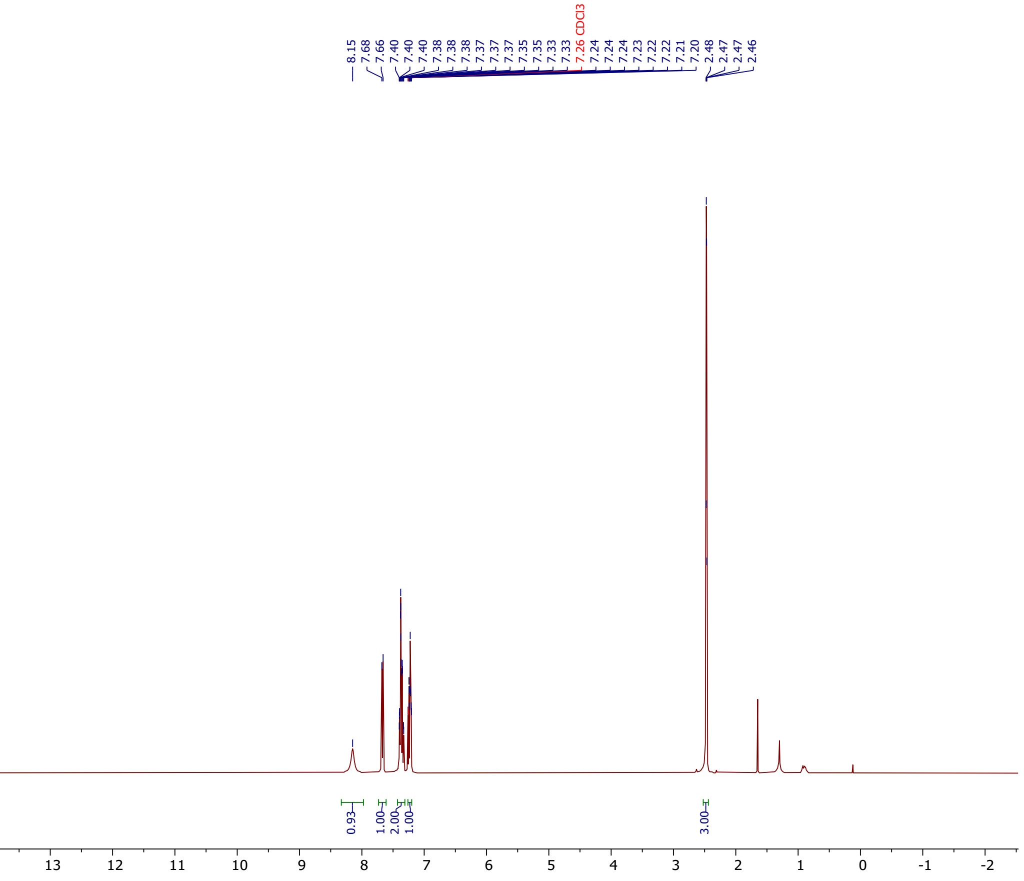

^{13}C NMR spectrum of **3l** ($CDCl_3$, 101 MHz)

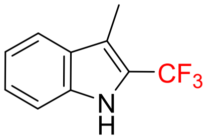

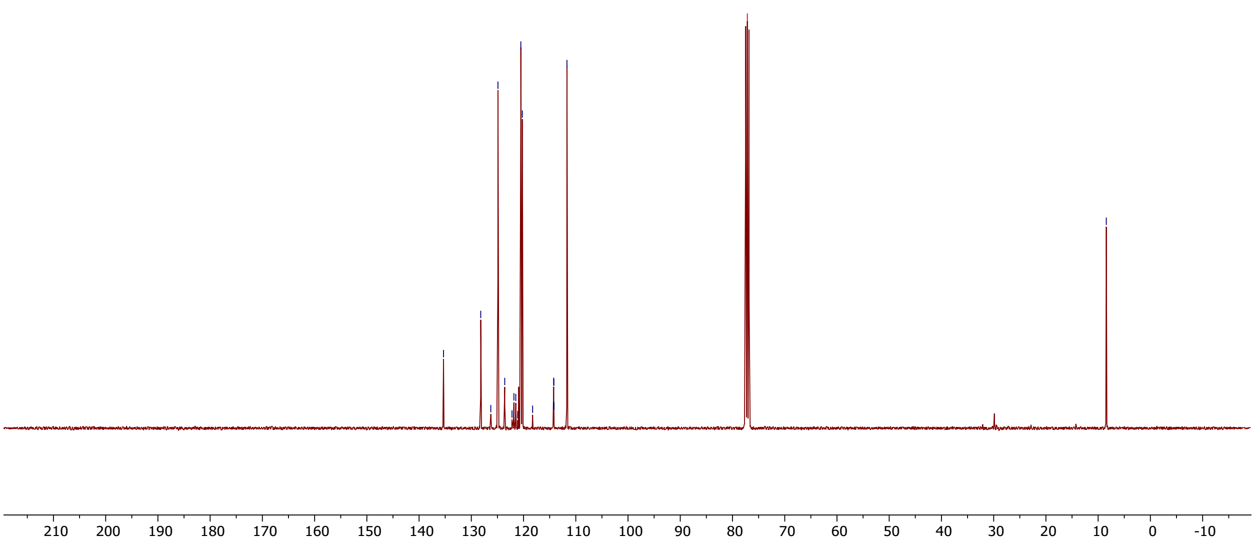

^{19}F NMR spectrum of **3l** ($CDCl_3$, 376 MHz)

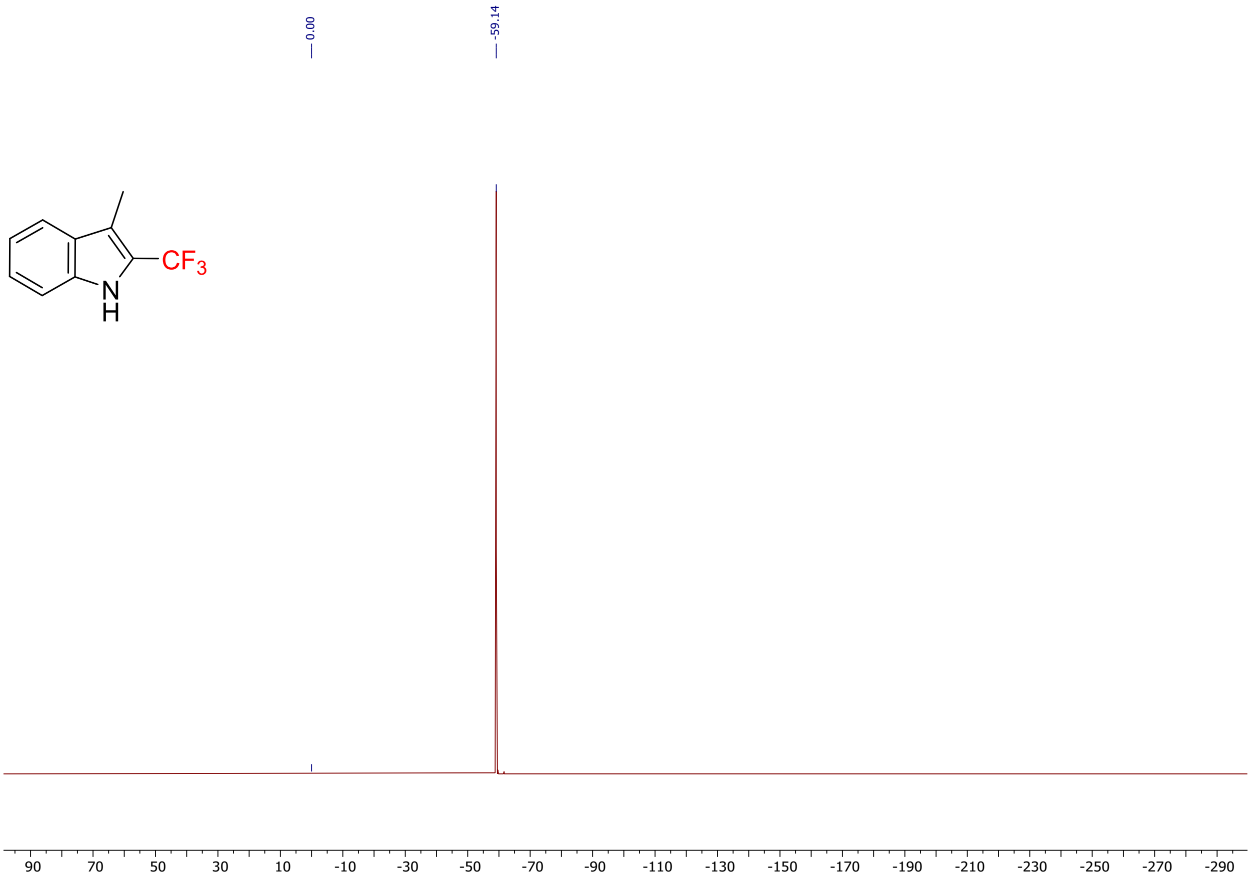

^{1}H NMR spectrum of **3m** ($CDCl_3$, 400 MHz)

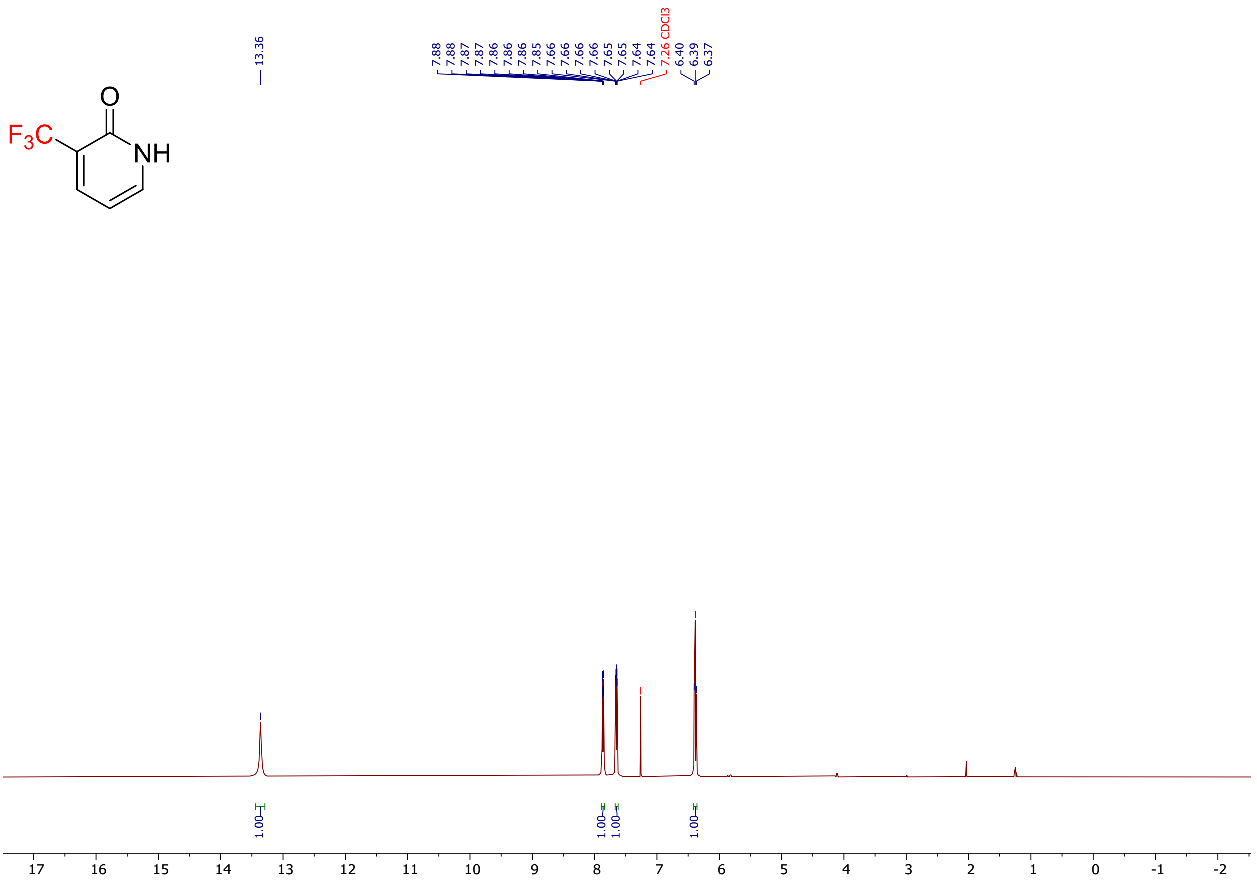

^{13}C NMR spectrum of **3m** ($CDCl_3$, 101 MHz)

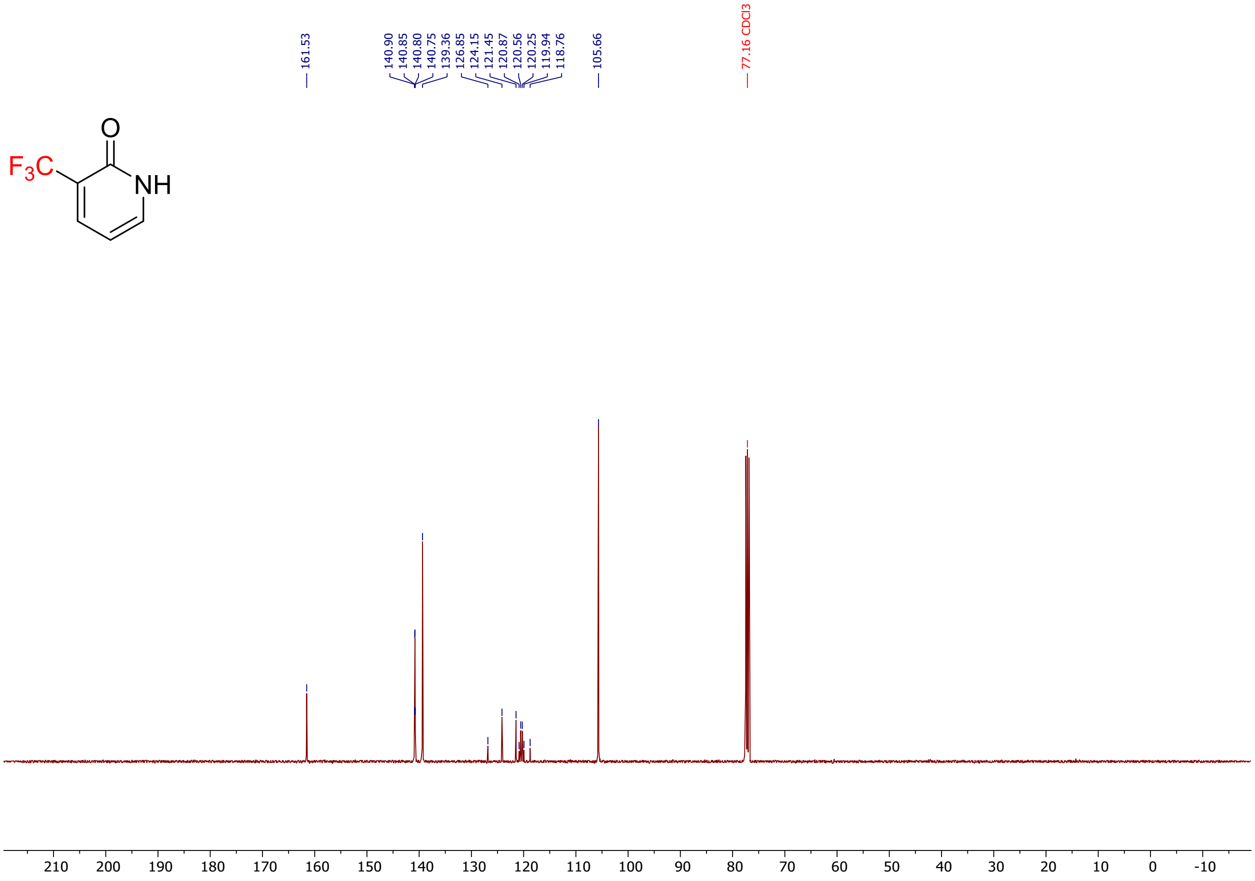

^{19}F NMR spectrum of **3m** ($CDCl_3$, 376 MHz)

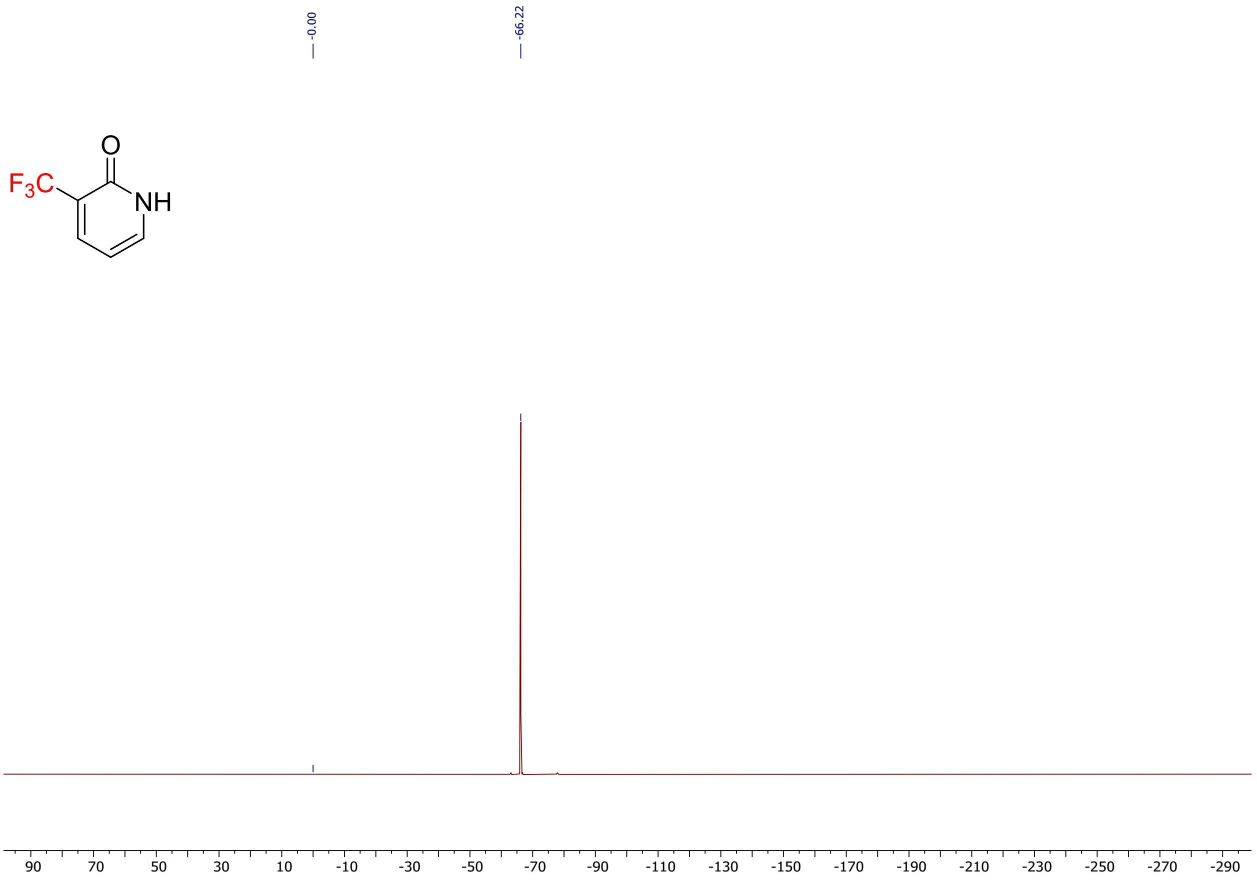

^{1}H NMR spectrum of **3n** ($CDCl_3$, 400 MHz)

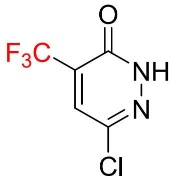

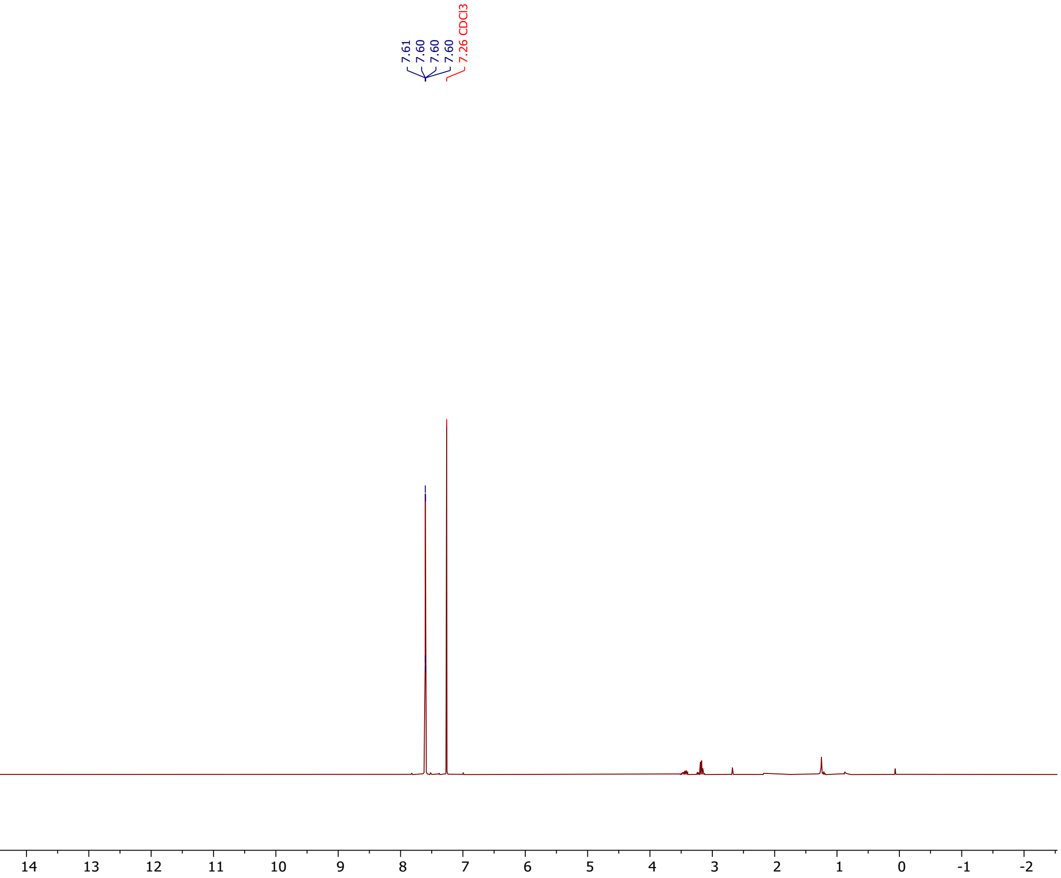

^{13}C NMR spectrum of **3n** ($CDCl_3$, 101 MHz)

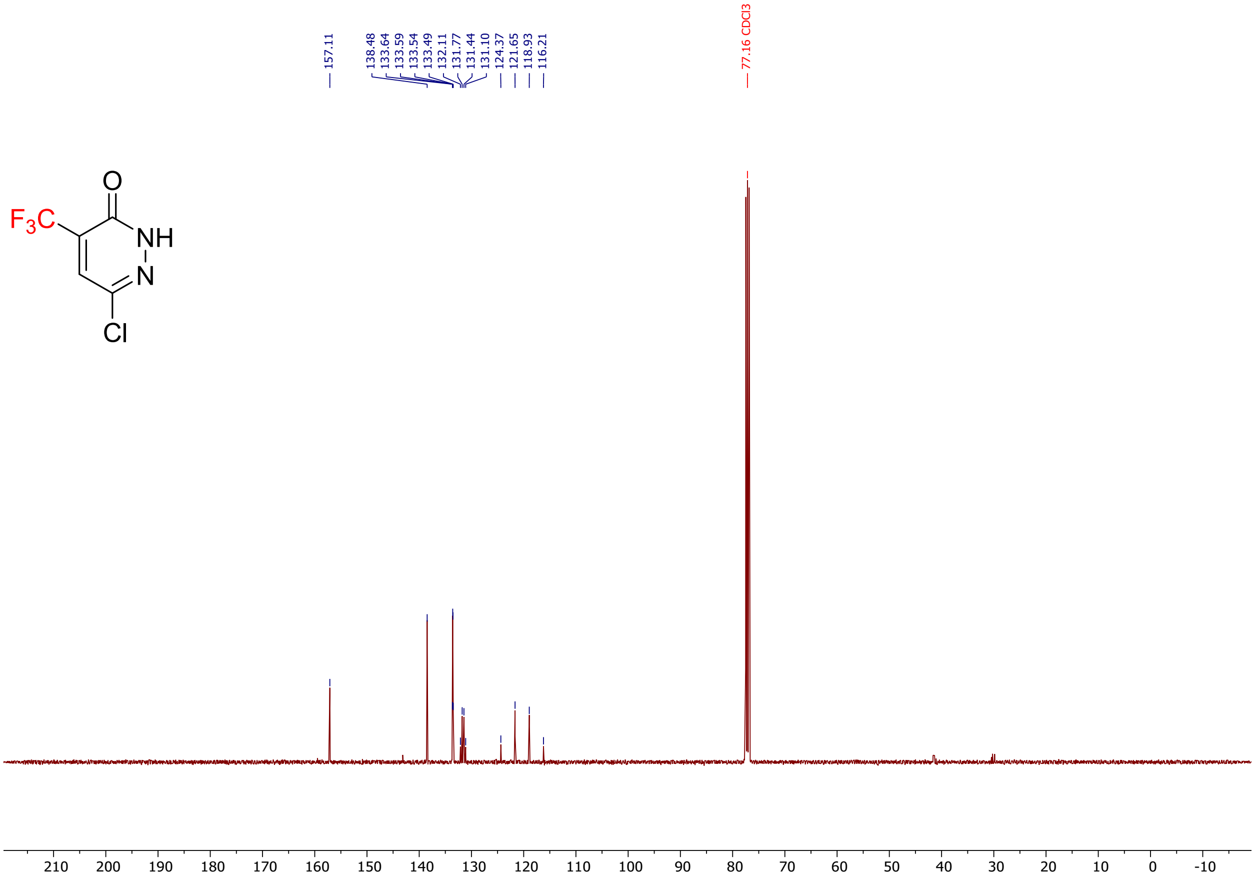

^{19}F NMR spectrum of **3n** ($CDCl_3$, 376 MHz)

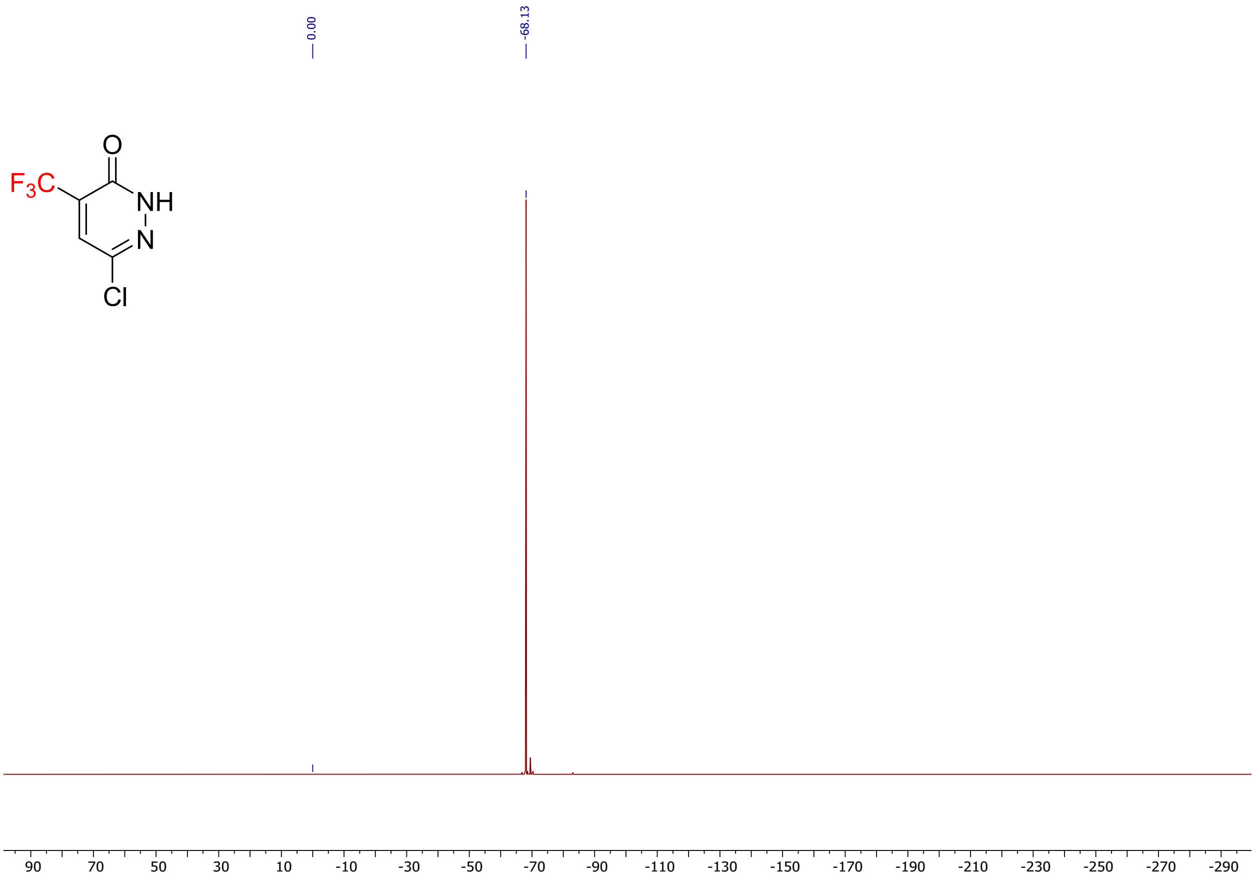

^{19}F NMR spectrum of crude **3o** ($CDCl_3$, 376 MHz)

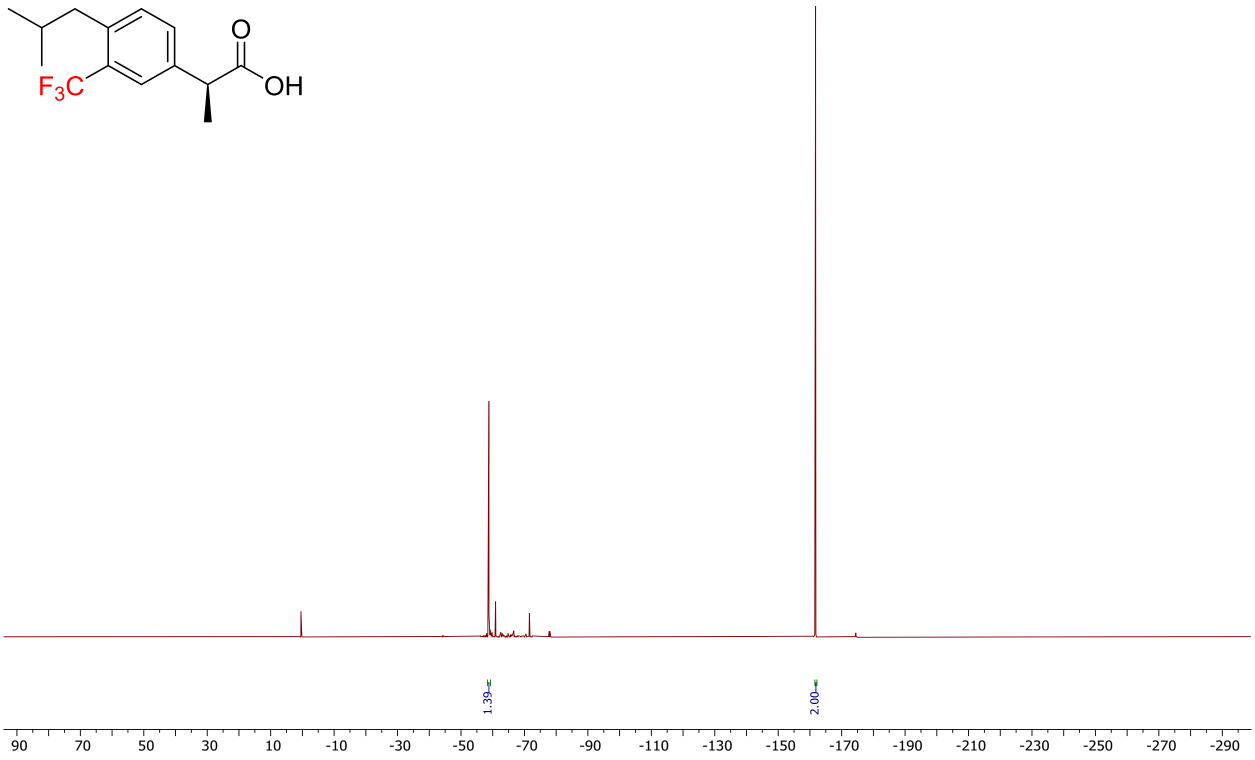

^{1}H NMR spectrum of **3p** ($CDCl_3$, 400 MHz)

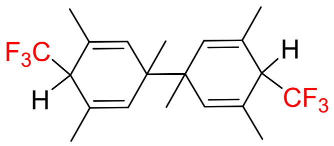

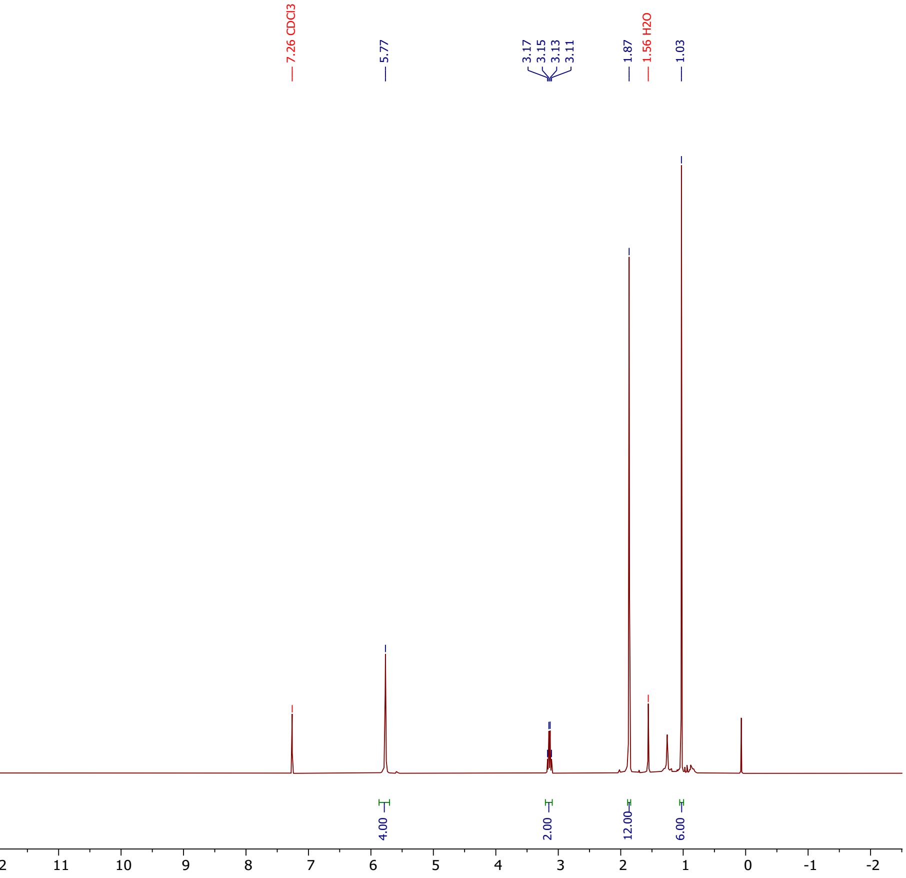

^{13}C NMR spectrum of **3p** ($CDCl_3$, 101 MHz)

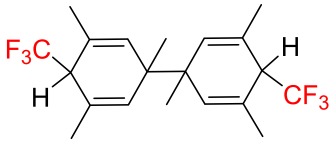

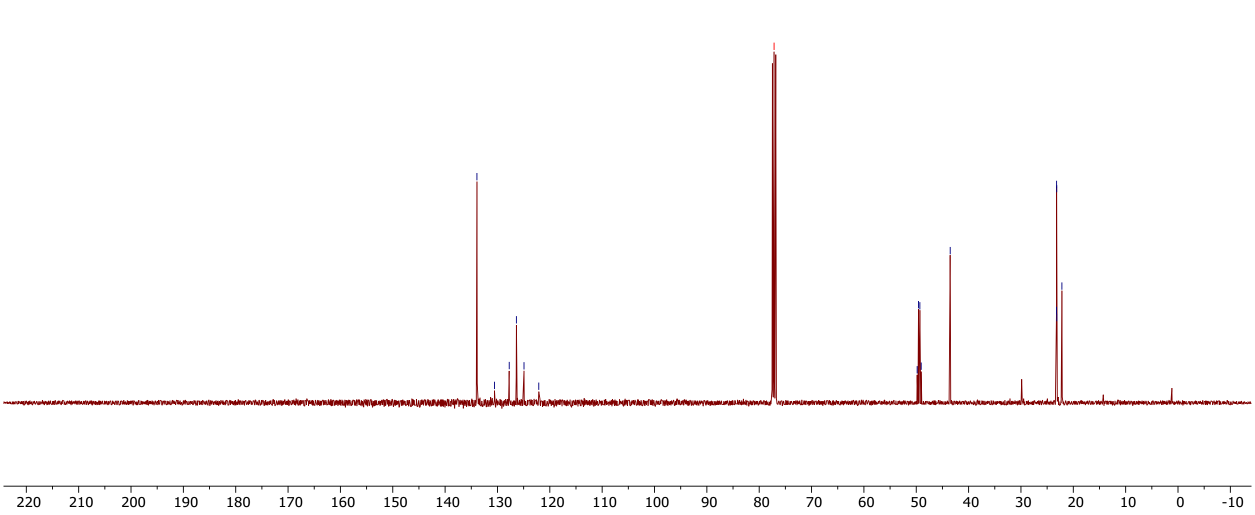

^{19}F NMR spectrum of **3p** ($CDCl_3$, 376 MHz)

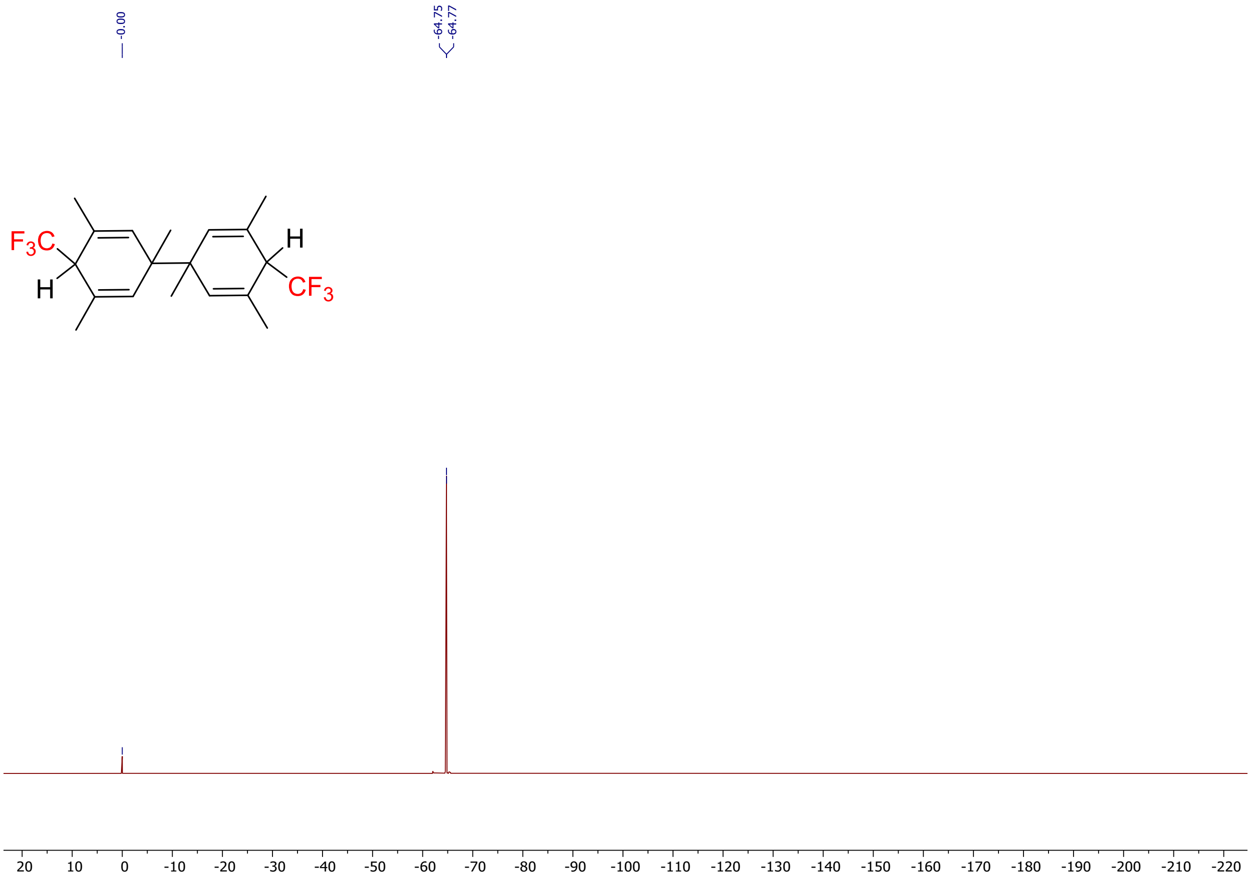

^{1}H NMR spectrum of **3q** ($CDCl_3$, 400 MHz)

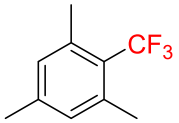

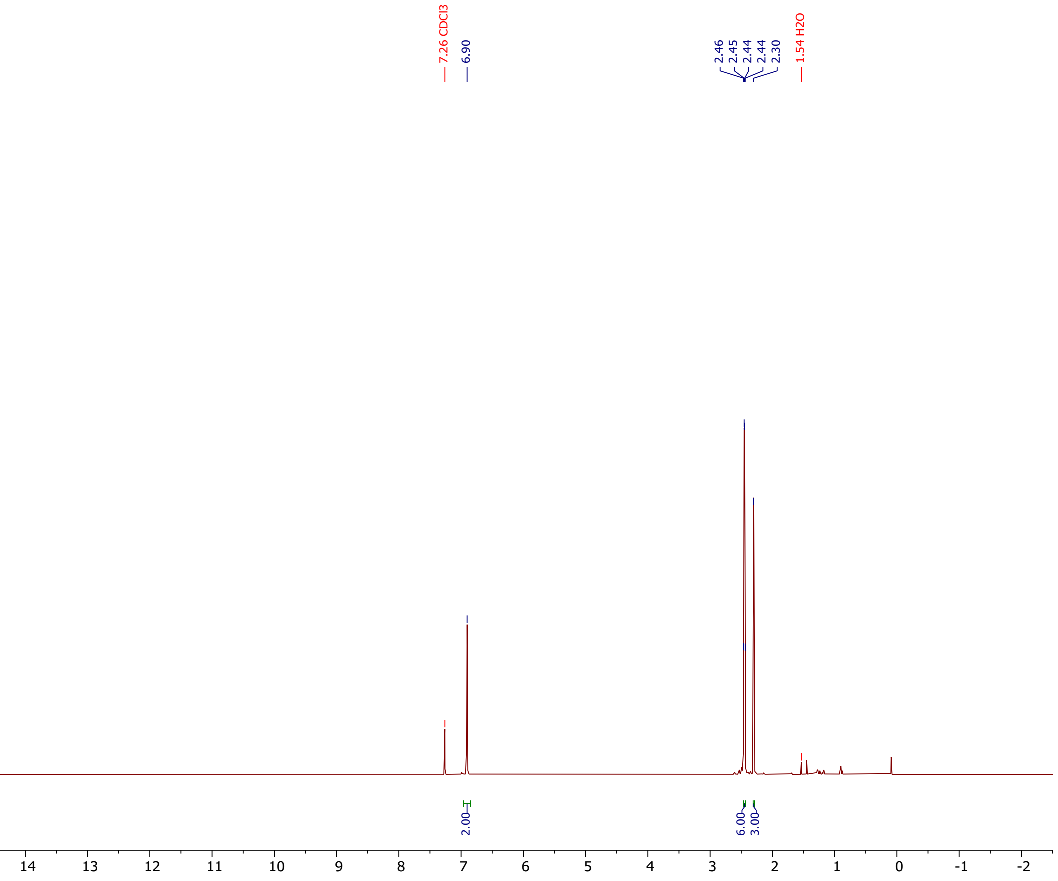

^{13}C NMR spectrum of **3q** ($CDCl_3$, 101 MHz)

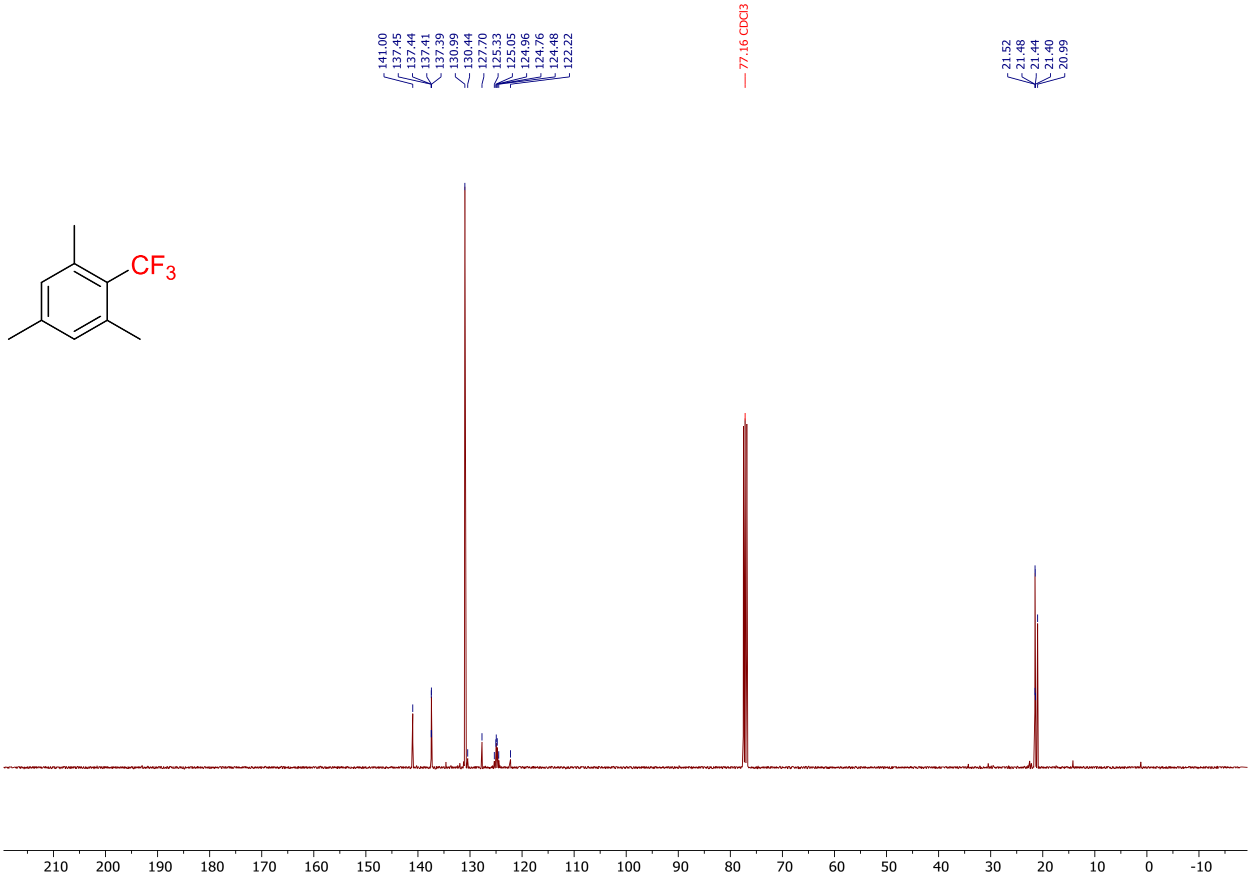

^{19}F NMR spectrum of **3q** ($CDCl_3$, 376 MHz)

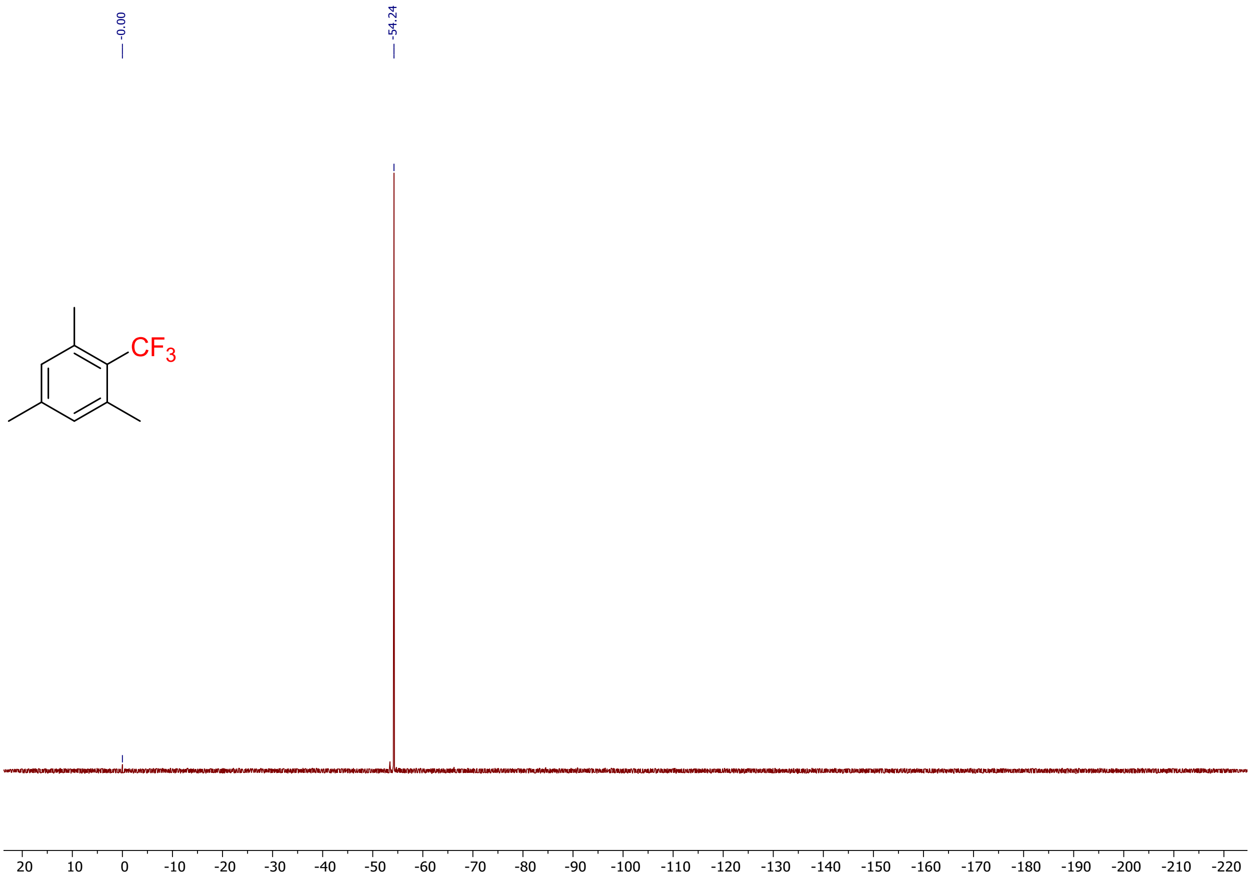

^{1}H NMR spectrum of **4** (MeOD, 400 MHz)

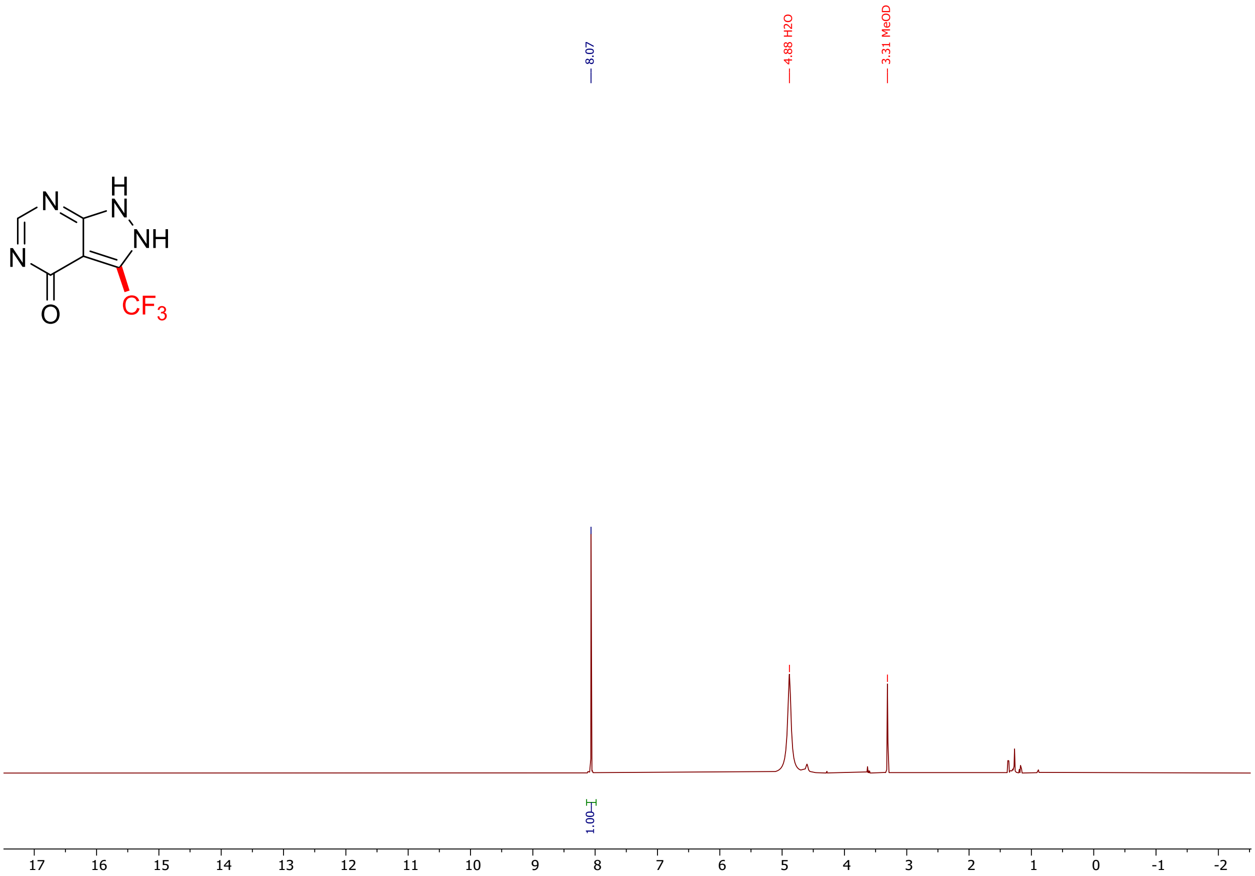

^{13}C NMR spectrum of **4** (MeOD, 101 MHz)

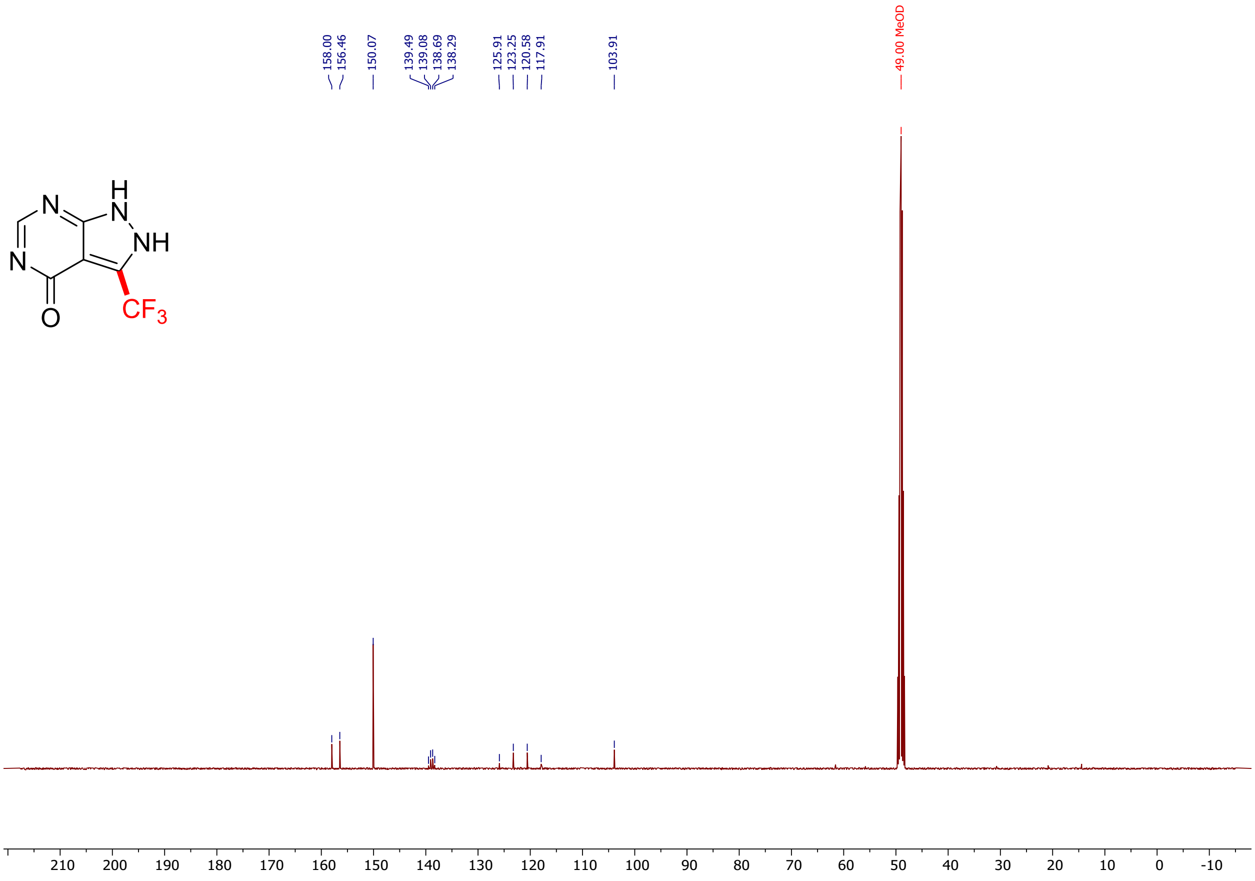

^{19}F NMR spectrum of **4** (MeOD, 376 MHz)

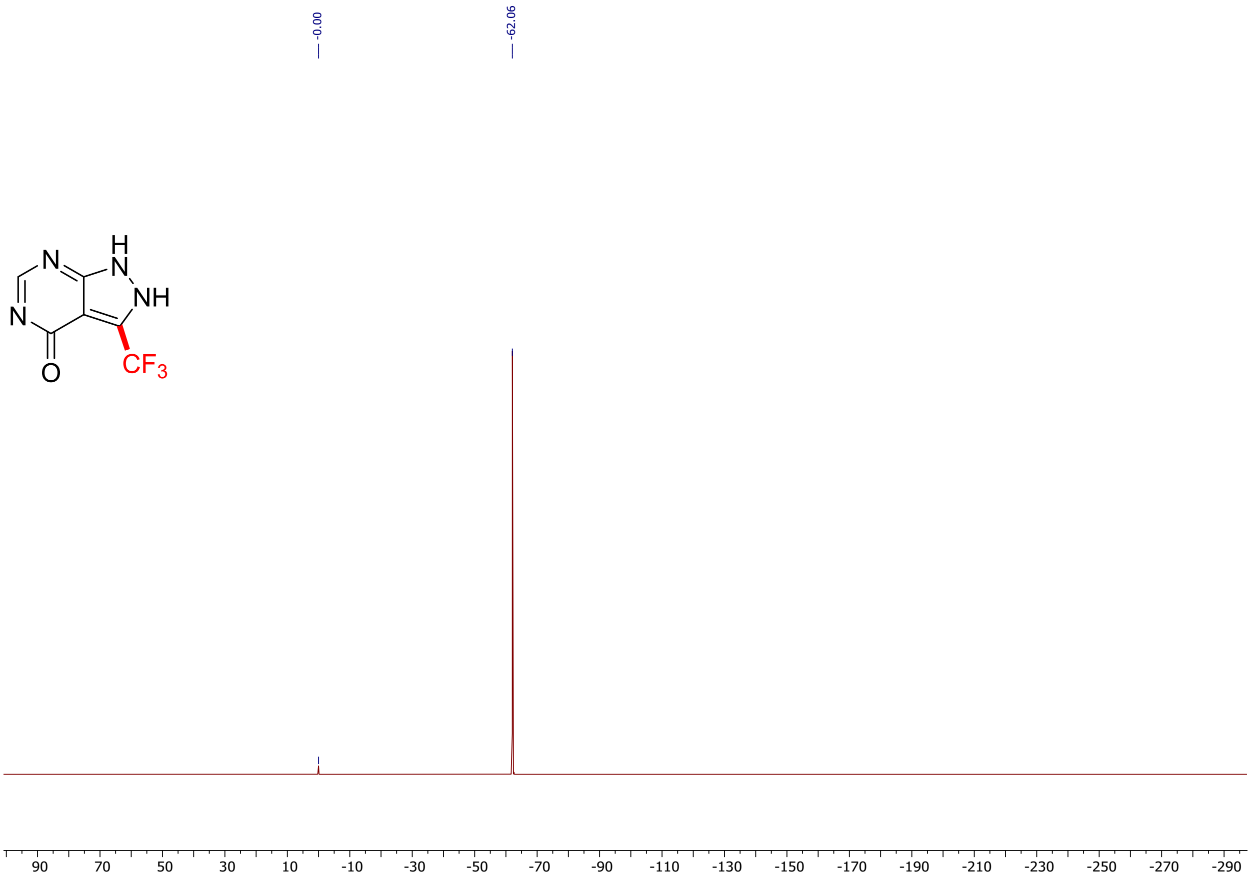

^{1}H NMR spectrum of **5** ($CDCl_3$, 400 MHz)

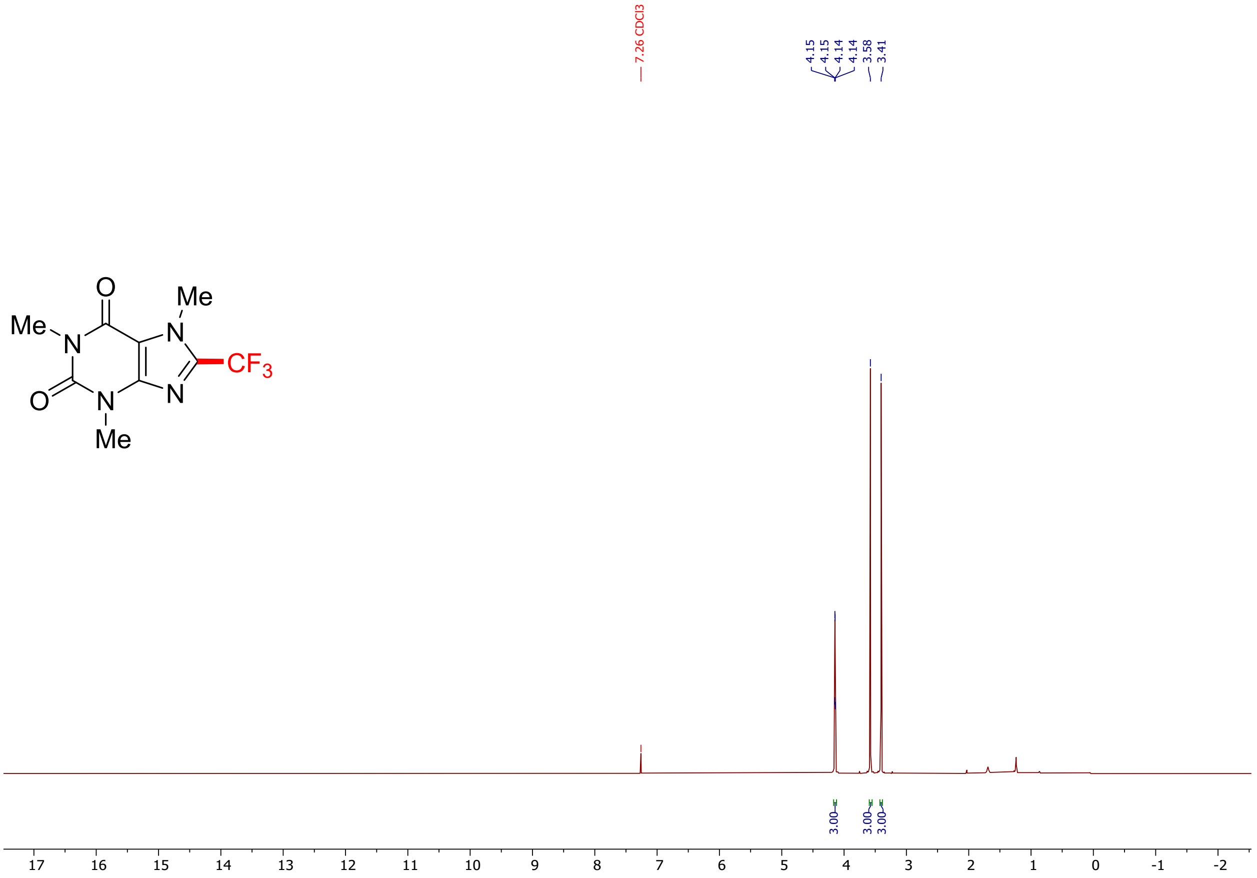

^{13}C NMR spectrum of **5** ($CDCl_3$, 101 MHz)

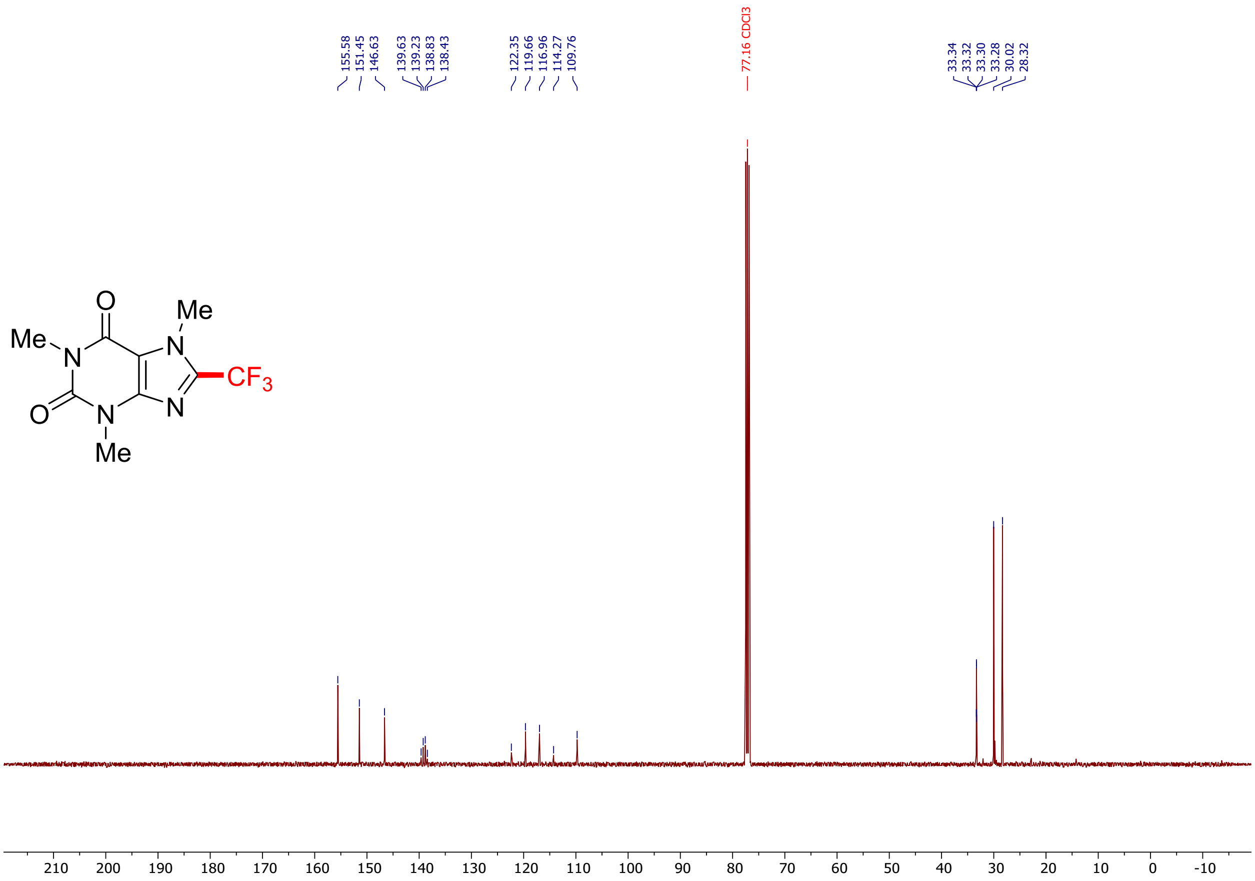

^{19}F NMR spectrum of **5** ($CDCl_3$, 376 MHz)

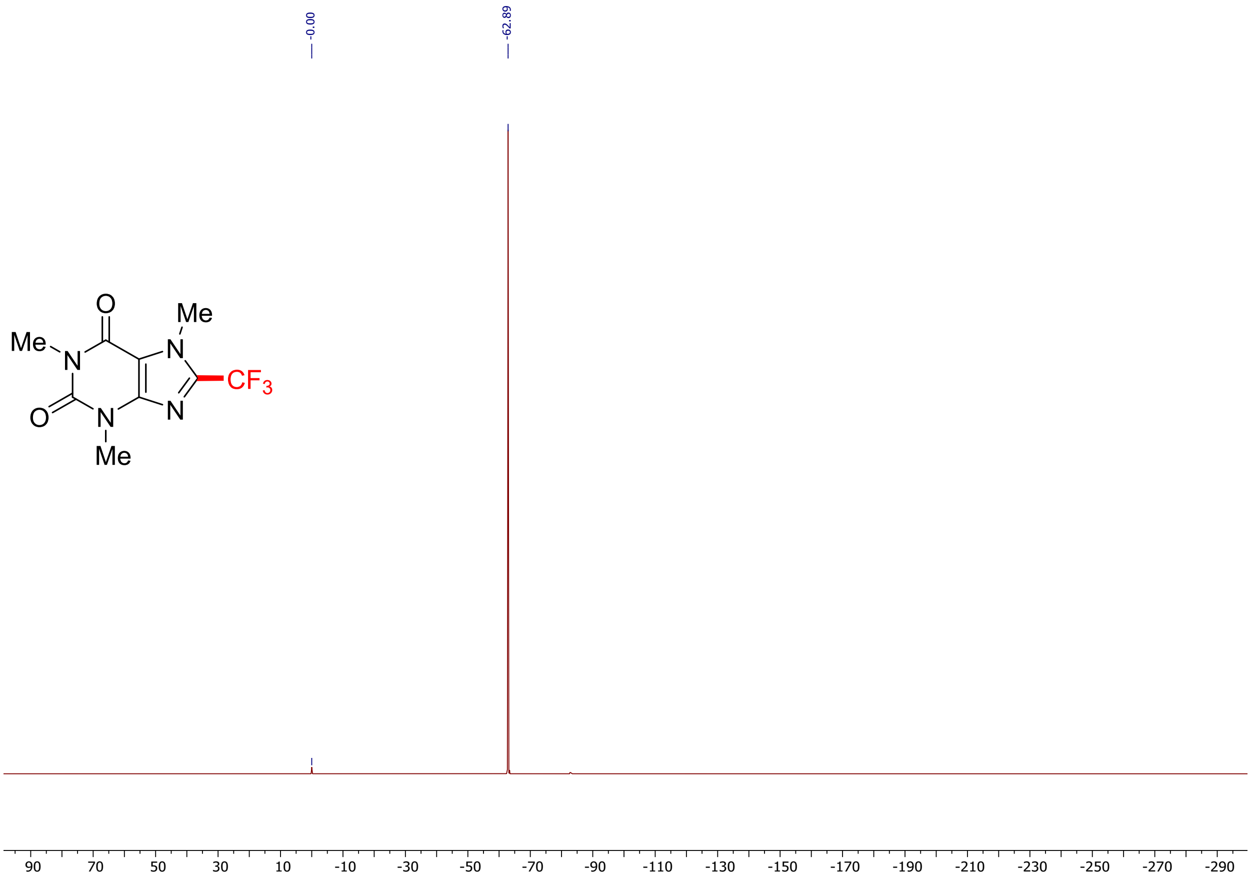

^{1}H NMR spectrum of **6** ($CDCl_3$, 400 MHz)

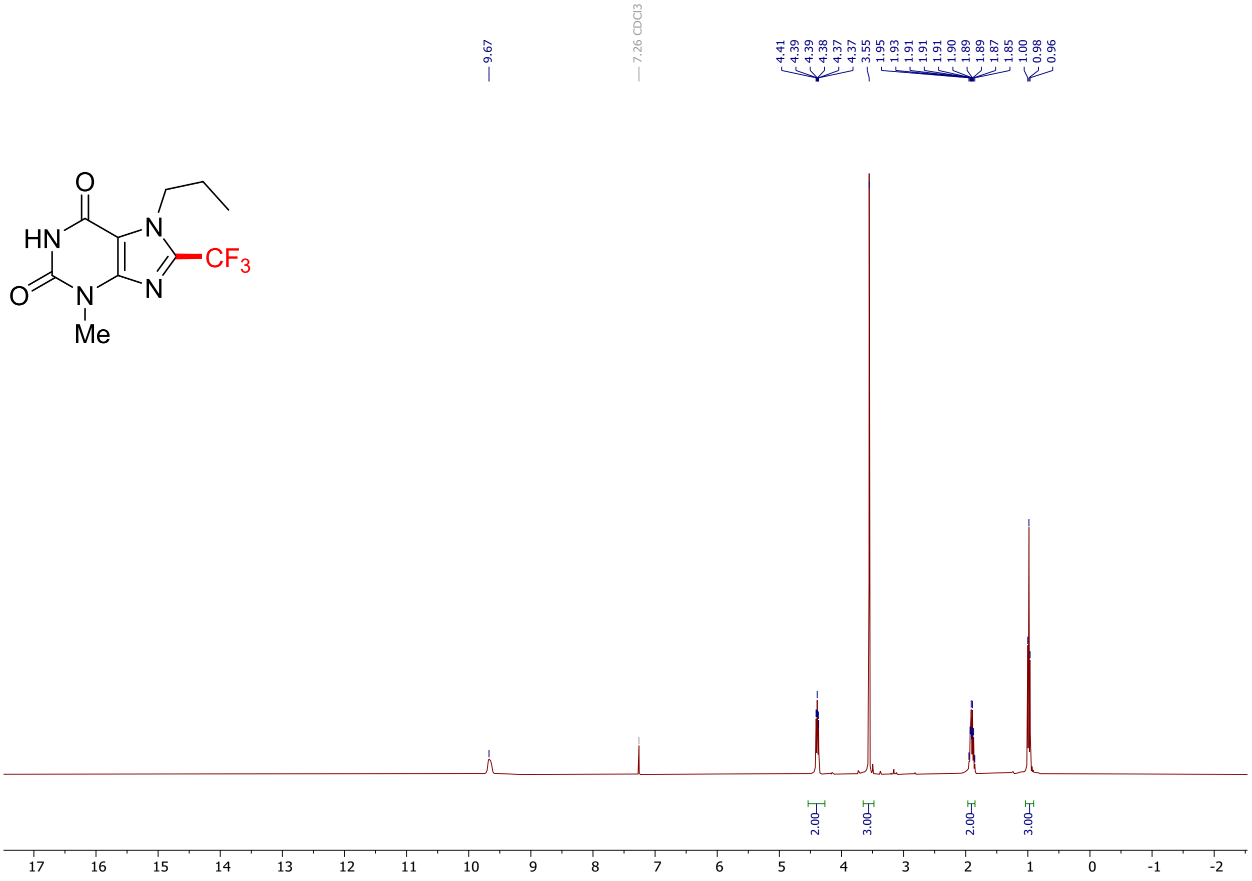

^{13}C NMR spectrum of **6** ($CDCl_3$, 101 MHz)

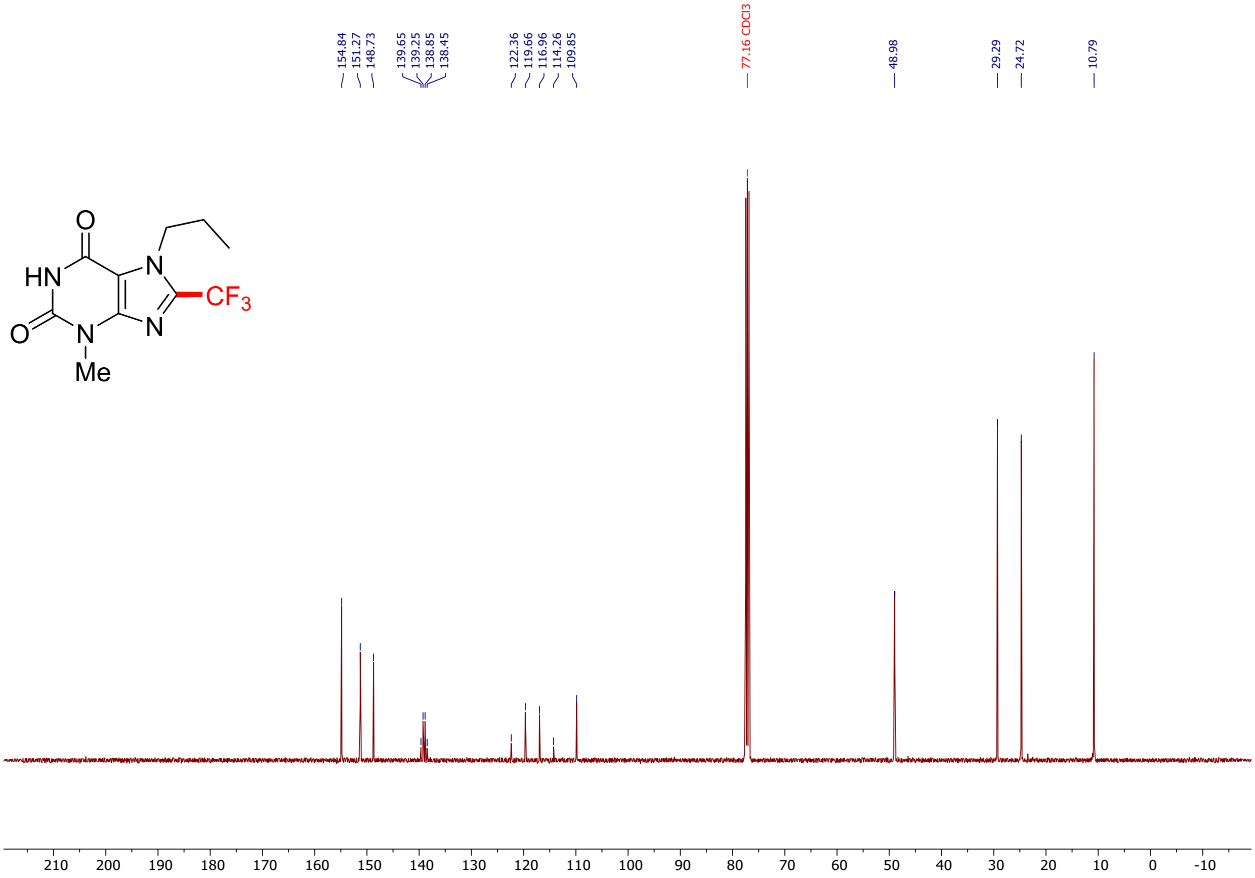

^{19}F NMR spectrum of **6** ($CDCl_3$, 376 MHz)

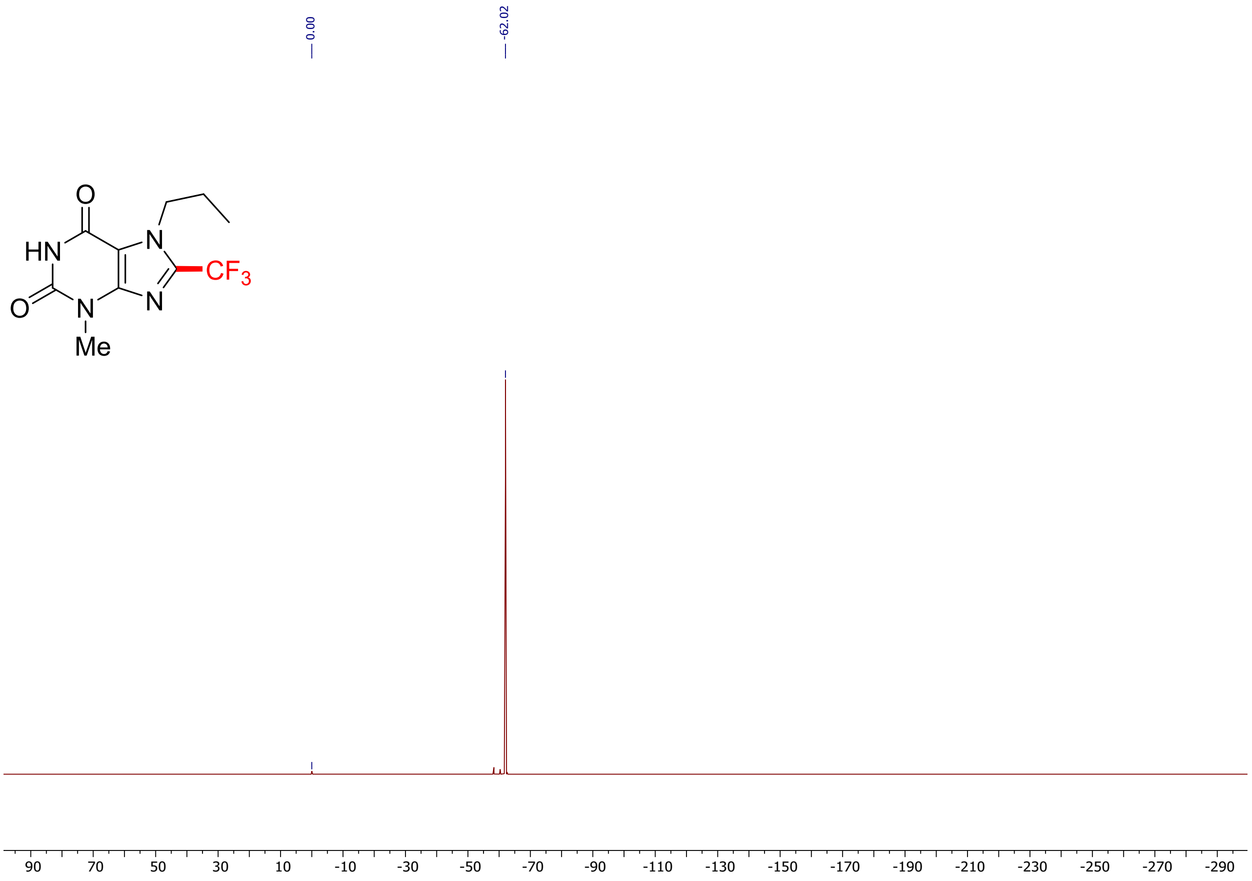

^{1}H NMR spectrum of **7** ($CDCl_3$, 400 MHz)

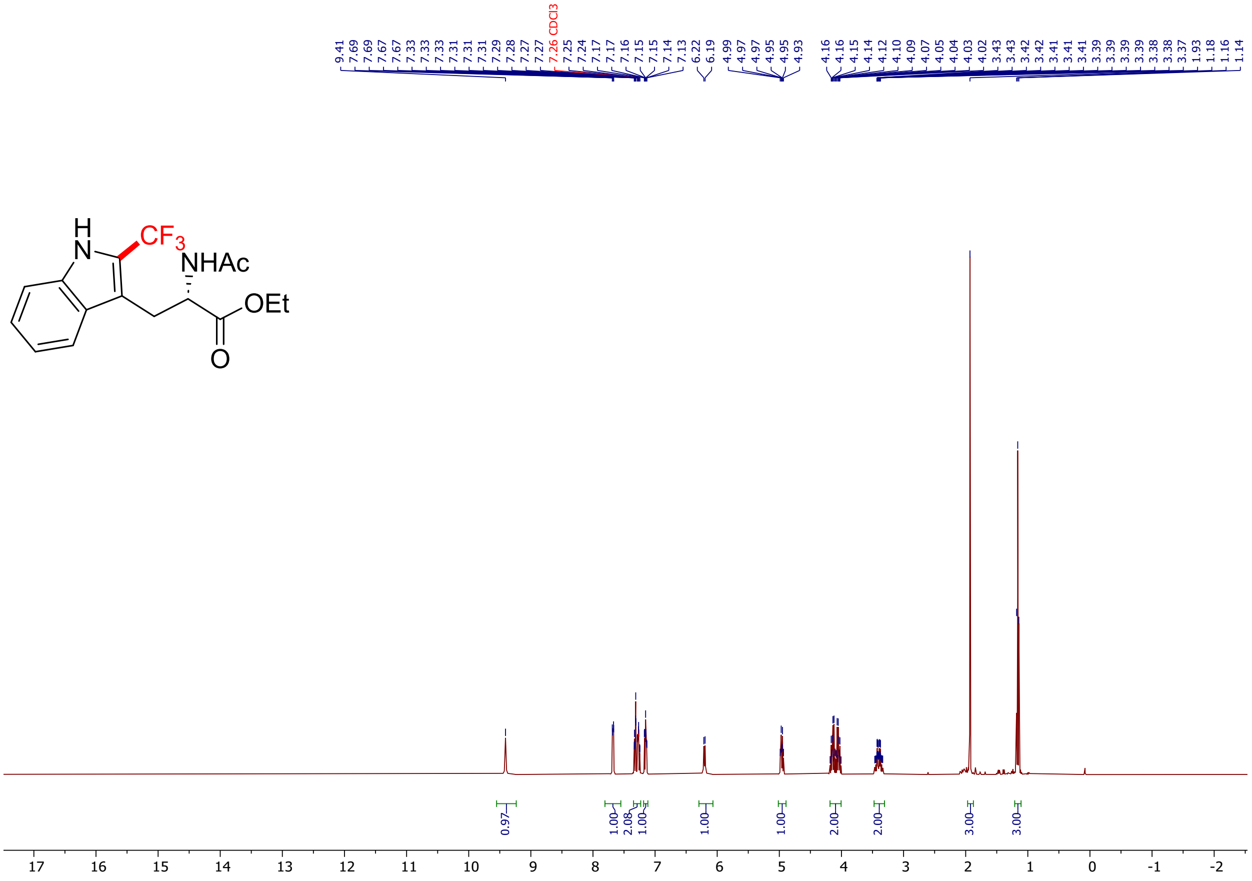

^{13}C NMR spectrum of **7** ($CDCl_3$, 101 MHz)

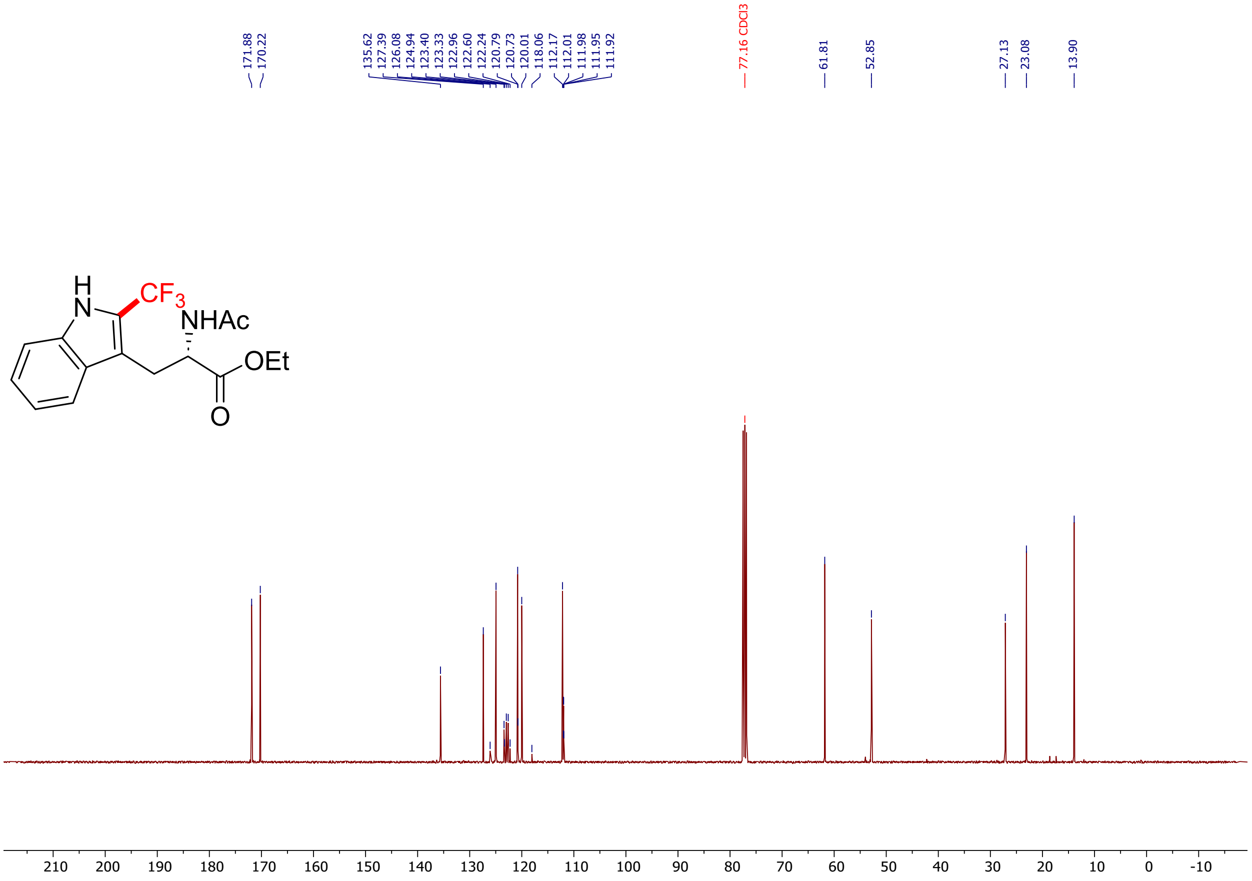

^{19}F NMR spectrum of **7** ($CDCl_3$, 376 MHz)

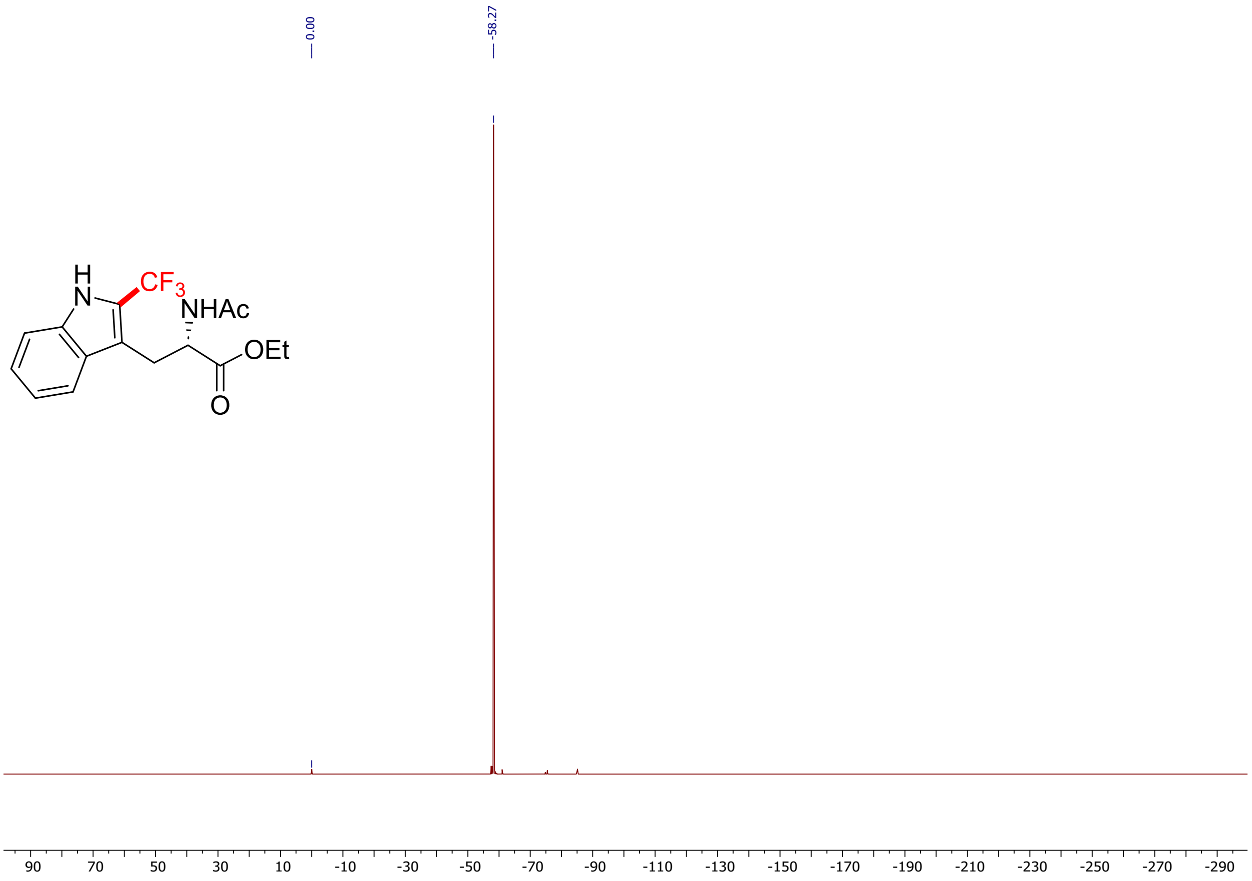

^{1}H NMR spectrum of **8** ($(CD_3)_2CO$, 400 MHz)

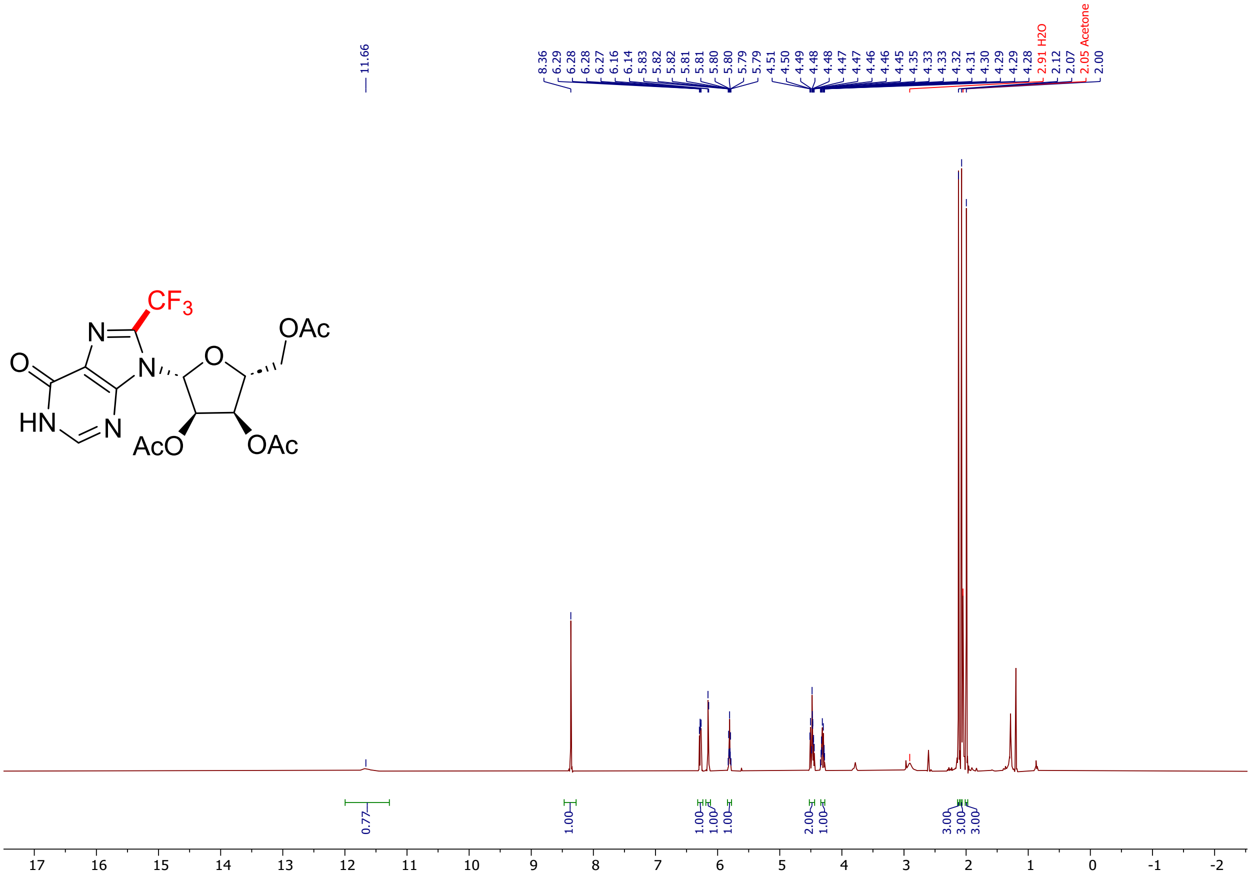

^{13}C NMR spectrum of **8** ($(CD_3)_2CO$, 101 MHz)

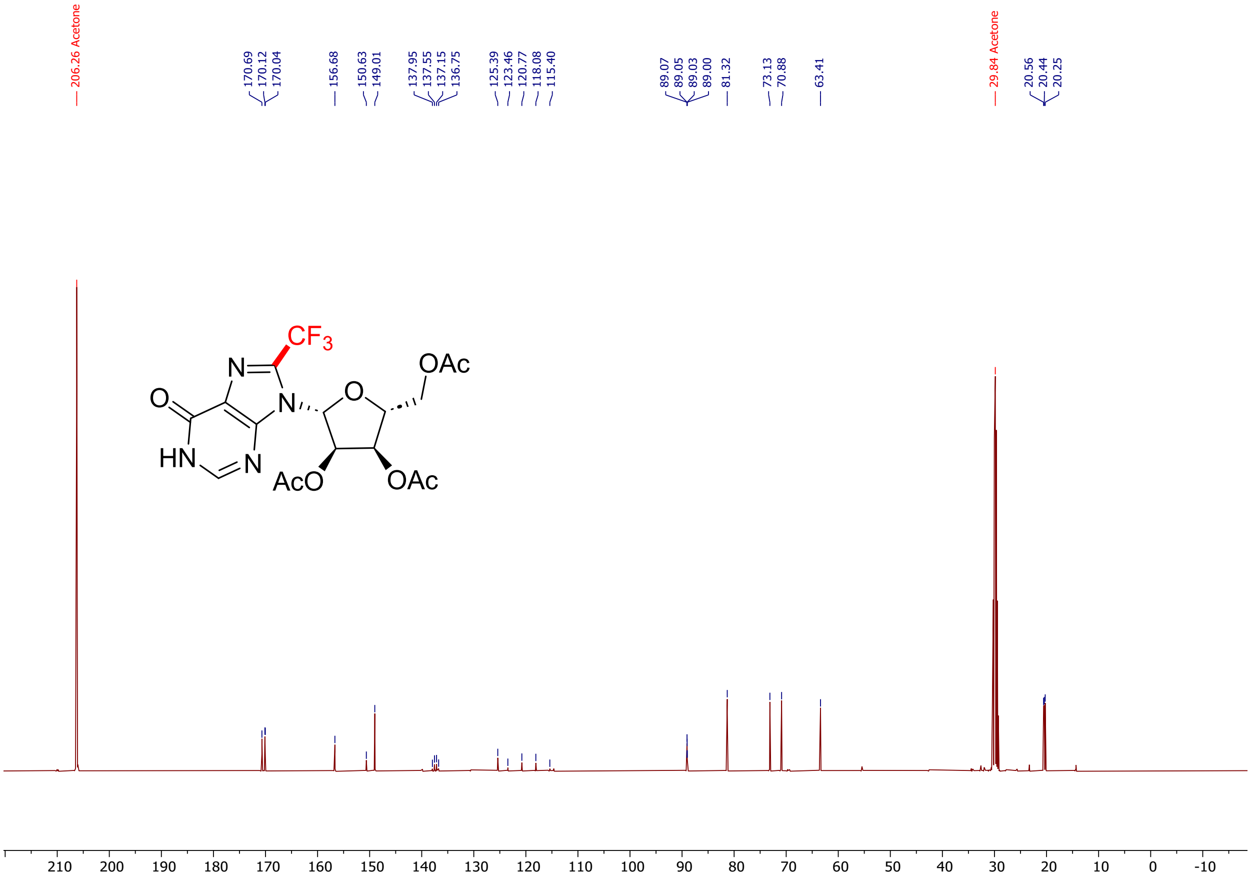

^{19}F NMR spectrum of **8** ($(CD_3)_2CO$, 376 MHz)

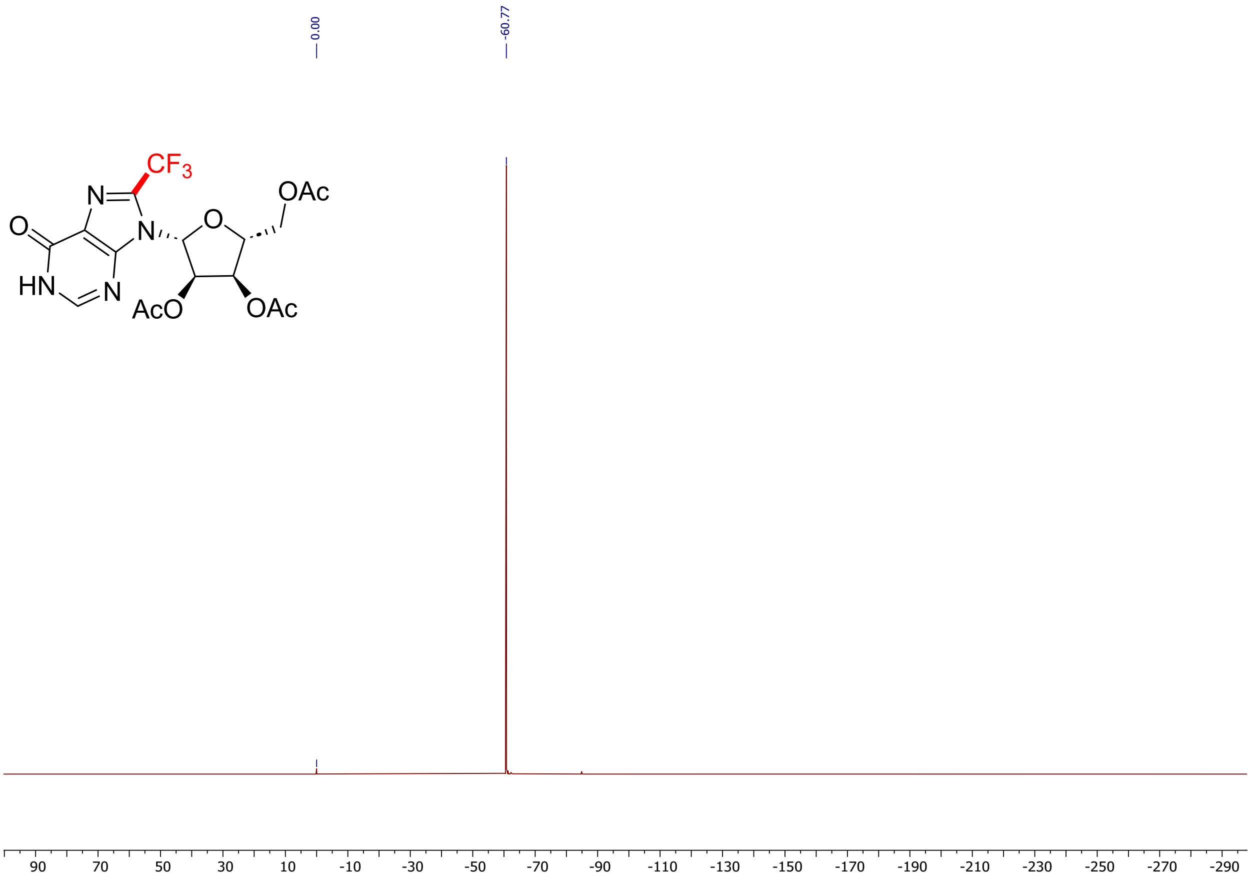

^{1}H NMR spectrum of **9** (DMSO-d_6, 400 MHz)

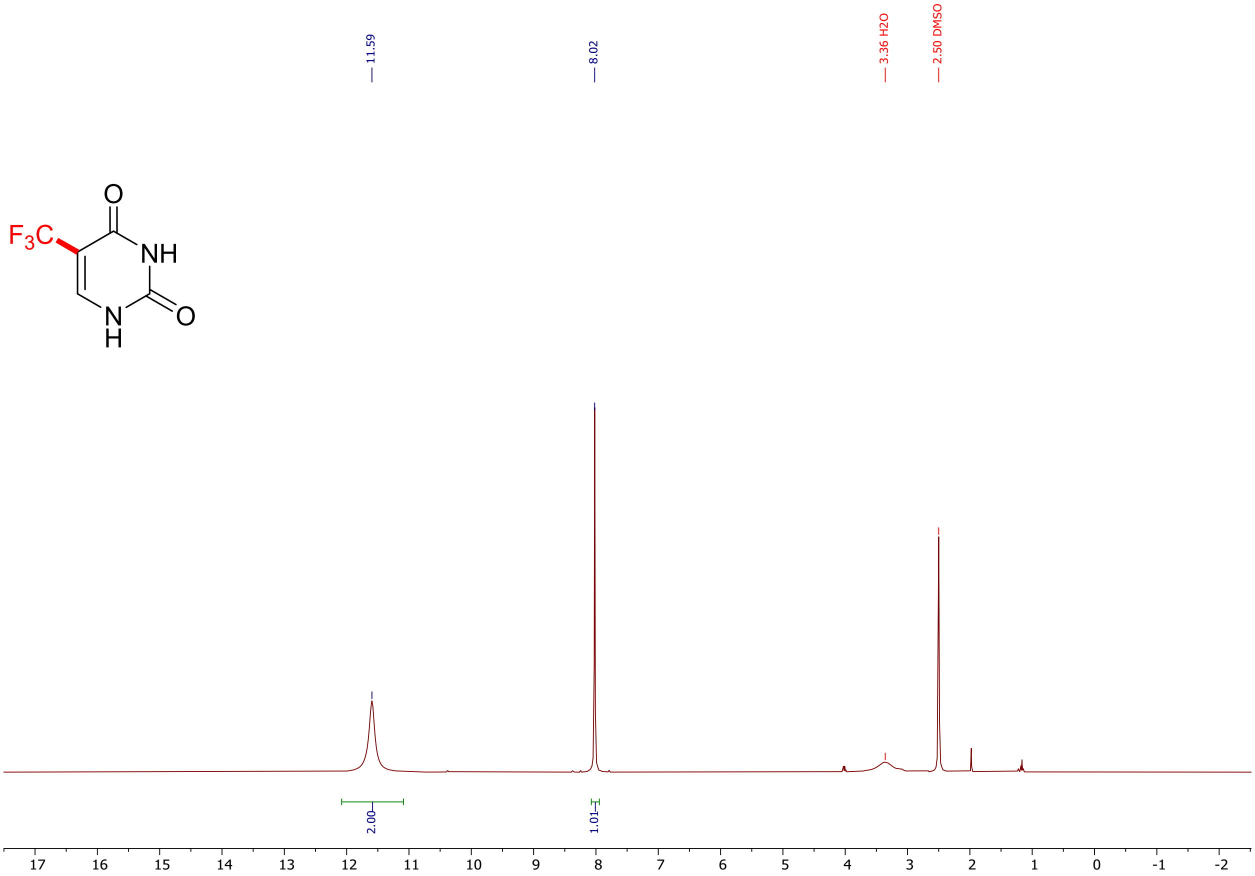

^{13}C NMR spectrum of **9** (DMSO-d_6, 101 MHz)

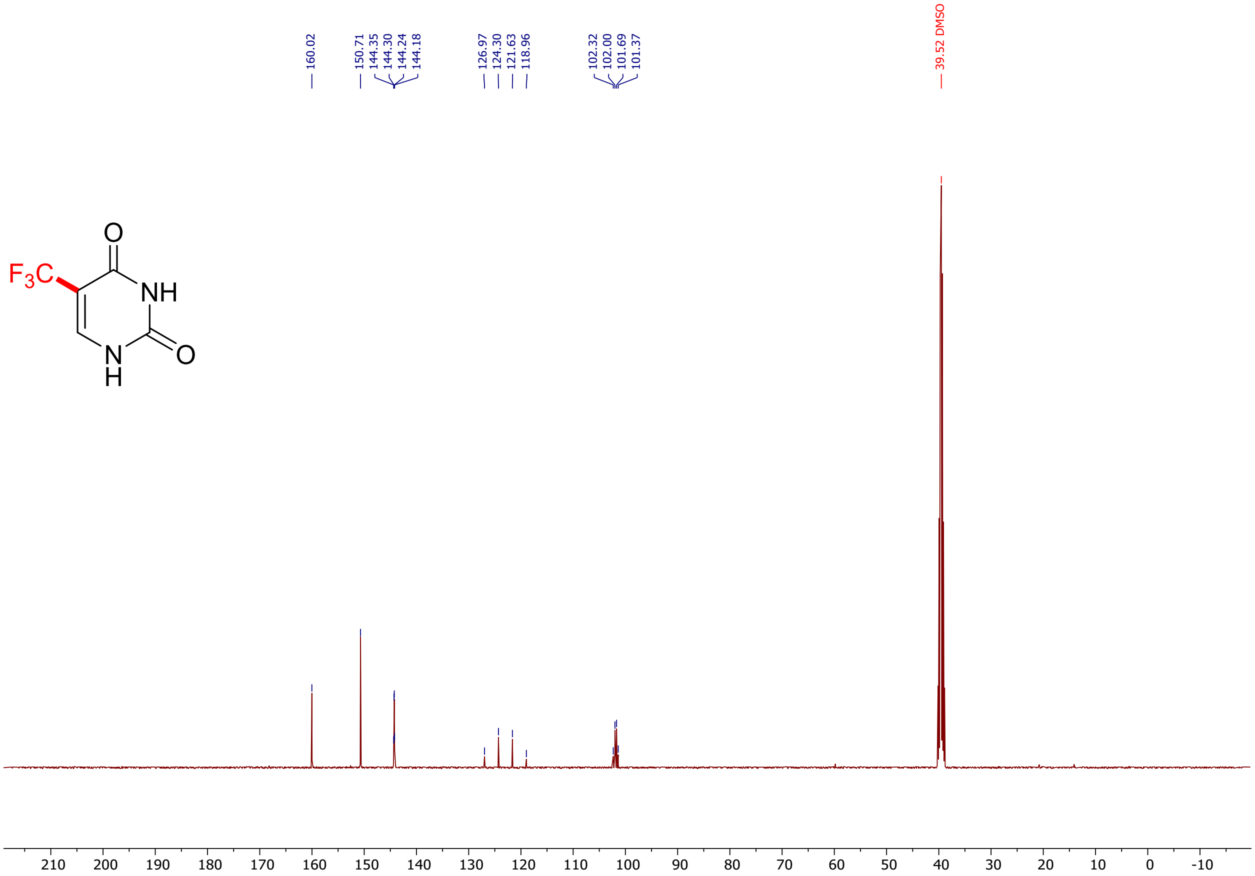

^{19}F NMR spectrum of **9** (DMSO-d_6, 376 MHz)

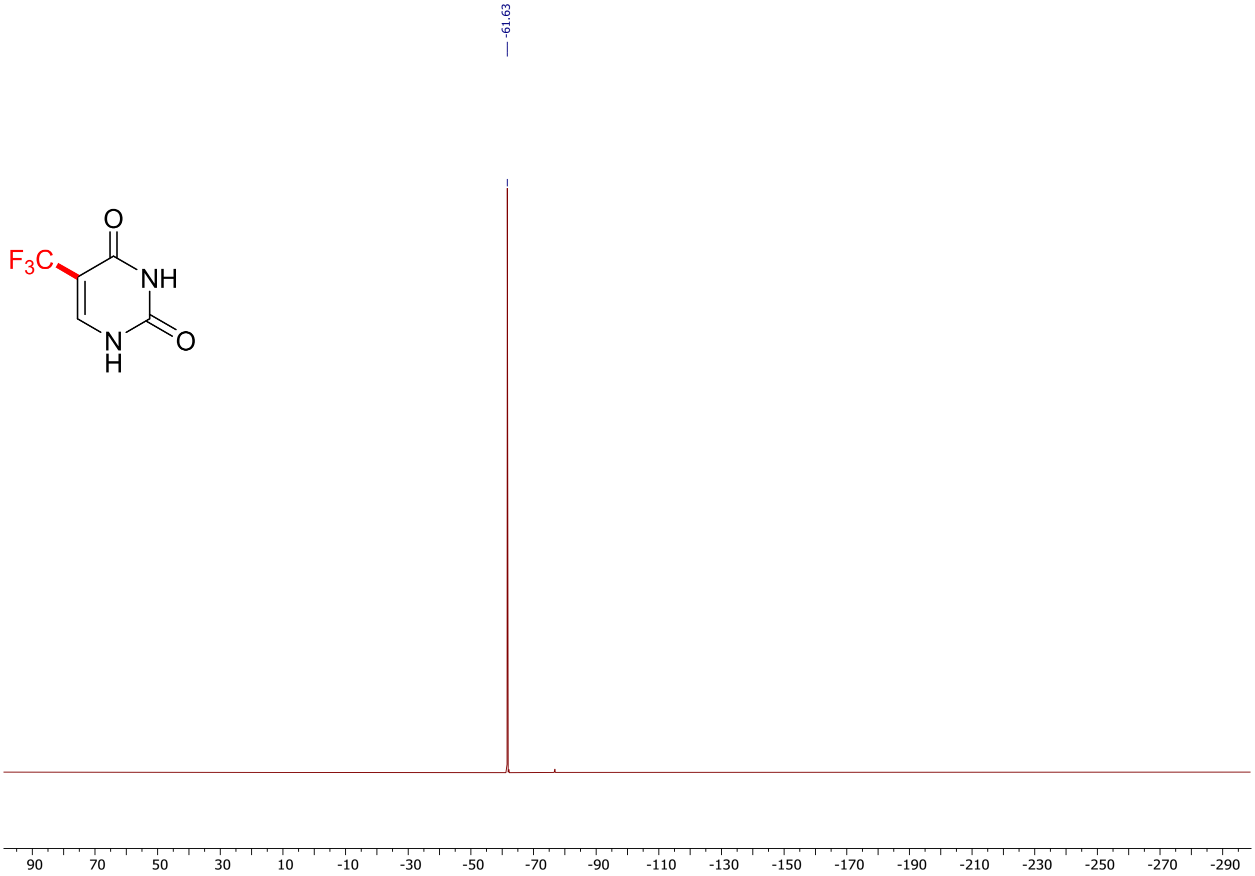

^{1}H NMR spectrum of **10** ($CDCl_3$, 300 MHz)

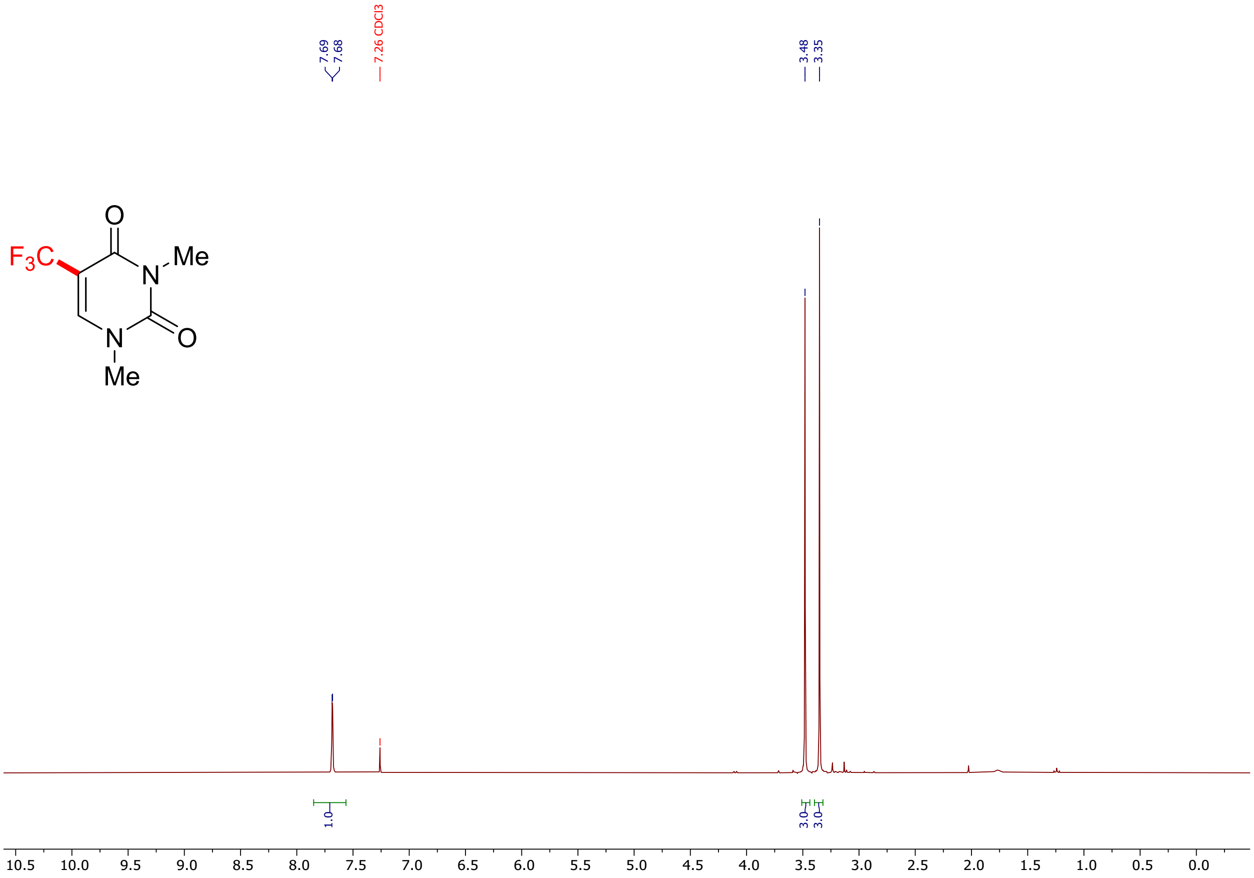

^{13}C NMR spectrum of **10** ($CDCl_3$, 85 MHz)

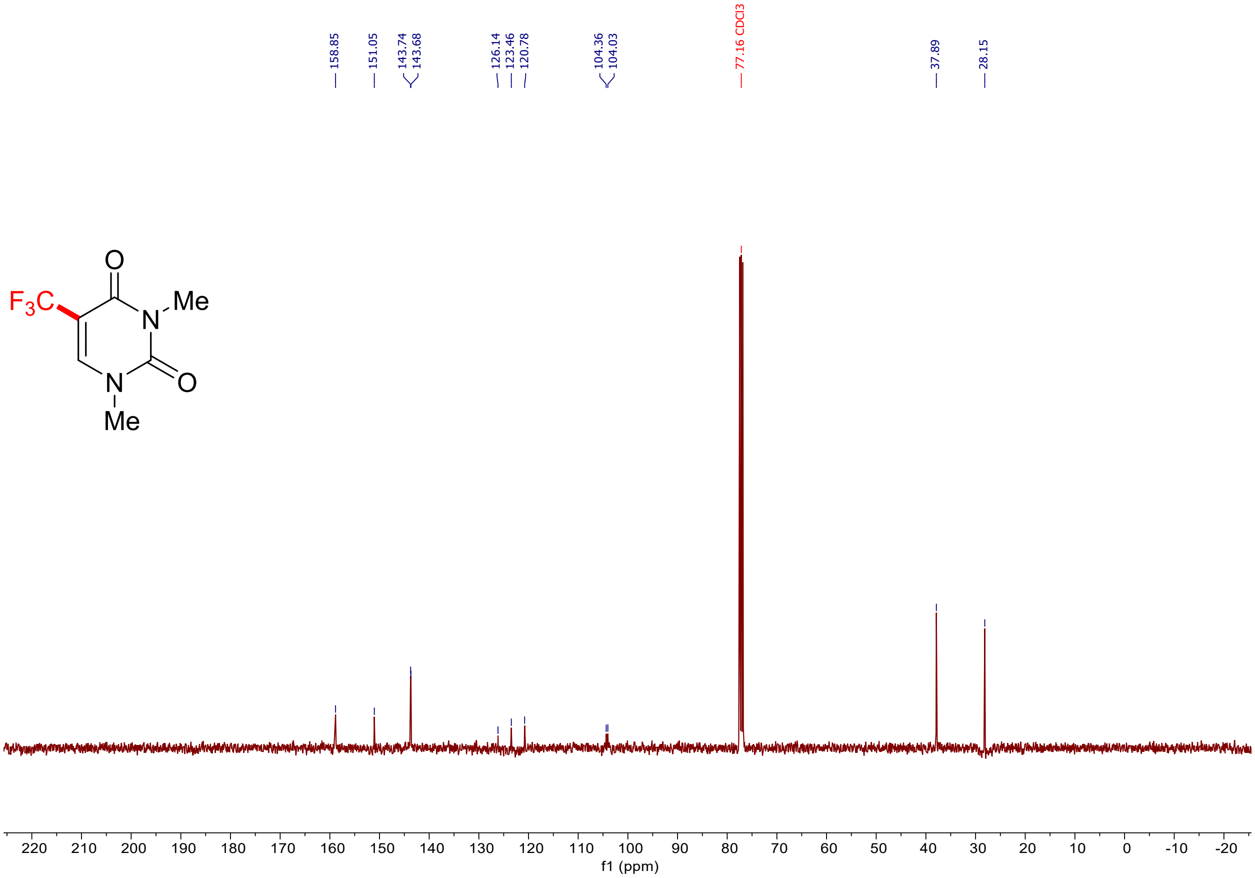

^{19}F NMR spectrum of **10** ($CDCl_3$, 282 MHz)

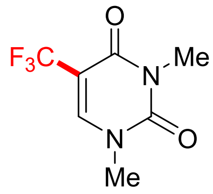

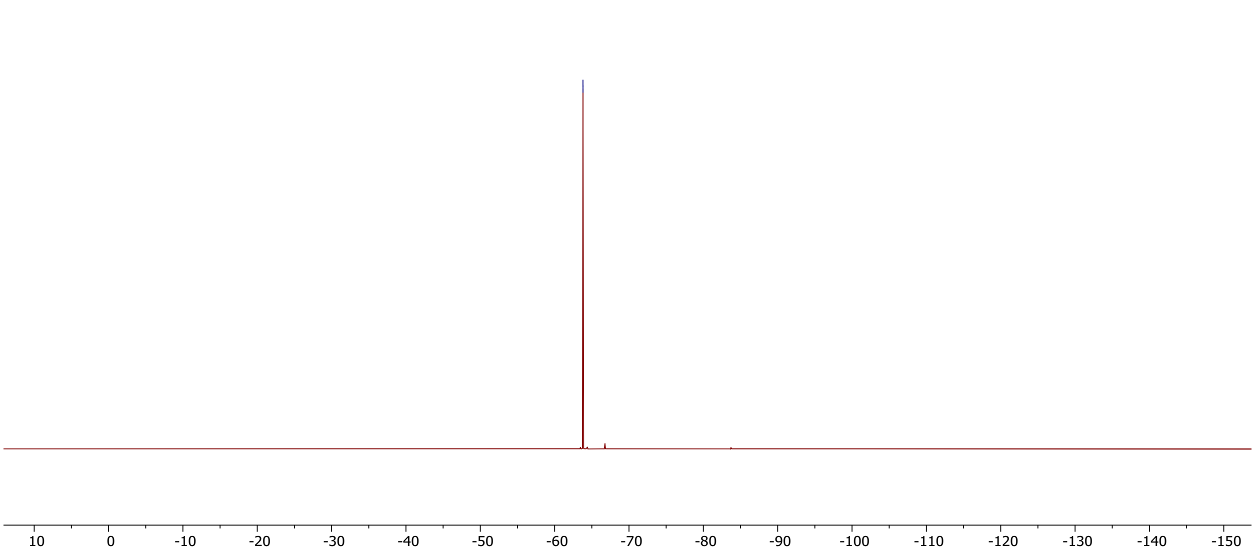

^{1}H NMR spectrum of **11** (MeOD, 300 MHz)

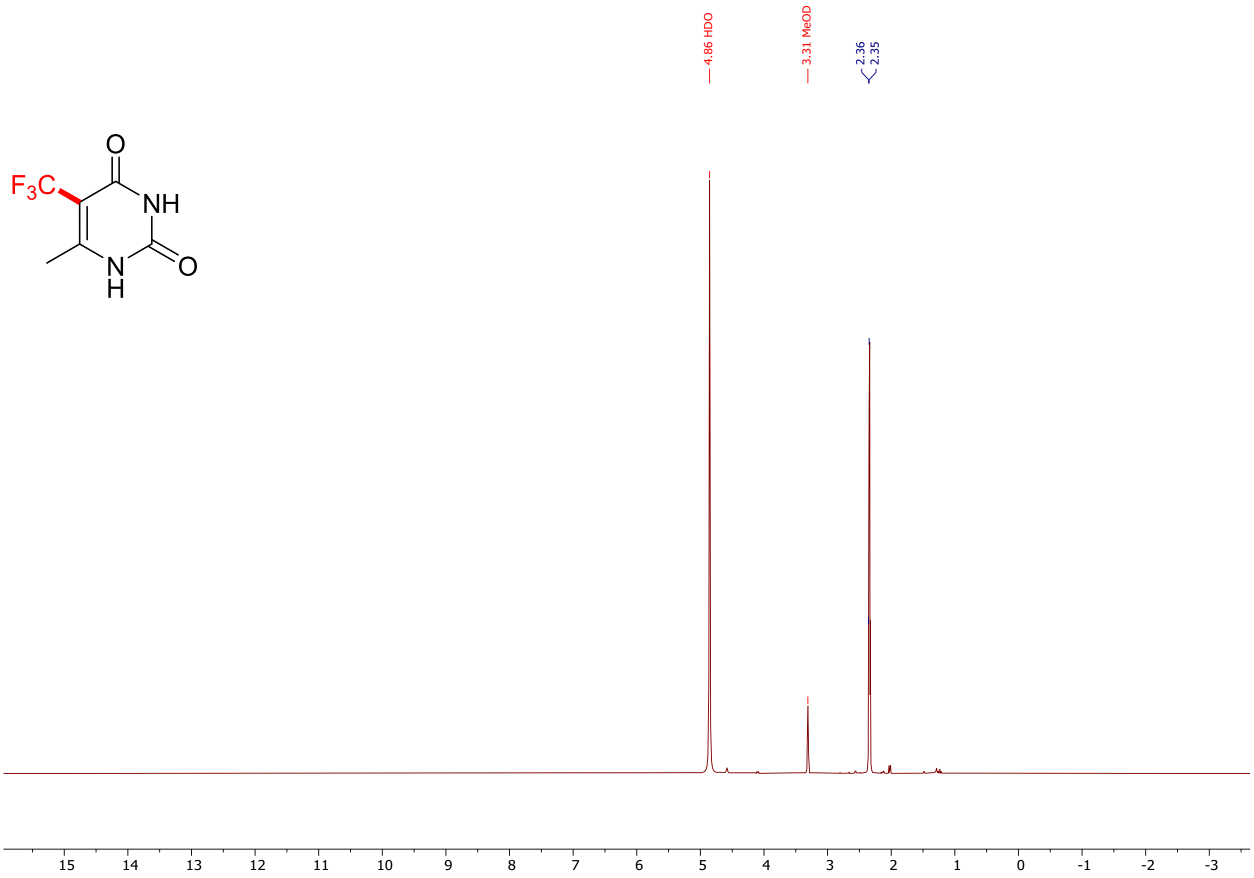

^{13}C NMR spectrum of **11** (MeOD, 75 MHz)

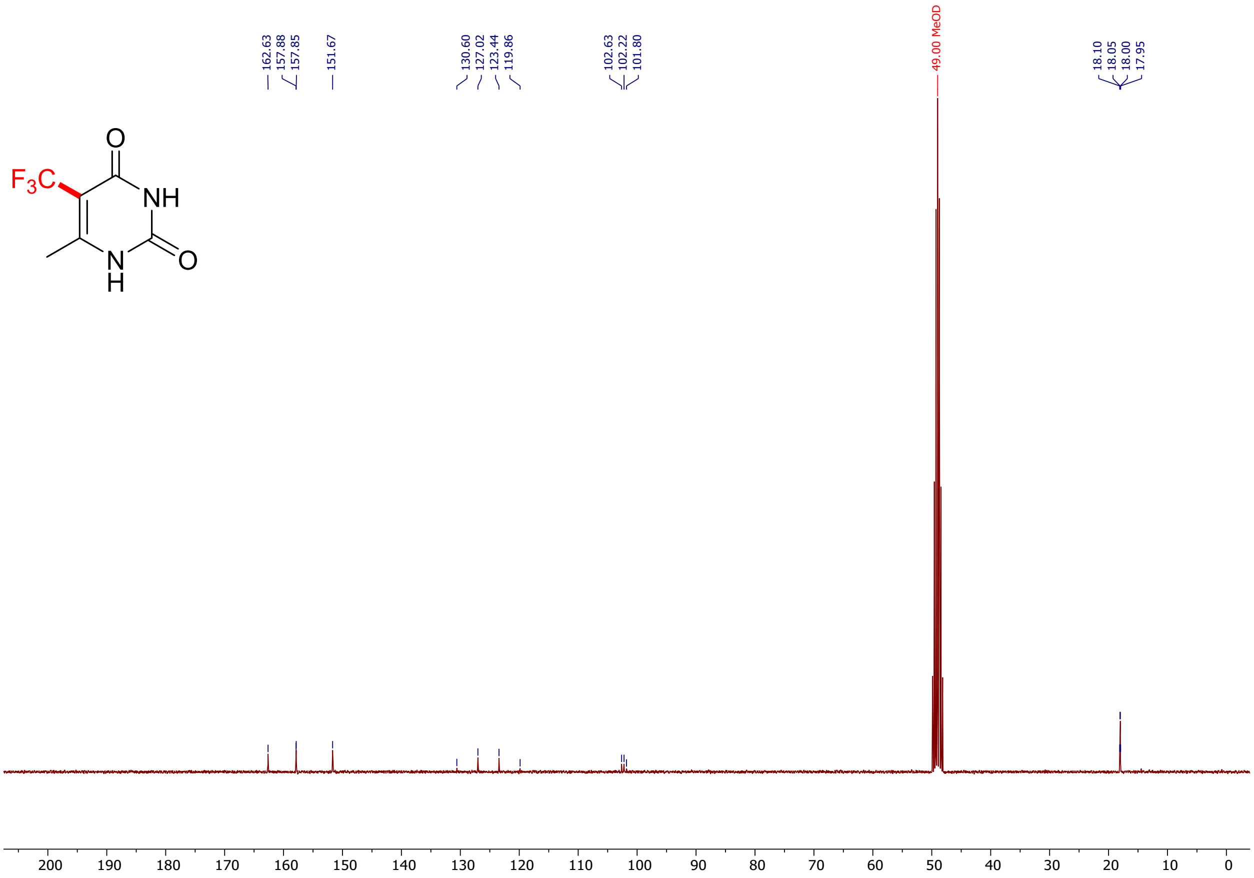

^{19}F NMR spectrum of **11** (MeOD, 282 MHz)

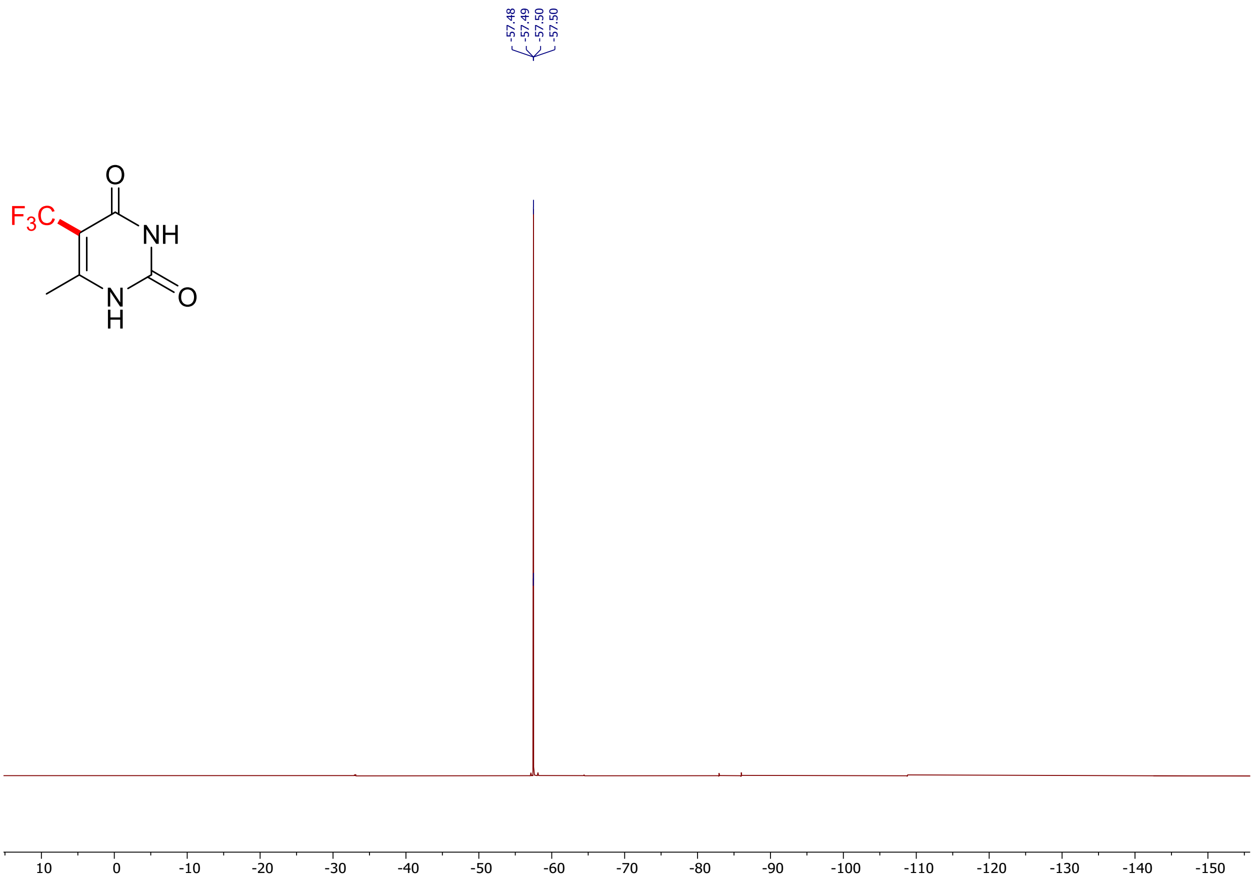

^1H NMR spectrum of **12** (CD_3CN, 300 MHz)

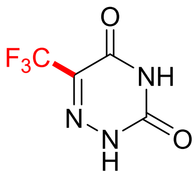

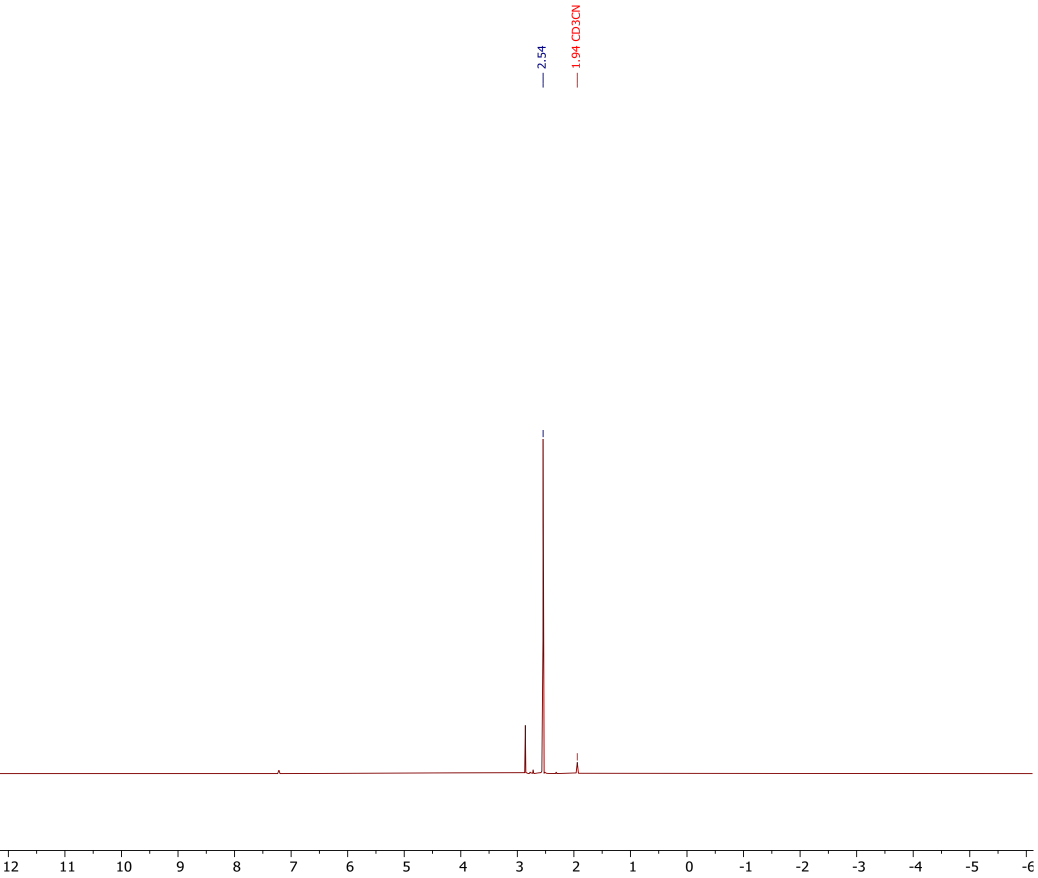

^{19}F NMR spectrum of **12** (CD_3CN, 282 MHz)

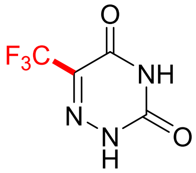

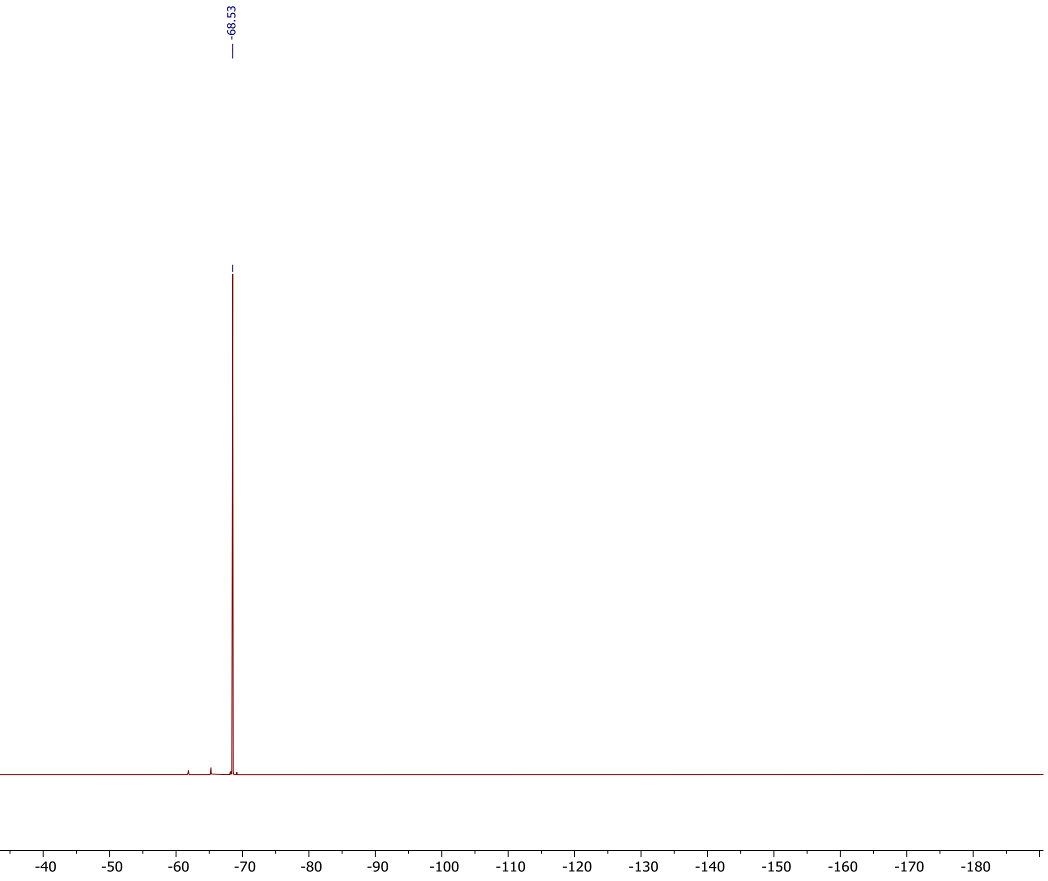

^{1}H NMR spectrum of **13** ($CDCl_3$, 300 MHz)

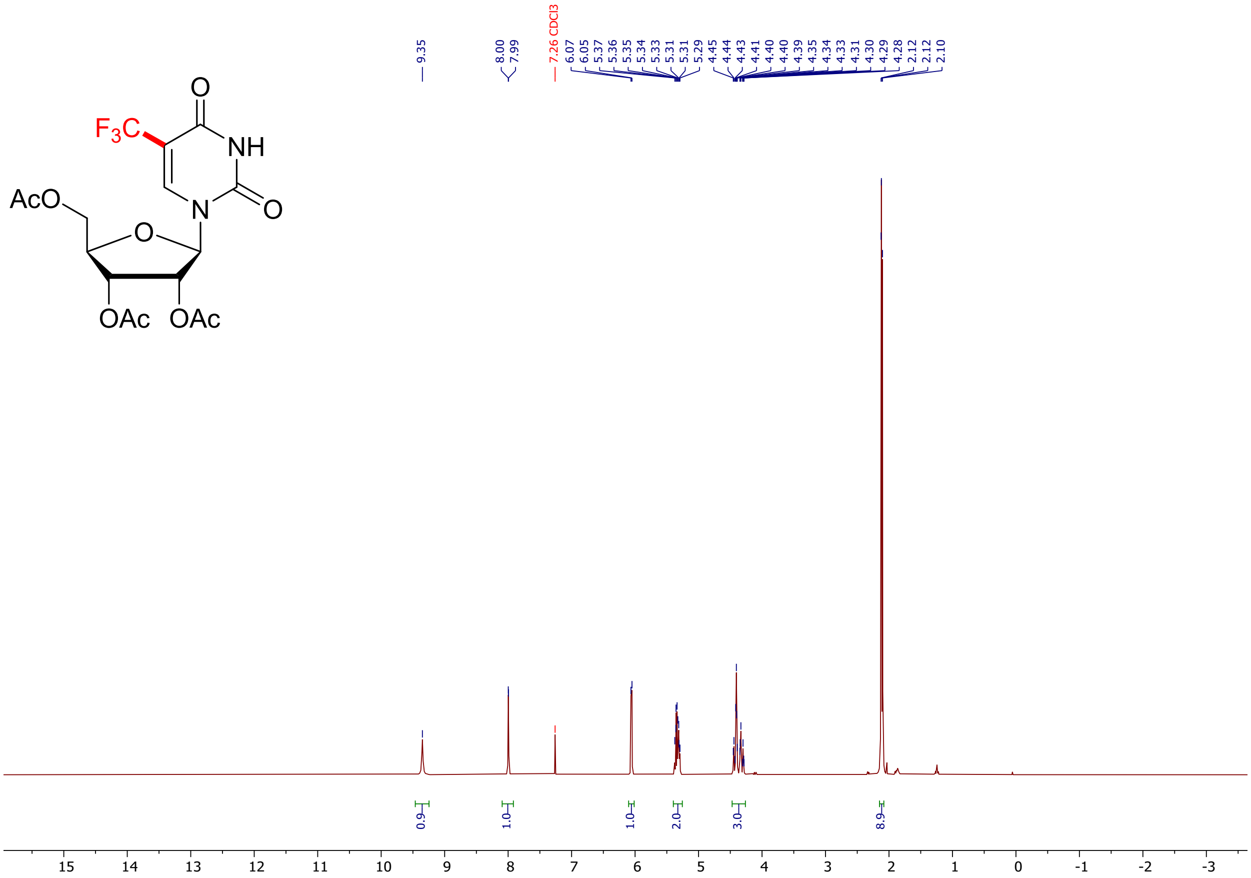

^{19}F NMR spectrum of **13** ($CDCl_3$, 282 MHz)

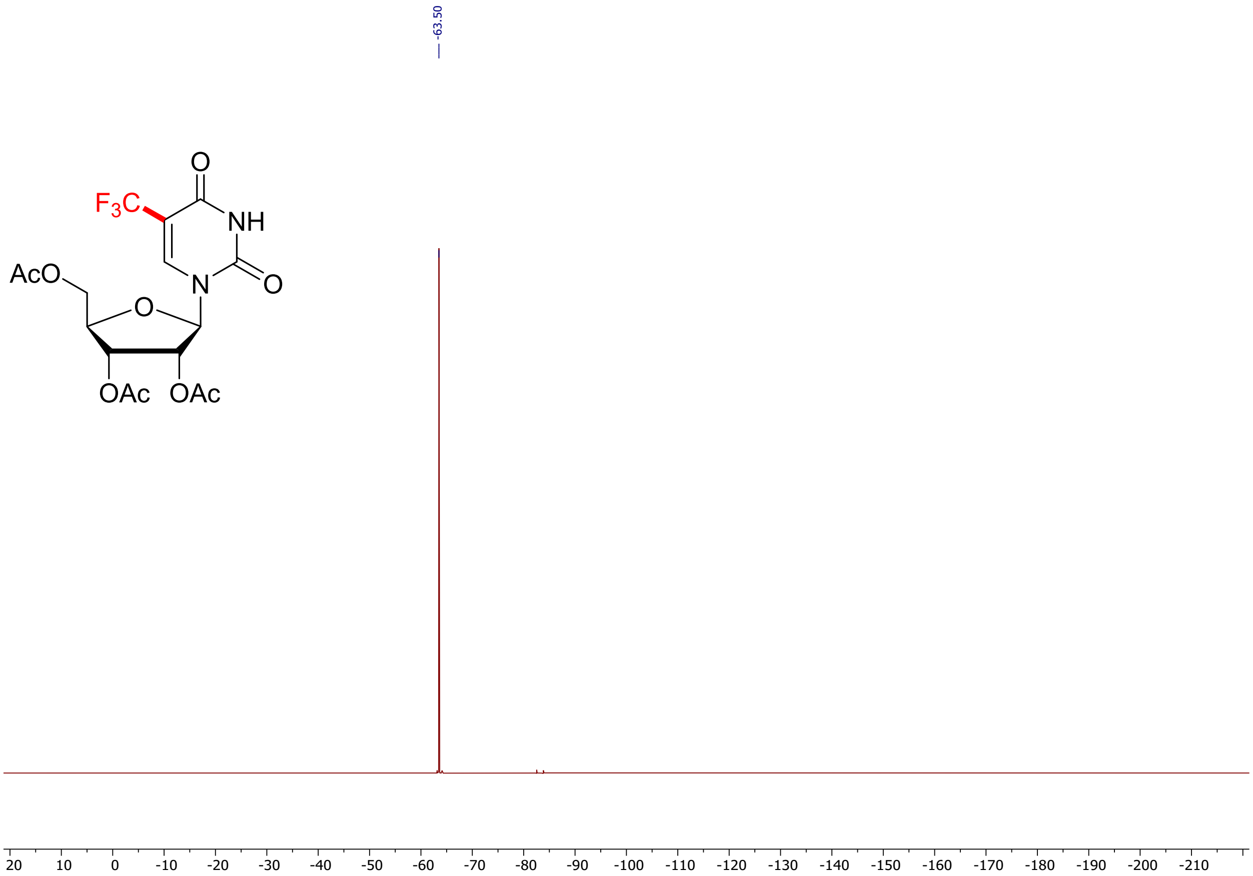

^{1}H NMR spectrum of **14** (MeOD, 300 MHz)

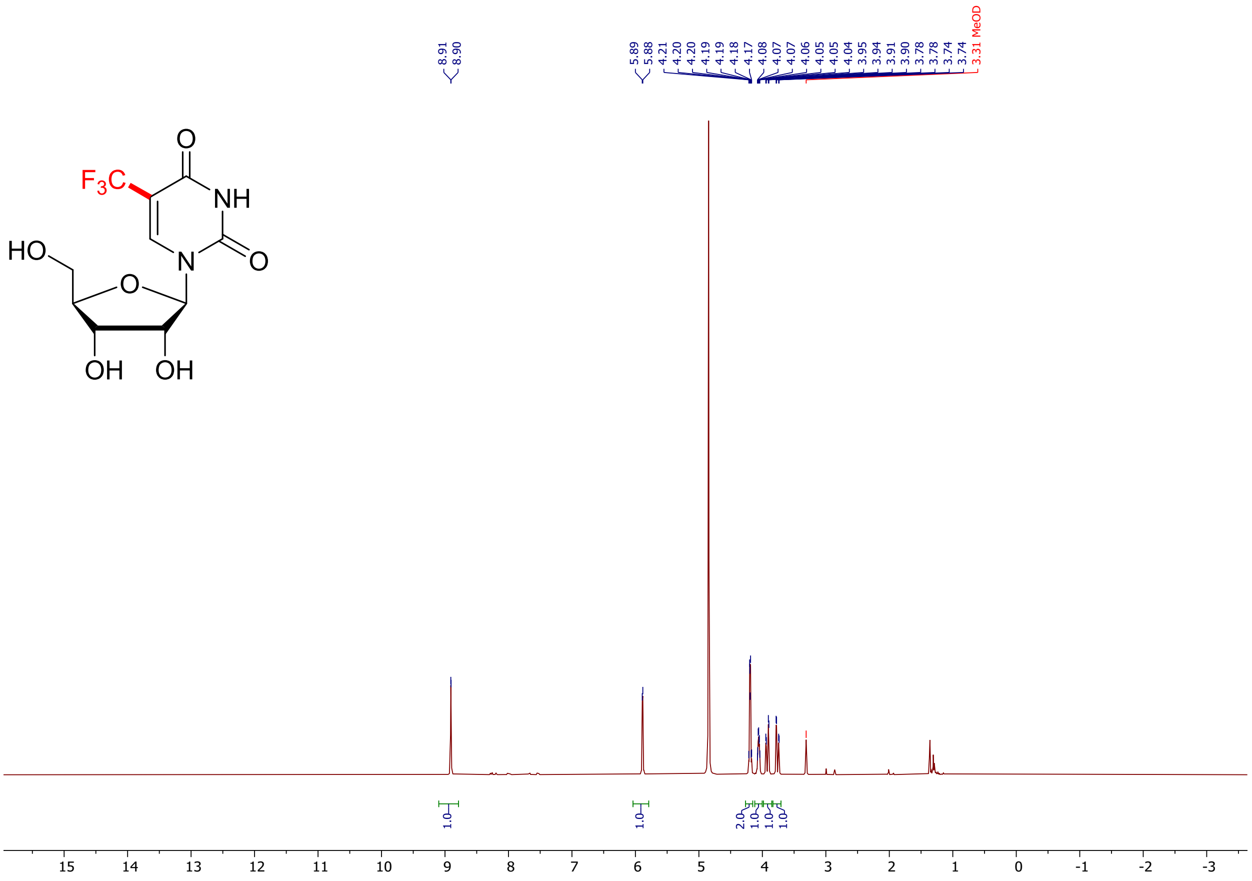

^{13}C NMR spectrum of **14** (MeOD, 75 MHz)

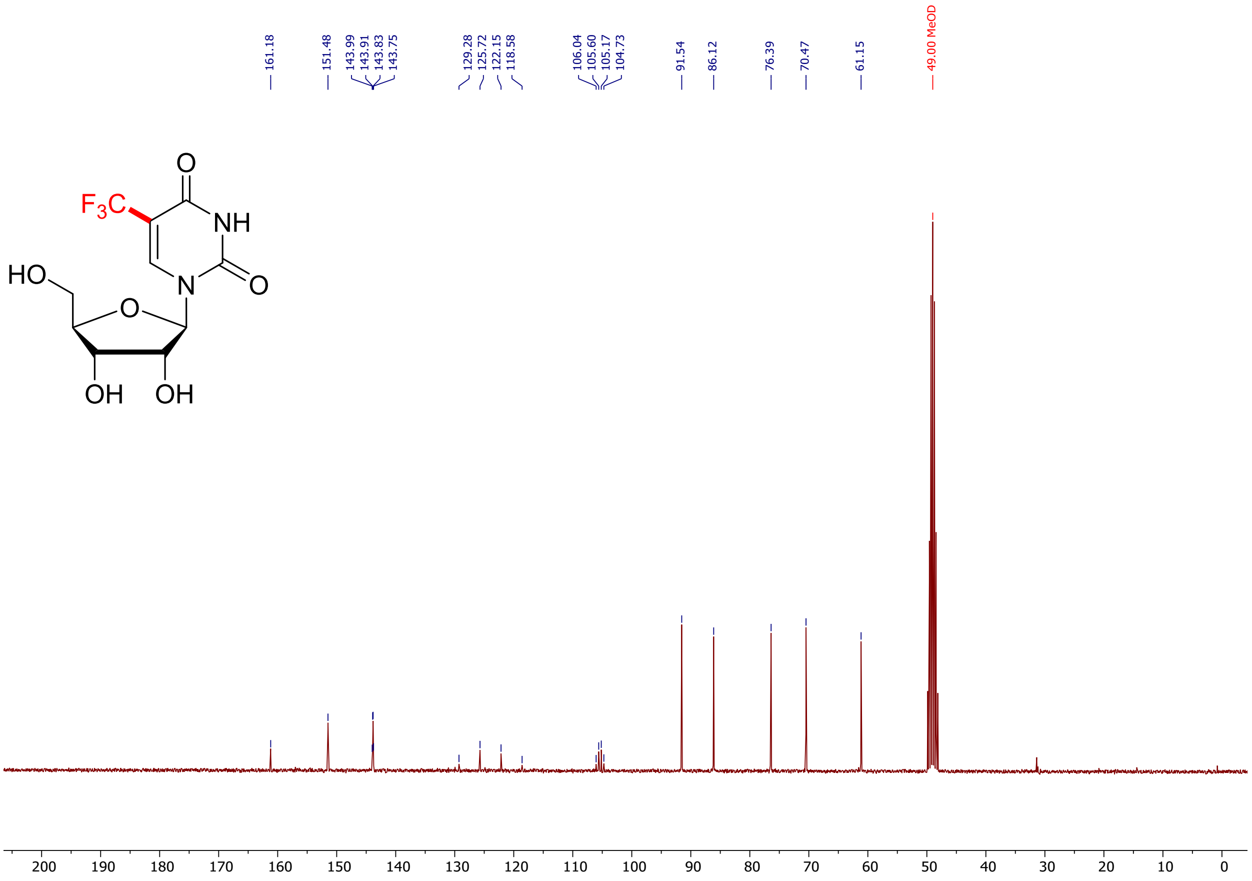

^{19}F NMR spectrum of **14** (MeOD, 282 MHz)

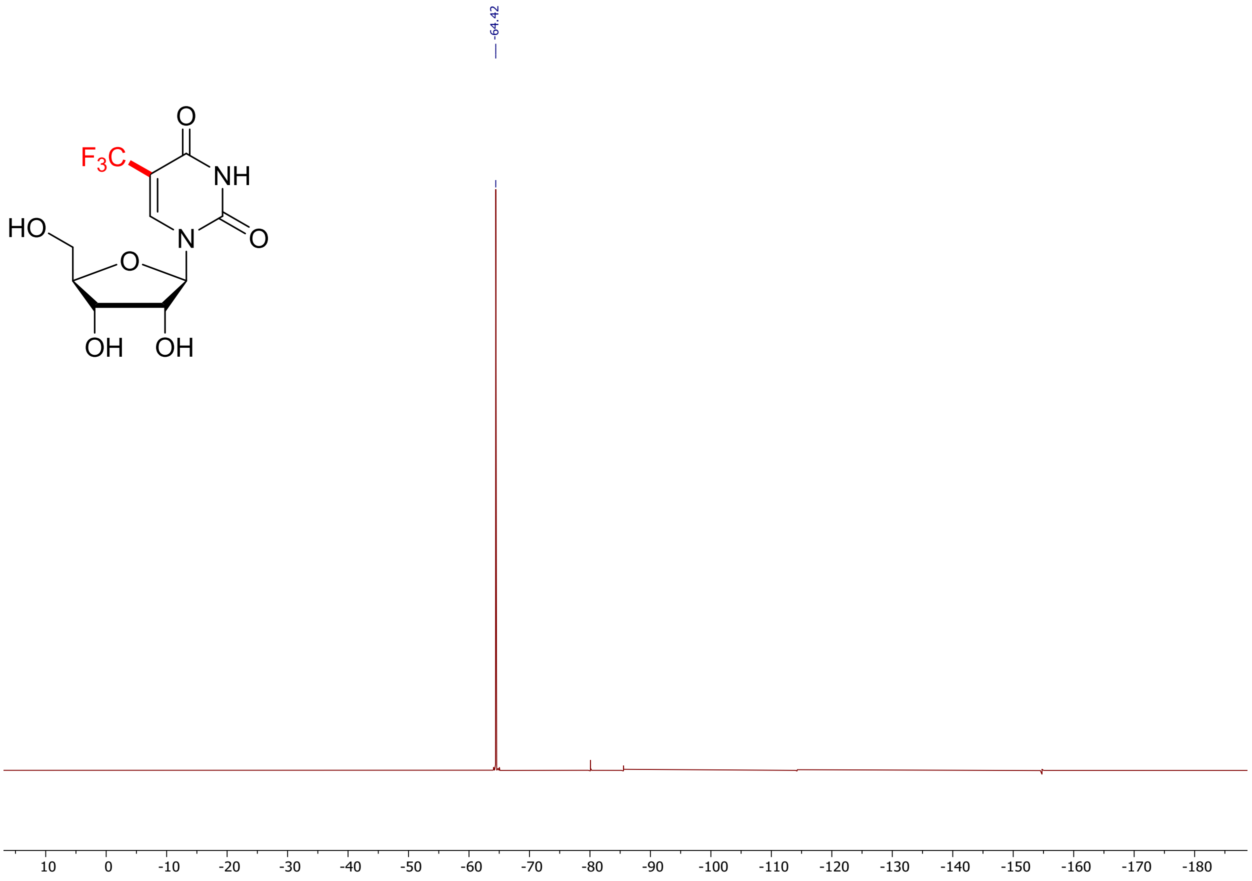

^{1}H NMR spectrum of **15** (DMSO-d_6, 400 MHz)

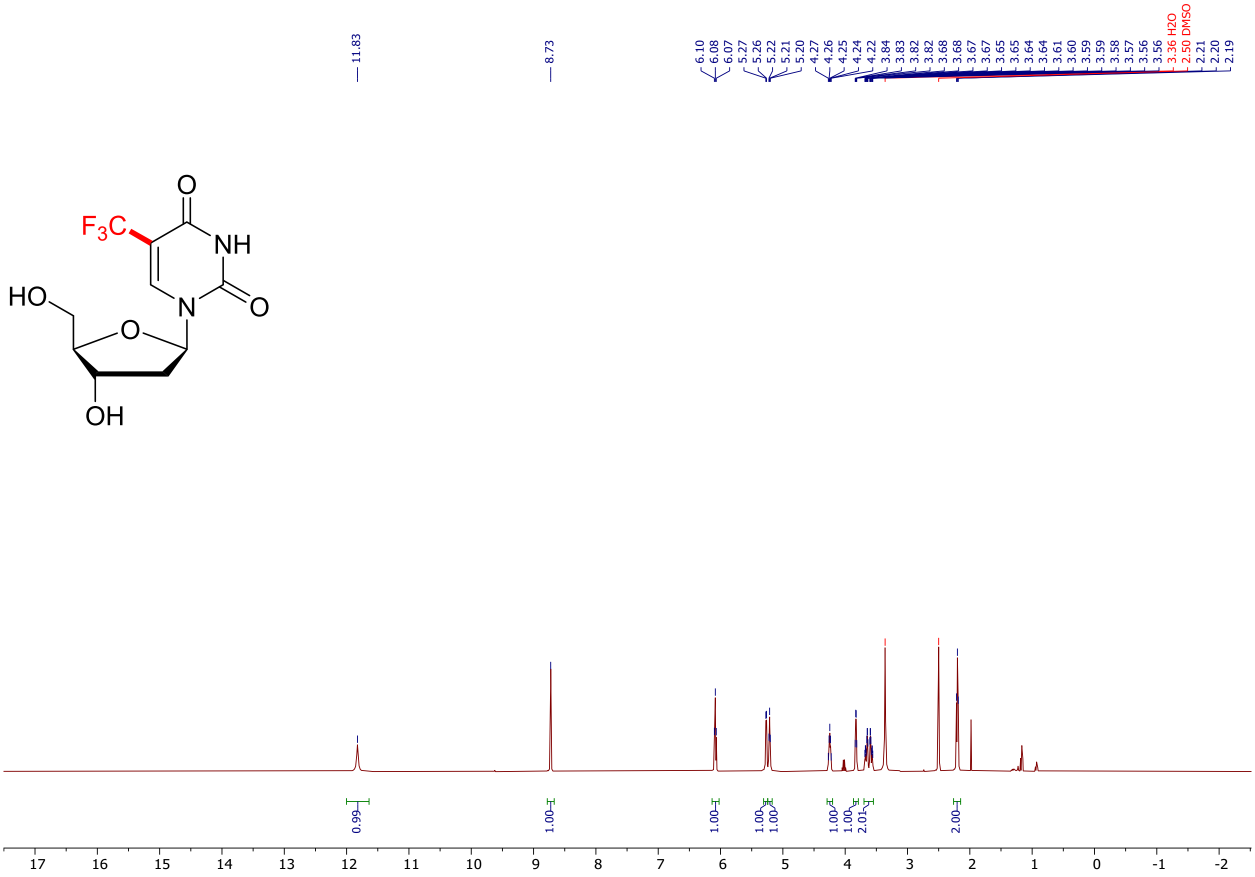

^{13}C NMR spectrum of **15** (DMSO-d_6, 101 MHz)

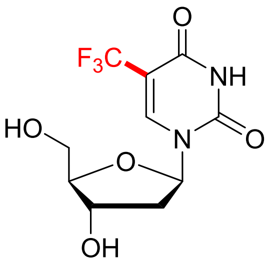

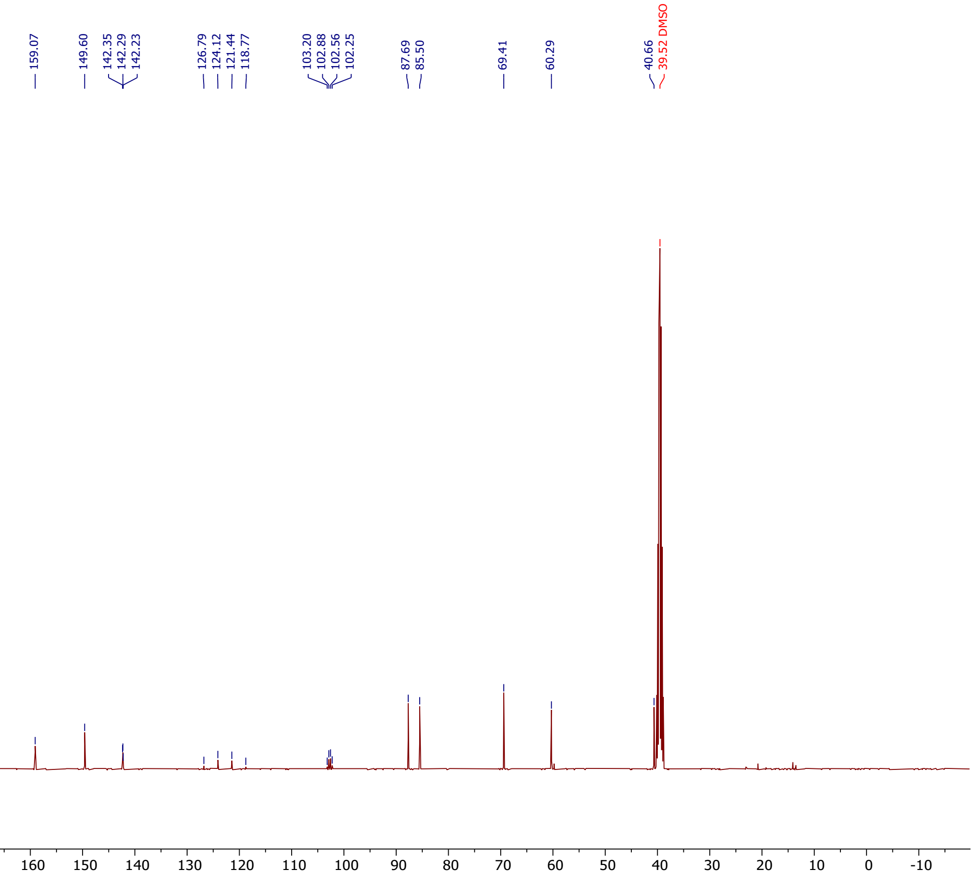

^{19}F NMR spectrum of **9** (DMSO-d_6, 376 MHz)

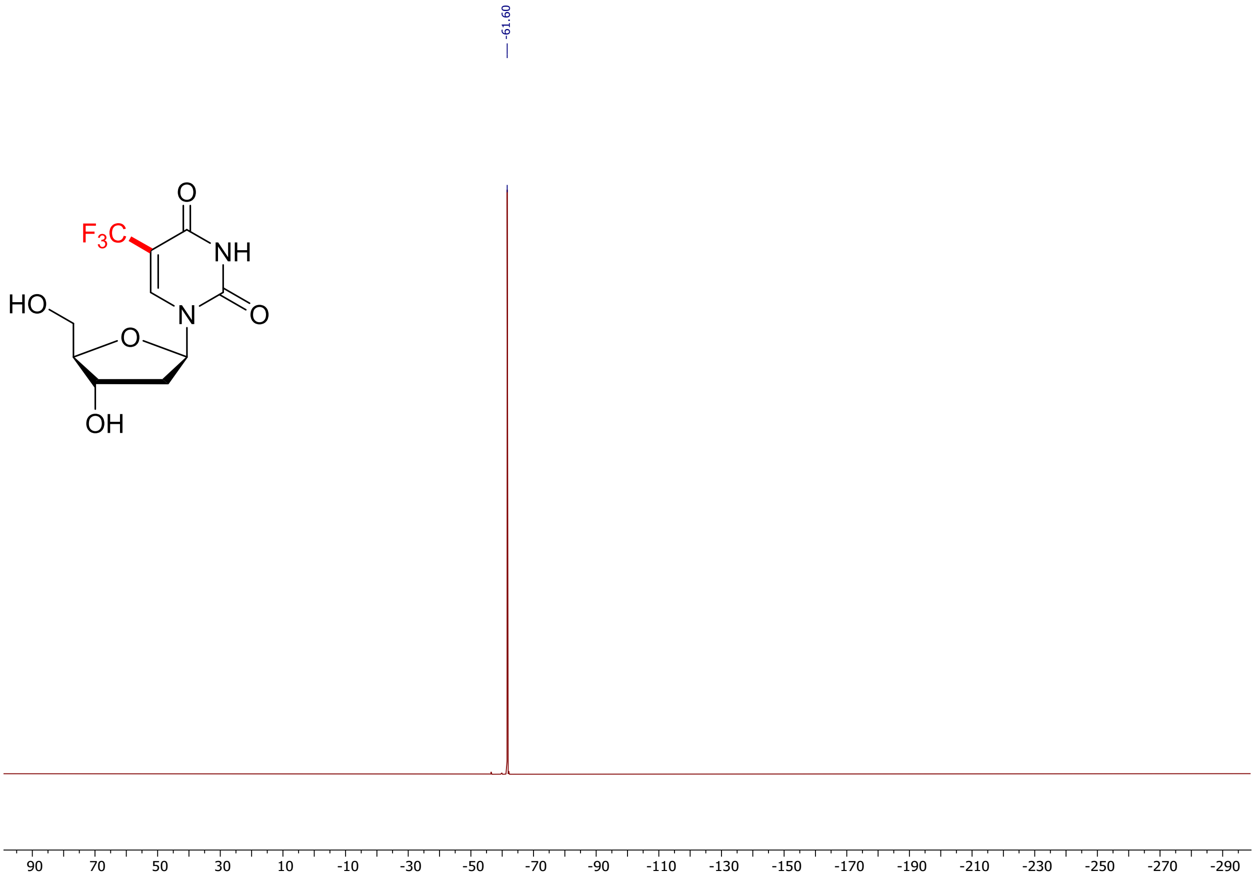

^{1}H NMR spectrum of **16** (C_6D_6, 400 MHz)

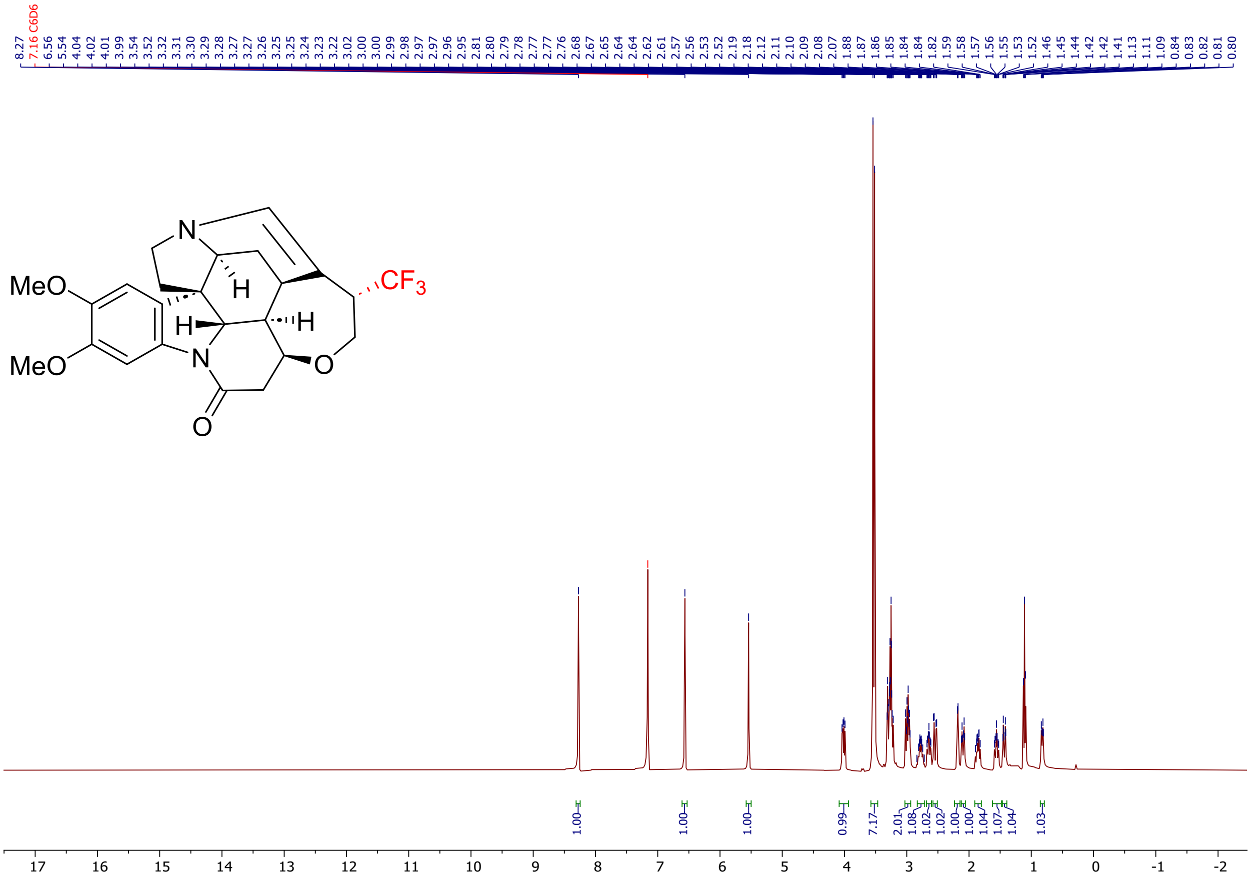

^{13}C NMR spectrum of **16** (C_6D_6, 101 MHz)

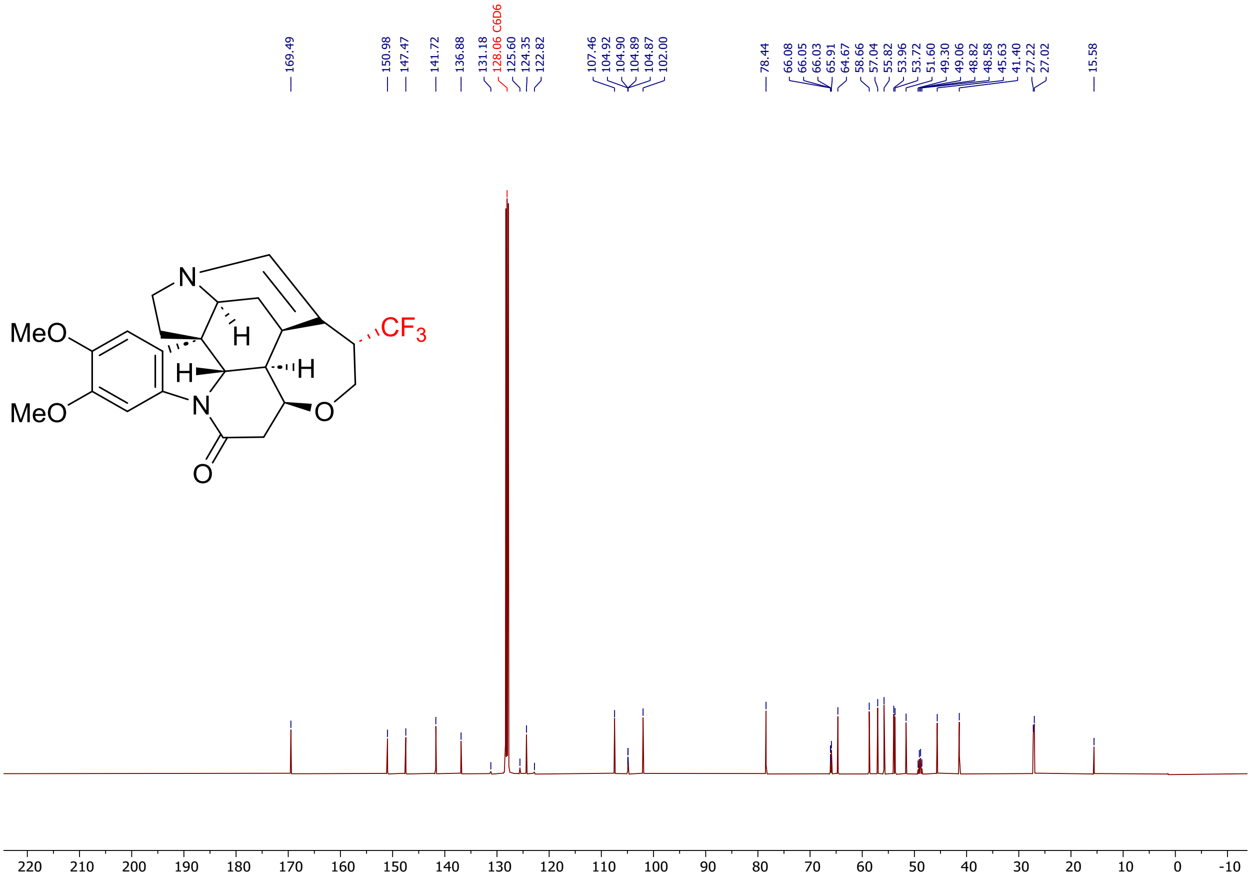

^{19}F NMR spectrum of **16** (C_6D_6, 376 MHz)

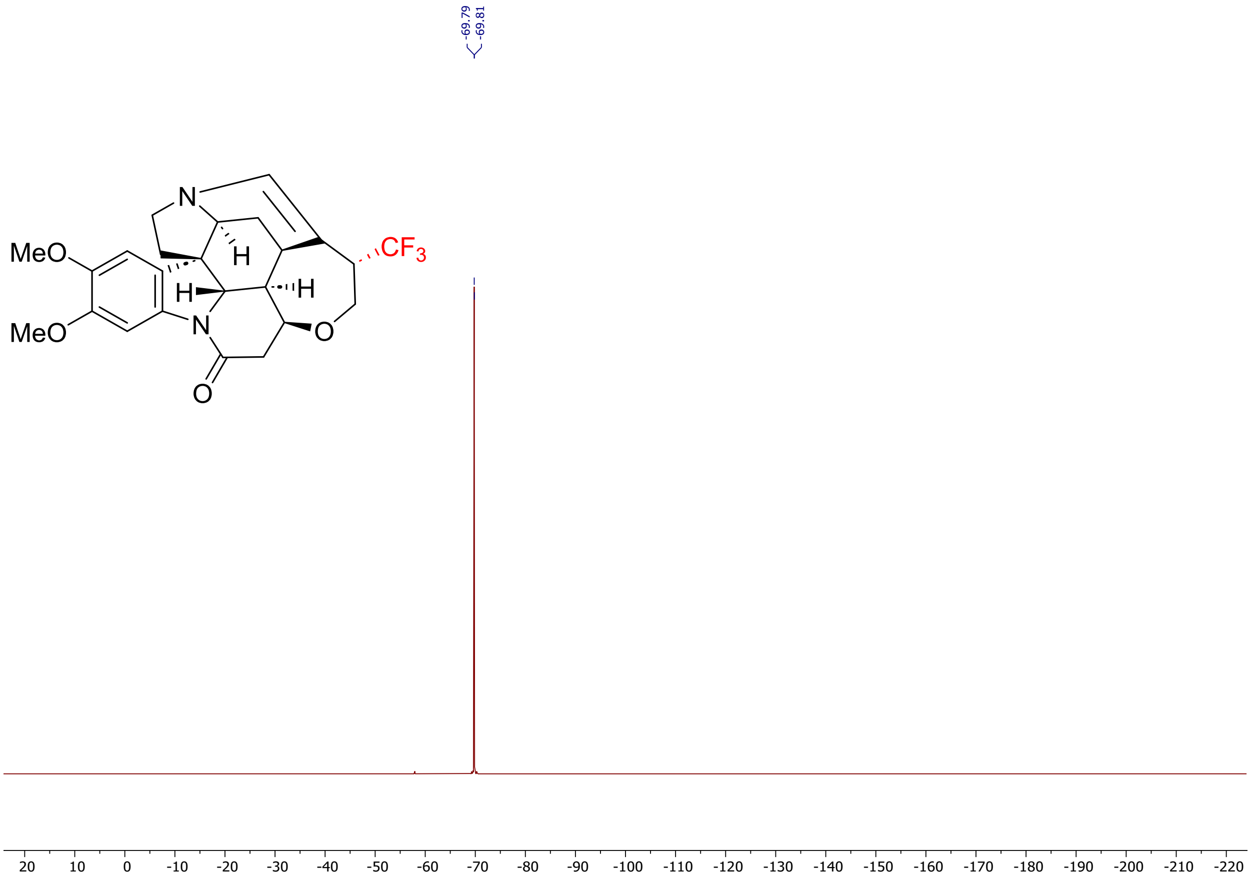

^{1}H–^{1}H COSY of **16** (C_6D_6)

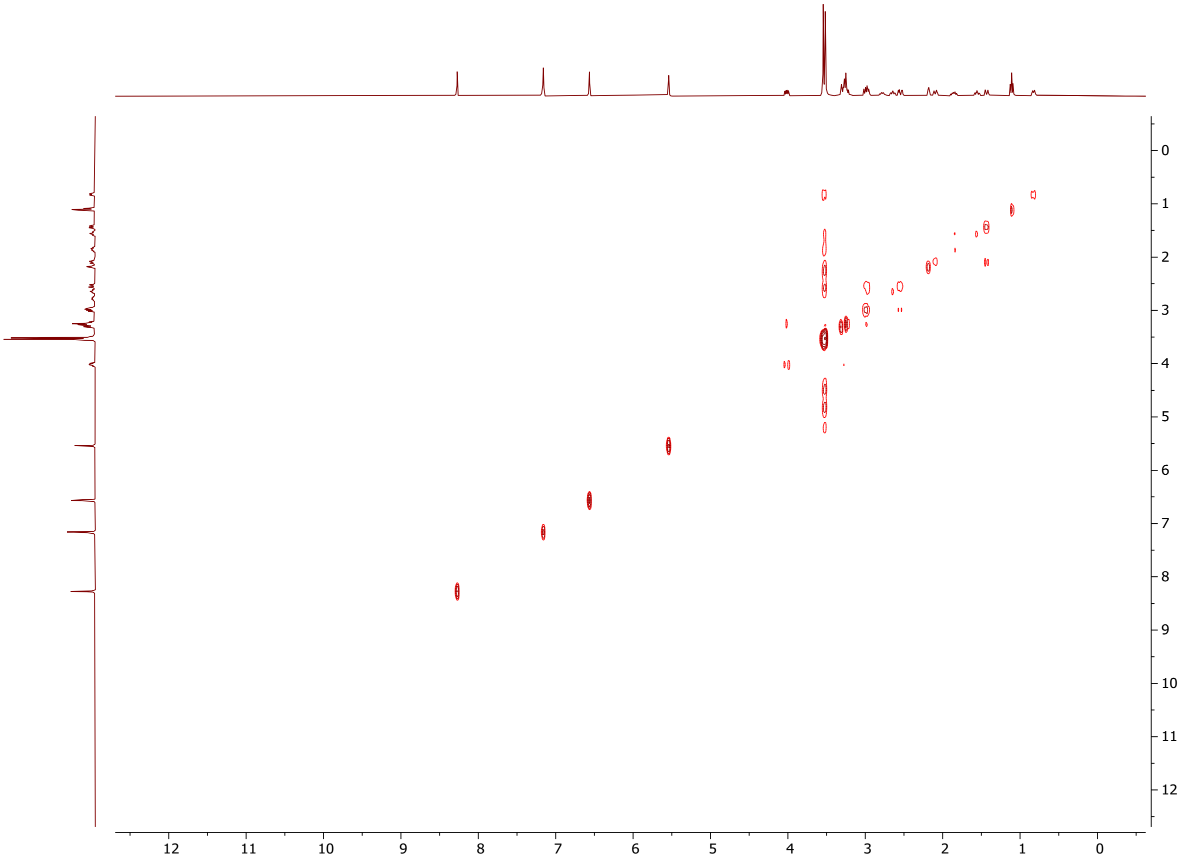

$^{1}H–^{13}C$ HSQC of **16** (C_6D_6)

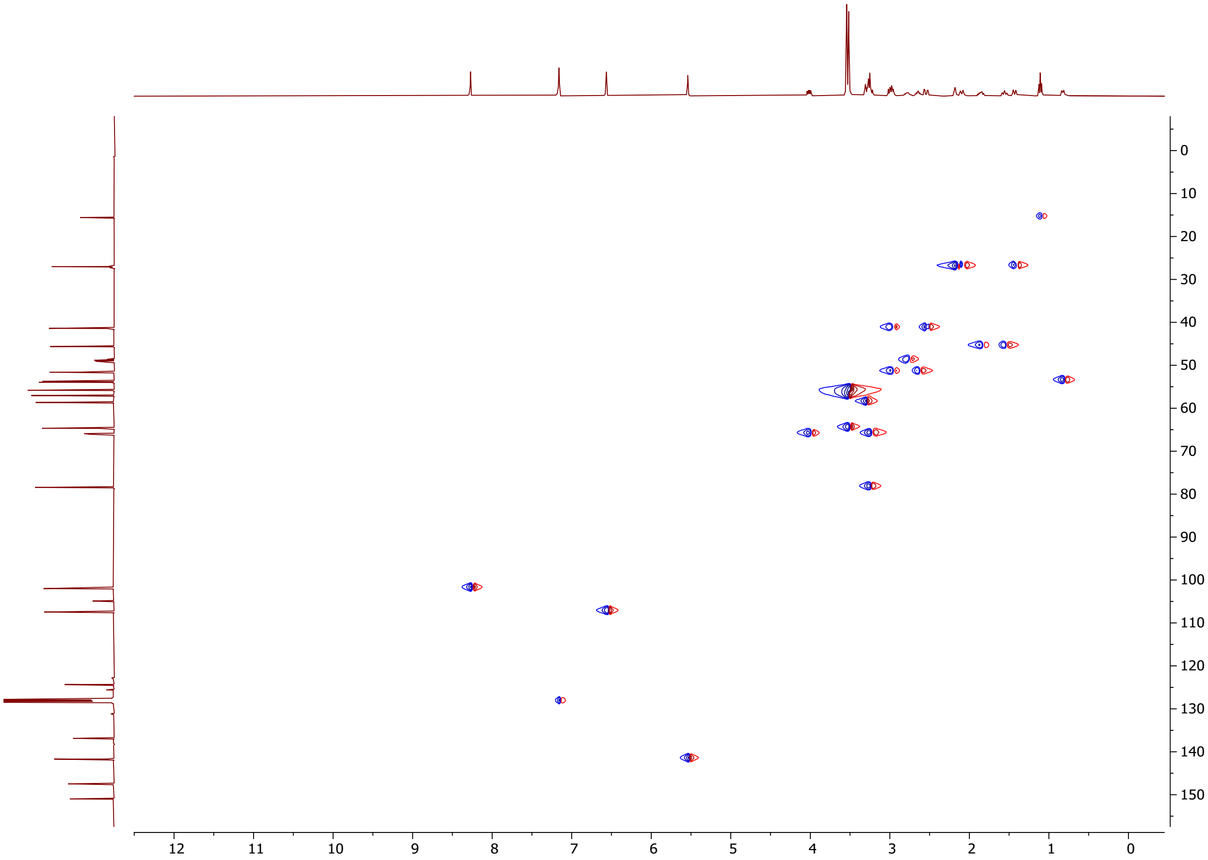

^{1}H-^{13}C HMBC of **16** (C_6D_6)

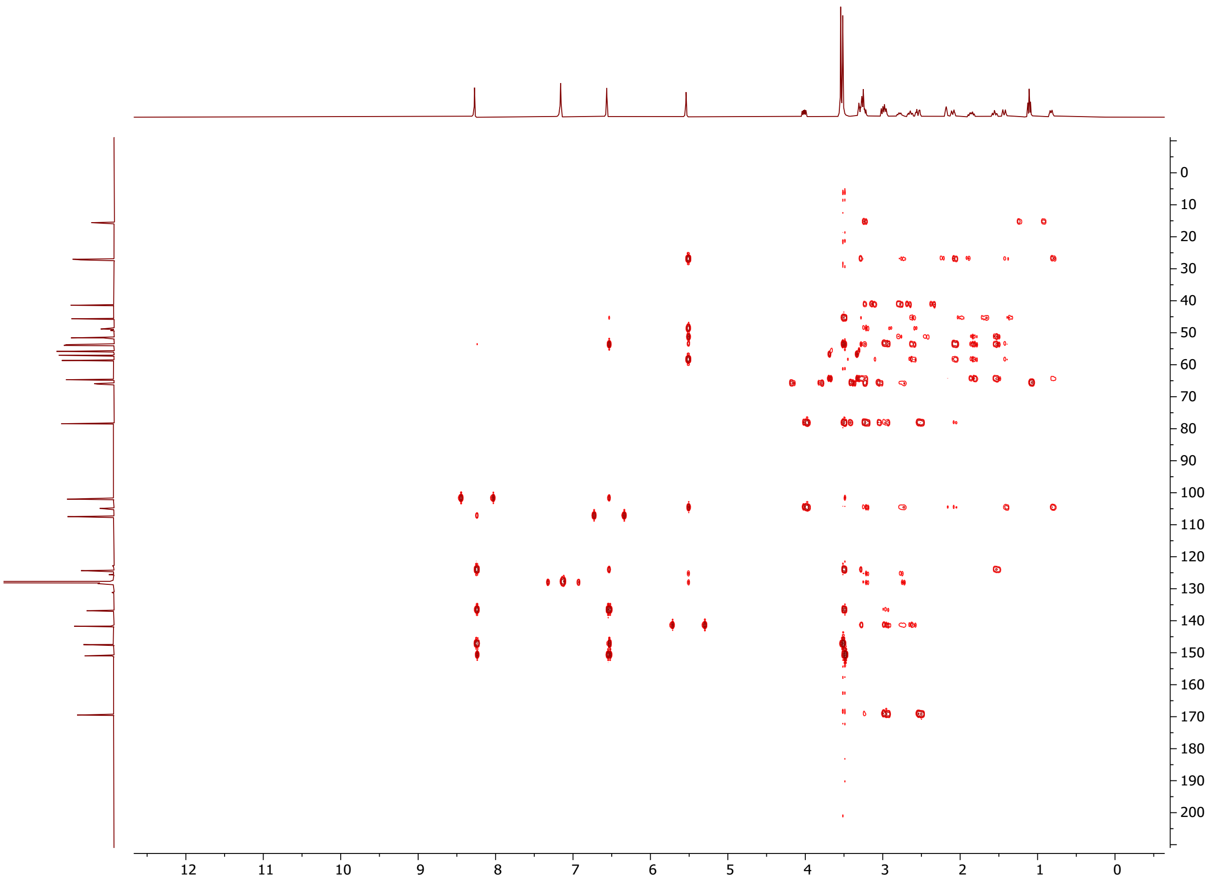

Supplement: SC-017-D5SC07405C-s001 [file SC-017-D5SC07405C-s001.pdf]
